# Effectiveness of an Immersive Telemedicine Platform for Delivering Diabetes Medical Group Visits for African American, Black and Hispanic, or Latina Women With Uncontrolled Diabetes: The Women in Control 2.0 Noninferiority Randomized Clinical Trial

**DOI:** 10.2196/43669

**Published:** 2023-05-10

**Authors:** Suzanne E Mitchell, Alexa Bragg, Barbara A De La Cruz, Michael R Winter, Matthew J Reichert, Lance D Laird, Ioana A Moldovan, Kimberly N Parker, Jessica Martin-Howard, Paula Gardiner

**Affiliations:** 1 Department of Family Medicine and Community Health University of Massachusetts Chan Medical School Worcester, MA United States; 2 Department of Family Medicine Boston Medical Center Boston, MA United States; 3 Department of Family Medicine Boston University School of Medicine Boston, MA United States; 4 Biostatistics and Epidemiology Data Analytics Center Boston University School of Public Health Boston, MA United States; 5 Weatherhead Center for International Affairs Harvard University Cambridge, MA United States

**Keywords:** type 2 diabetes mellitus, virtual world, women, digital health, diabetes self-management education, self-management, health equity, Hispanic or Latina, Black or African American, group visit, shared medical appointment

## Abstract

**Background:**

Medically underserved people with type 2 diabetes mellitus face limited access to group-based diabetes care, placing them at risk for poor disease control and complications. Immersive technology and telemedicine solutions could bridge this gap.

**Objective:**

The purpose of this study was to compare the effectiveness of diabetes medical group visits (DMGVs) delivered in an immersive telemedicine platform versus an in-person (IP) setting and establish the noninferiority of the technology-enabled approach for changes in hemoglobin A_1c_ (HbA_1c_) and physical activity (measured in metabolic equivalent of task [MET]) at 6 months.

**Methods:**

This study is a noninferiority randomized controlled trial conducted from February 2017 to December 2019 at an urban safety net health system and community health center. We enrolled adult women (aged ≥18 years) who self-reported African American or Black race or Hispanic or Latina ethnicity and had type 2 diabetes mellitus and HbA_1c_ ≥8%. Participants attended 8 weekly DMGVs, which included diabetes self-management education, peer support, and clinician counseling using a culturally adapted curriculum in English or Spanish. In-person participants convened in clinical settings, while virtual world (VW) participants met remotely via an avatar-driven, 3D VW linked to video teleconferencing. Follow-up occurred 6 months post enrollment. Primary outcomes were mean changes in HbA_1c_ and physical activity at 6 months, with noninferiority margins of 0.7% and 12 MET-hours, respectively. Secondary outcomes included changes in diabetes distress and depressive symptoms.

**Results:**

Of 309 female participants (mean age 55, SD 10.6 years; n=195, 63% African American or Black; n=105, 34% Hispanic or Latina; n=151 IP; and n=158 in VW), 207 (67%) met per-protocol criteria. In the intention-to-treat analysis, we confirmed noninferiority for primary outcomes. We found similar improvements in mean HbA_1c_ by group at 6 months (IP: –0.8%, SD 1.9%; VW: –0.5%, SD 1.8%; mean difference 0.3, 97.5% CI –∞ to 0.3; *P*<.001). However, there were no detectable improvements in physical activity (IP: –6.5, SD 43.6; VW: –9.6, SD 44.8 MET-hours; mean difference –3.1, 97.5% CI –6.9 to ∞; *P*=.02). The proportion of participants with significant diabetes distress and depressive symptoms at 6 months decreased in both groups.

**Conclusions:**

In this noninferiority randomized controlled trial, immersive telemedicine was a noninferior platform for delivering diabetes care, eliciting comparable glycemic control improvement, and enhancing patient engagement, compared to IP DMGVs.

**Trial Registration:**

ClinicalTrials.gov NCT02726425; https://clinicaltrials.gov/ct2/show/NCT02726425

## Introduction

Minority and low-income women with type 2 diabetes mellitus (T2DM) face widening disparities in diabetes care and clinical outcomes, highlighting the pressing need to improve diabetes care for underserved communities [1-5]. Diabetes medical group visits (DMGVs) are shared appointments where groups of patients receive diabetes self-management education (DSME), peer support, and a clinical visit within a 2-hour appointment. Compared to usual care for adults living with diabetes, the in-person (IP) DMGV model has been associated with improved diabetes outcomes and lower costs [6-9]. Moreover, receiving care as a group can reduce disparities by fostering more equitable patient-provider relationships, creating relationships of care between patients, and improving health literacy and self-management skills [10]. Yet, many disadvantaged communities report poor access to DMGVs as health systems find them difficult to implement [11,12]. Patient engagement in group-based diabetes care is also often low due to social stigma, lack of transportation, and time constraints [11,13].

Telehealth solutions have gained unprecedented traction with the onset of the COVID-19 pandemic. Early evidence has shown that virtual worlds (VWs) and virtual reality platforms are feasible and potentially more effective alternatives to IP programming [14,15]. A VW is a 3D, computer-based simulated environment where users engage in immersive, experiential learning with animated educational content [16]. Users create avatars, digital manifestations of self, to engage in peer group programming virtually [17]. This environment is intrinsically designed for users to enact behavioral change among peers and restructure old habits [18,19].

To our knowledge, the possibilities of avatar-based VW DSME have not been rigorously tested. We developed an immersive telemedicine platform, linking an interactive VW learning environment with videoconferencing software, to overcome the common barriers to diabetes group-based care while maintaining clinical effectiveness at scale. We implemented Women in Control 2.0 (WIC2) in 2015 to study the comparative effectiveness of delivering DMGVs in a VW versus the traditional IP classroom for women from Black, African American, Hispanic, or Latina backgrounds with uncontrolled T2DM (trial protocol in Multimedia Appendix 1) [20].

## Methods

### Trial Design

From February 2017 to October 2019, we recruited 17 cohorts of African American or Black or Hispanic or Latina women with uncontrolled T2DM. A total of 309 participants were enrolled and randomly assigned to the VW or IP DMGV conditions. Participants attended 8 weekly DMGVs and were followed for 6 months.

### Participants

Eligible participants were adult women (≥18 years) who self-identified as African American, Black, Hispanic, or Latina with uncontrolled T2DM, defined by a hemoglobin A_1c_ (HbA_1c_) value ≥8%. Participants were English-speaking or Spanish-speaking, had telephone access, permanent or stable housing, a clinician-supervised diabetes treatment plan, and could provide informed consent. Exclusion criteria included scheduling conflicts with DMGV programming, enrollment in another program, a history of diabetic ketoacidosis, oxygen-dependent chronic obstructive pulmonary disease, stroke within the last 6 months, and an acute coronary event or chronic heart condition within the last year. Pregnancy, recent glucocorticoid therapy, dialysis, active substance abuse, active cancer treatment, and any medical contraindications to study dietary recommendations were also exclusionary.

### Recruitment

#### Overview

We identified participants from Boston Medical Center and a local community health center using a weekly electronic medical record query. We contacted eligible participants with an introductory letter and follow-up call [21]. Additional recruitment strategies included participant or provider referrals and posted flyers. We screened participants by phone and reconfirmed eligibility at an IP enrollment appointment. Participants provided written informed consent and were eligible for up to US $300 in compensation or a new laptop.

#### Randomization and Masking

After stratification by language, we used one-to-one block randomization (alternating blocks of 6 and 8) to assign participants to the VW or IP DMGV conditions. A biostatistician generated the randomization sequence, and randomization occurred after informed consent. We randomized participants prior to obtaining baseline data. Investigators were blinded to the randomization process, but assignments were revealed to participants and investigators post consent.

### Intervention

Assigned to cohorts of 6-12 participants based on study arm, participants convened in clinical or virtual settings for 8 weekly DMGVs. Each session lasted approximately 120 minutes and started with the completion of an intake form to document acute or chronic symptoms, health system usage, and self-management activities, followed by the measurement of vital signs and the delivery of DSME. Sessions included a one-on-one clinical consult. Study clinicians were 4 board-certified physicians and 2 nurse practitioners. Nonclinical group facilitators received training on core DSME topics and facilitation skills from lead faculty (SEM, PG). All participants received a paper curriculum booklet.

**Figure 1:**
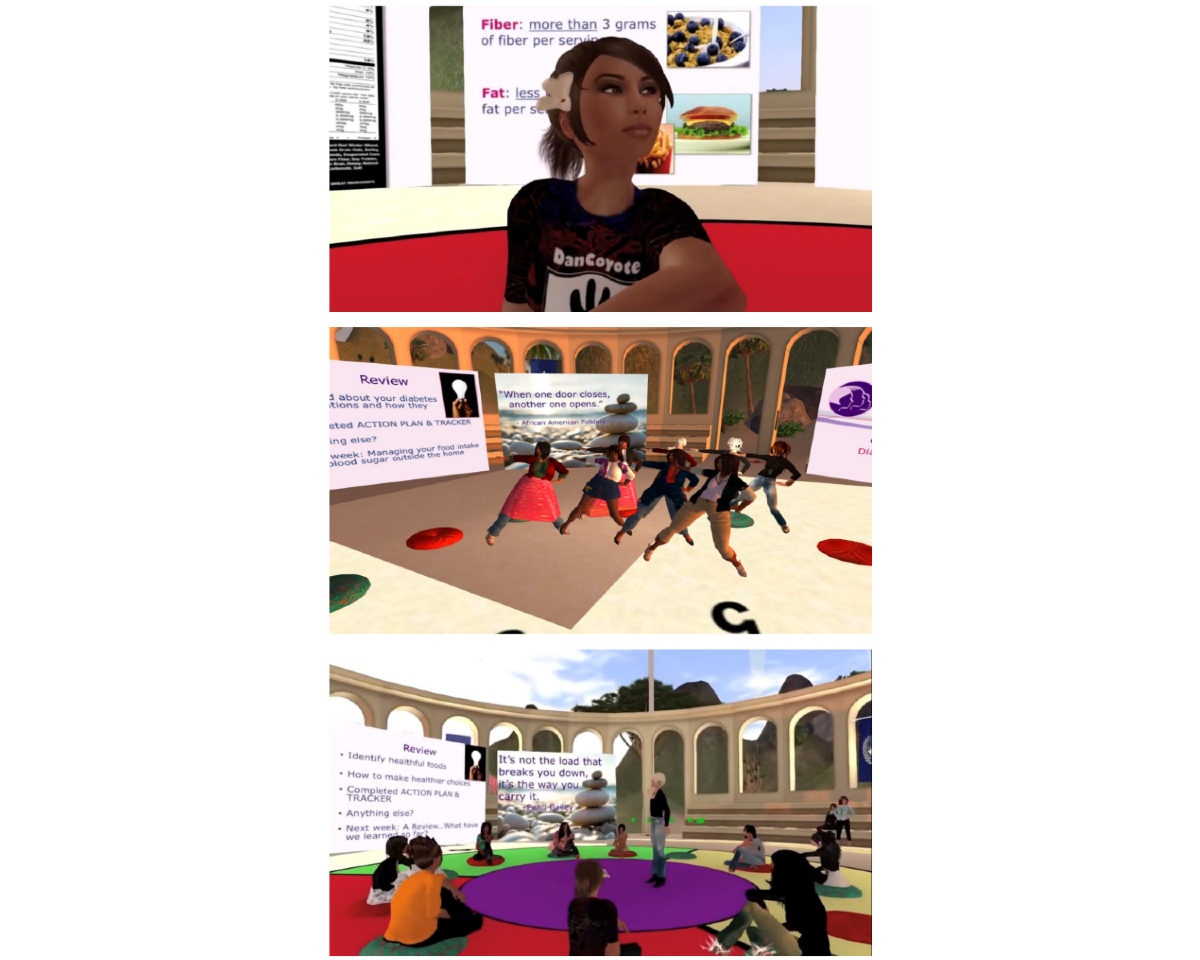
Illustration of avatars in the virtual world.

Prior to the first virtual DMGV, staff provided laptops and wireless internet to VW participants and conducted IP computer training. All participants then met weekly for 8 weeks, according to a session schedule. During DMGVs, all participants received the same WIC2 curriculum, which was adapted from *Power to Prevent* [22] and consisted of 8 modules highlighting topics such as diabetes self-monitoring, preventative care, healthy eating, exercise, and stress management. Three bilingual staff, who were native Spanish speakers and included a certified interpreter, used the forward-backward method of translation, in tandem, to produce a culturally equivalent, Spanish-language curriculum [23,24]. The curriculum content was reviewed by 2 patient advisory groups. To develop avatar-driven learning experiences and incorporate chat and telehealth capabilities, instructional design was adapted for a VW environment using game design theory [25]. VW participants customized avatars to represent themselves and engage in DMGVs, including practicing positive health behaviors such as dance and social support (Figure 1).

During each session, a clinician met individually with participants (in a separate physical space or via secure telehealth platform or telephone depending on study group) to review blood glucose readings and hyper or hypo glycemic data, conduct diabetes medication reconciliation, and address concerns. Recommendations for medication adjustments were based on an algorithm [26] and shared with primary care providers via progress notes in the electronic health record.

To ensure fidelity of DMGV protocols and standard operating procedures, we used checklists, audits of session recordings, and participant observation field notes.

Following the 8-week DMGV sessions, participants entered a 16-week maintenance period. They were encouraged to self-monitor (tracking blood glucose, blood pressure, diet, and exercise) using a paper booklet or mobile app. No formal DMGVs occurred.

### Outcomes

The primary outcomes were mean changes in (1) HbA_1c_ and (2) physical activity (metabolic equivalent of task [MET]-hours) by study arm from baseline to 6 months. Secondary outcomes included mean changes in HbA_1c_, physical activity, and medication changes at 9 weeks after enrollment and changes in the 17-item Diabetes Distress (DD) scale, depressive symptoms (Patient Health Questionnaire-8 [PHQ-8]), physical function (Patient-Reported Outcomes Measurement Information System [PROMIS-29] measure), 13-item Patient Activation Measure (PAM), weight, and step count at 6 months [27-31].

### Data Collection and Management

Baseline data collection included sociodemographic characteristics, HbA_1c_ values (blood draw), physical activity (per accelerometers), and survey measures. Baseline HbA_1c_ was obtained within 30 days of the first DMGV. Physical activity was measured within a 14-day window, with participants wearing an accelerometer on the wrist for 7 consecutive days. Follow-up data collection occurred 9 weeks and 6 months post enrollment (within a 28-day window). Study data were stored using secure Research Electronic Data Capture (REDCap) software hosted by Boston University [32,33]. Unique study identification numbers were used to label all participant forms.

### Sample Size Calculations

We used the average overtime change in HbA_1c_ and physical activity by study arm as coprimary outcomes, measured from baseline to 6-month follow-up. Data obtained from the WIC 1.0 pilot study was used to estimate the sample size necessary to establish the noninferiority margin of VW DMGVs compared to IP DMGVs at reducing HbA_1c_ and increasing total physical activity levels [34]. For HbA_1c_, we assumed a noninferiority margin of 0.7 based on a clinically meaningful decrease [35,36], a pooled SD of 2, an α of .05, and a power of 80% based on the pilot study results [34]. For physical activity, we assumed a noninferiority margin of 12, a pooled SD of 35, an α of .05, and a power of 80%. We required 106 participants per arm. We did not expect the dropout rate for WIC2 to exceed 7%; thus, we aimed to enroll and randomize 228 and retain 212 participants.

### Statistical Analysis

Sociodemographic characteristics were compared by arm using chi-square and Fisher exact tests as appropriate for categorical variables and 2 sample *t* tests or Wilcoxon rank sum tests for continuous variables. Within-group changes from baseline to follow-up on mean HbA_1c_ and mean physical activity between the VW and IP study arms were assessed with paired *t* tests; between-group changes were assessed with multiple linear regression models, both at an α level of .025 after applying a Bonferroni correction for multiple testing. Between-group differences in the likelihood of achieving a 0.4% reduction or more in HbA_1c_ at follow-up were examined by logistic regression. One-sided *P* values and 97.5% CIs were calculated to assess the noninferiority hypothesis, using margins of 0.7% for HbA_1c_ and 12 MET-hours for physical activity. Other statistical tests and confidence intervals were 2-sided. Per-protocol (PP) analyses were limited to participants who completed the protocol as intended, by attending ≥6 out of 8 DMGVs. PP and intention-to-treat (ITT) analyses were conducted on a full data set that used multiple imputation via predictive mean matching to impute missing baseline, 9-week, and 6-month primary and secondary outcomes. Analyses were replicated on unimputed data to check the sensitivity of results to imputation.

Accelerometry data was used to calculate participants’ mean change in physical activity behavior from baseline to 6 months. For each participant, we randomly selected the 2 weekdays with the longest wear-time. We considered missing wear time data in a 24-hour day as sedentary activity. For each weekday, we estimated total MET-hours by a weighted sum of the number of hours in light (1.5 MET), moderate (4 MET), vigorous (6 MET), and very vigorous (8 MET) activity as measured by the accelerometer using the Freedson et al cut points [37,38]. We then averaged the estimated MET-hours for the 2 weekdays with the longest wear-time to obtain weekday average MET-hours per participant.

Sensitivity analyses were performed to evaluate the influence of language preference on our primary outcome results. Participant characteristics with, versus without, baseline HbA_1c_ were assessed to detect potential bias from missing data. Characteristics of participants who adhered to the session protocol (attended ≥6 vs <6 sessions) were also assessed, and primary outcome PP analyses were replicated controlling for characteristics found to be correlated with protocol adherence. All analyses were performed using SAS/STAT software (SAS version 9.4; SAS Institute) or the R programming language (version 3.4.3; R Core Team).

### Ethics Approval

This study was conducted according to the CONSORT (Consolidated Standards of Reporting Trials) guidelines [39] and approved by the Boston University/Boston Medical Center Institutional Review Board (H-34220).

## Results

### Study Population

**Figure 2:**
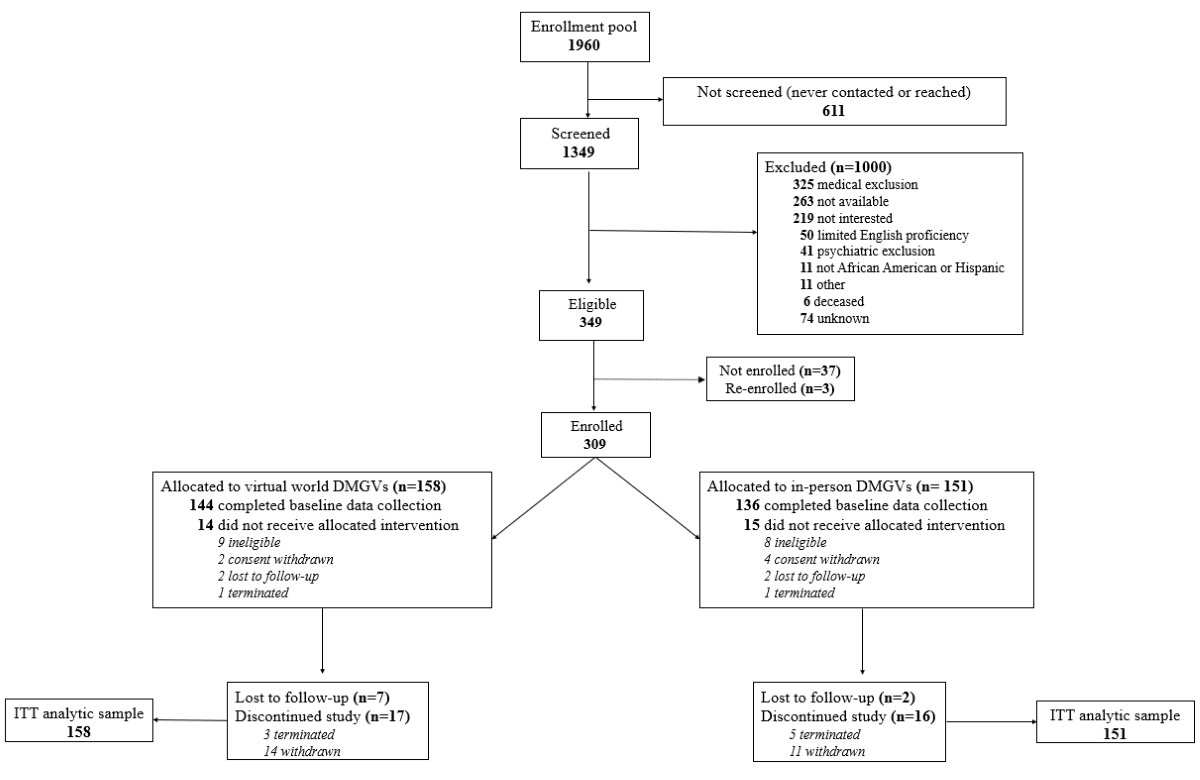
Flowchart of Women in Control 2.0 (WIC2) participants. DMGV: diabetes medical group visit; ITT: intention-to-treat.

Of 1960 potentially eligible patients, 1349 were screened, and 309 participants were randomized; 29 patients had a change in eligibility status before the first DMGV (Figure 2). The PP sample included 207 (108 VW and 99 IP; 67%) participants who met *a priori* criteria by attending ≥6 DMGVs. At baseline, participants’ mean age was 55 (SD 10.6) years, their mean weight was 195 (SD 41.8) lb, and their mean physical activity was 104.1 (SD 34.3) MET-hours. All participants were female, with 63% (195/309) of African American or Black race and 34% (105/309) Hispanic or Latina ethnicity (Table 1). A majority (219/309, 71%) were insured by Medicaid, Medicare, or both, and 59.6% (184/309) had home internet. The mean DD score was 2.3 (SD 1.0), which is moderately high, and the PHQ-8 score for depressive symptoms was 5.5 (SD 5.0), which is mild. More IP participants owned a smartphone. Mean HbA_1c_ values differed by VW and IP groups (mean 9.7%, SD 1.7% vs mean 10.2%, SD 1.8%), respectively. Participant characteristics with complete versus missing baseline HbA_1c_ data and changes in eligibility status were compared (Tables S1 and S2 in Multimedia Appendix 2 [27-31]). Because no participant characteristic was identified as accountable for the imbalance in mean baseline HbA_1c_, we attributed the difference in baseline HbA_1c_ to random imbalance and controlled for baseline HbA_1c_ in our outcome analyses.

**Table 1:** Baseline characteristics of Women in Control 2.0 (WIC2) study participants.

Characteristic		Overall (n=309)^a^	In-person (n=151)	Virtual world (n=158)
**Age (years)**				
	Mean (SD)	55.4 (10.6)	55.20 (9.6)	55.54 (11.5)
	Participants, n	300	146	154
**Race, n (%)**				
	African American or Black	195 (63.1)	96 (63.6)	99 (62.7)
	White	26 (8.4)	13 (8.6)	13 (8.2)
	Missing	10 (3.2)	5 (3.3)	5 (3.2)
	Other^b^	78 (25.2)	37 (24.5)	41 (25.9)
Hispanic, Latina, or Spanish ethnicity, n (%)		105 (34.0)	49 (32.5)	56 (35.4)
Spanish-speaking, n (%)		73 (23.6)	35 (23.2)	38 (24.1)
**Insurance, n (%)**				
	Commercial	69 (22.3)	31 (20.5)	38 (24.1)
	Medicare or Medicaid	219 (70.9)	109 (72.2)	110 (69.6)
	No insurance	4 (1.3)	2 (1.3)	2 (1.3)
**Education, n (%)**				
	High school graduate or less	152 (49.2)	74 (49.0)	78 (49.4)
	Any college, vocational, or trade school	132 (42.7)	65 (43.1)	67 (42.4)
	Any postgraduate	14 (4.5)	6 (4.0)	8 (5.1)
	Missing	11 (3.6)	6 (4.0)	5 (3.2)
**Employment status, n (%)**				
	Full-time	75 (24.3)	36 (23.8)	39 (24.7)
	Part-time	44 (14.2)	24 (15.9)	20 (12.7)
	Not employed	156 (50.5)	75 (49.7)	81 (51.3)
	Other	12 (3.9)	6 (4.0)	6 (3.8)
	Missing	22 (7.1)	10 (6.6)	12 (7.6)
**Financial insecurity, n (%)**				
	Does not have enough money to make ends meet	161 (52.1)	77 (51.0)	84 (53.2)
**Annual income, n (%)**				
	≤US $29,999	166 (53.7)	87 (57.6)	79 (50.0)
	≥US $30,000	59 (19.1)	24 (15.9)	35 (22.2)
	Refused to answer or don’t know	75 (24.3)	35 (23.2)	40 (25.3)
	Missing	9 (2.9)	5 (3.3)	4 (2.5)
**Marital status, n (%)**				
	Married	74 (24.0)	34 (22.5)	40 (25.3)
	Single with partner	34 (11.0)	17 (11.3)	17 (11.8)
	Single for any reason	192 (62.1)	95 (62.9)	97 (61.4)
	Missing	9 (2.9)	5 (3.3)	4 (2.5)
Health care head of household, n (%)		118 (38.2)	57 (37.8)	61 (38.6)
Has internet access, n (%)		184 (59.6)	90 (59.6)	94 (59.5)
Has smartphone, n (%)		237 (76.7)	125 (82.8)	112 (70.9)
Low health literacy, n (%)		87 (28.2)	44 (29.1)	43 (27.2)
**BMI (kg/m^2^)**				
	Mean (SD)	34.12 (6.9)	33.95 (6.7)	34.29 (7.2)
	Participants, n	285	138	147
**HbA_1c_^c^, %**				
	Mean (SD)	9.93 (1.7)	10.16 (1.7)	9.70 (1.7)
	≥9.0, n (%)	181 (58.6)	95 (62.9)	86 (54.4)
	Participants, n	284	138	146
**PHQ-8 score^d^**				
	Mean (SD)	5.49 (5.0)	5.10 (4.8)	5.86 (5.2)
	Participants, n	299	145	154
**Diabetes distress score^e^**				
	Mean (SD)	2.69 (1.4)	2.65 (1.5)	2.72 (1.4)
	Participants, n	298	145	153

^a^Percentages are based on the n value of each column (column %).

^b^Other category: 66 participants reported “some other race”; 8 reported “multiple races”; 1 reported Native Hawaiian, Pacific Islander; and 3 reported American Indian or Alaska Native.

^c^HbA_1c:_ hemoglobin A_1c_.

^d^Assessed using the Patient Health Questionnaire-8 (PHQ-8), which ranges from 1 to 8 [29].

^e^Assessed using the Diabetes Distress Scale- 17 [27,28].

### Fidelity

The 17 study cohorts were conducted with 98% fidelity to the 8-week curriculum. The median number of sessions attended by participants was 6 in the IP arm and 7 in the VW arm. Among participants who attended WIC2 DMGVs, 98.2% (1618/1648 total events) completed the clinician consult and intake forms. In the VW condition, participants completed the clinical consult via telehealth (540/823, 65.6%), telephone (54/823, 6.6%), or either modality (230/823, 27.9%).

### Coprimary Outcomes—ITT

Changes in HbA_1c_ and physical activity are reported in Tables 2 and 3. In the ITT sample, we found within-group HbA_1c_ improvements of 0.8% among IP participants, from 10.2% at baseline to 9.4% at 6 months, and 0.5% among VW participants, from 9.7% at baseline to 9.2% at 6 months. Improvements in HbA_1c_ values were not statistically significant between groups and were noninferior (the mean difference across study arms was 0.3 (97.5% CI –∞ to 0.3); *P*<.001). The upper limit did not cross the predetermined noninferiority margin of 0.7%. It would require a noninferiority margin of less than 0.3% to fail to reject the null hypothesis of inferiority.

**Table 2:** Changes in HbA_1c_ among Women in Control 2.0 (WIC2) participants.

Analysis type and study arm		HbA_1c_^a^, % Baseline to 6 months				HbA_1c_ noninferiority (margin of 0.7)			
		Baseline	6 months	Change	Mean difference (1-sided 97.5% CI)			*P* value	
**PP^b^ (n=207)**							0.2 (–∞ to 0.3)		<.001
	IP^c^, mean (SD)	10.1 (1.8)	9.4 (2.1)	–0.7 (1.8)					
	IP, mmol/mol	87	79	N/A^d^					
	VW^e^, mean (SD)	9.6 (1.7)	9.1 (1.8)	–0.5 (1.6)					
	VW, mmol/mol	81	76	N/A					
**ITT^f^ (n=309)**							0.3 (–∞ to 0.3)		<.001
	IP, mean (SD)	10.2 (1.8)	9.4 (2.2)	–0.8 (1.9)					
	IP, mmol/mol	88	79	N/A					
	VW, mean (SD)	9.7 (1.7)	9.2 (2.1)	–0.5 (1.8)					
	VW, mmol/mol	83	77	N/A					

^a^HbA_1c_: hemoglobin A_1c._

^b^PP: per-protocol.

^c^IP: in-person.

^d^N/A: not applicable.

^e^VW: virtual world.

^f^ITT: intention-to-treat.

**Table 3:** Changes in physical activity among Women in Control 2.0 (WIC2) participants.

Analysis type and study arm		Work week MET^a^-hours Baseline to 6 months				Work week MET-hours noninferiority (margin of 12)			
		Baseline	6 months	Change	Mean difference (one-sided 97.5% CI)			*P* value	
**PP^b^ (n=207)**							–3.0 (–8.9 to ∞)		.008
	IP^c^, mean (SD)	105.3 (35.3)	101.4 (34.9)	–5.2 (37.7)					
	VW^d^, mean (SD)	106.6 (35.4)	100.6 (38.4)	–8.1 (37.7)					
**ITT^e^ (n=309)**							–3.1 (–6.9 to ∞)		.02
	IP, mean (SD)	105.5 (40.1)	106.3 (45.4)	–6.5 (43.6)					
	VW, mean (SD)	106.4 (37.1)	105.1 (46.9)	–9.6 (44.8)					

^a^MET: metabolic equivalent of task.

^b^PP: per-protocol.

^c^IP: in-person.

^d^VW: virtual world.

^e^ITT: intention-to-treat.

For physical activity, IP and VW participants had mean within-group decreases of 6.5 MET-hours and 9.6 MET-hours, respectively. Between-group differences were not detected from baseline to post intervention. Still, the noninferiority of the VW approach was confirmed (the mean difference across arms was –3.1 MET-hours, 97.5% CI –6.9 to ∞; *P*=.02). It would require a noninferiority margin of less than 9 MET-hours to fail to reject the null hypothesis of inferiority.

### PP Results

Among the 207 participants who attended at least 6 DMGVs, within-group mean HbA_1c_ values improved by 0.7% among IP participants, from 10.1% at baseline to 9.4% at 6 months, and by 0.5% among VW participants, from 9.6% at baseline to 9.1% at 6 months. Noninferiority was confirmed with a mean difference of 0.2 (97.5% CI –∞ to 0.3; *P*<.001) in HbA_1c_ across study arms. Improvements of ≥0.4% were achieved by 56% (56/99) of IP and 52% (56/108) of VW participants from baseline to 6 months, while nearly one-third (75/207, 36.02%) achieved a 1% improvement. IP and VW participants’ physical activity decreased, on average, by 5.2 MET-hours and 8.1 MET-hours, respectively. Noninferiority was confirmed in the PP sample (the mean difference across arms was –3.0, 97.5% CI –8.9 to ∞; *P*=.008). Similar to the ITT analyses, between-group changes in HbA_1c_ and accelerometer-measured physical activity were not statistically significant.

### Secondary Outcomes

We analyzed mean changes in HbA_1c_ and physical activity data at 9 weeks from baseline, which were similar to the 6-month results (Table S3 in Multimedia Appendix 2). We compared the mean change in DD, depression symptom burden, physical functioning, patient activation, weight, and step count by study arm, and adjusted for the baseline values. We observed substantial within-group improvements in both study arms for total DD and some DD subscales (emotional burden, regimen, and interpersonal). We observed substantive but nonsignificant improvements in both study arms for depression symptom burden, physical functioning, and patient activation, and mixed results for weight (Table 4). Notably, the total participants reporting moderate DD (scores of ≥2) decreased from 53% (156/294) to 33% (77/237), and the proportion with clinically meaningful depressive symptoms (scores of ≥5) decreased from 48% (142/295) to 40% (95/237) from baseline to 6 months (Table S4 in Multimedia Appendix 2). Physical activity assessment using step count revealed high baseline step counts, small decreases at 6 months, and overall null findings (Table 4). Results of sensitivity and unimputed analyses are in Table S5-S10 in Multimedia Appendix 2.

**Table 4:** Group changes in secondary outcomes among Women in Control 2.0 (WIC2) study participants.

				Baseline, mean (SD)		Changes from baseline to 9 weeks					Changes from baseline to 6 months				
						LS^a^, mean (SE)		Between-group *P* value			LS, mean (SE)			Between-group *P* value	
**Total DD^b^**															
	**PP^c^**								.14						.33
		IP^d^	2.3 (1.0)		–0.5 (0.1)					–0.3 (0.1)					
		VW^e^	2.3 (1.0)		–0.3 (0.1)					–0.2 (0.1)					
	**ITT^f^**								.35						.69
		IP	2.2 (1.0)		–0.4 (0.1)					–0.2 (0.1)					
		VW	2.3 (1.1)		–0.2 (10.1)					–0.2 (0.1)					
**DD: emotional burden**															
	**PP**								.01						.28
		IP	2.7 (1.5)		–0.7 (0.2)					–0.3 (0.1)					
		VW	2.7 (1.5)		–0.4 (0.2)					–0.2 (0.1)					
	**ITT**								.26						.68
		IP	2.6 (1.5)		–0.6 (0.2)					–0.2 (0.1)					
		VW	2.7 (1.4)		–0.3 (0.2)					–0.2 (0.1)					
**DD: physician**															
	**PP**								.27						.31
		IP	1.5 (0.9)		–0.2 (0.1)					–0.1 (0.1)					
		VW	1.5 (1.0)		0.0 (0.1)					–0.1 (0.1)					
	**ITT**								.53						.98
		IP	1.5 (0.9)		–0.1 (0.1)					0.0 (0.1)					
		VW	1.6 (1.1)		0.0 (0.1)					–0.1 (0.1)					
**DD: regimen**															
	**PP**								.08						.76
		IP	2.7 (1.3)		–0.7 (0.2)					–0.3 (0.1)					
		VW	2.7 (1.4)		–0.4 (0.2)					–0.3 (0.1)					
	**ITT**								.34						.56
		IP	2.6 (1.3)		–0.5 (0.2)					–0.2 (0.1)					
		VW	2.7 (1.3)		–0.3 (0.2)					–0.2 (0.1)					
**DD: interpersonal**															
	**PP**								.77						.62
		IP	2.0 (1.3)		–0.3 (0.2)					–0.2 (0.1)					
		VW	2.0 (1.3)		–0.3 (0.2)					–0.2 (0.1)					
	**ITT**								.98						.98
		IP	1.9 (1.3)		–0.2 (0.1)					–0.1 (0.1)					
		VW	2.0 (1.3)		–0.2 (0.2)					–0.1 (0.1)					
**PHQ-8^g^**															
	**PP**								.66						.12
		IP	4.9 (4.8)		–0.7 (0.7)					–0.5 (0.3)					
		VW	5.5 (5.1)		–1.1 (0.7)					–0.3 (0.4)					
	**ITT**								.86						.99
		IP	5.1 (4.8)		–0.4 (0.6)					–0.2 (0.3)					
		VW	5.9 (5.3)		–0.7 (0.6)					–0.3 (0.3)					
**Physical functioning^h^**															
	**PP**								.42						.30
		IP	45.8 (9.8)		2.3 (1.3)					1.0 (0.7)					
		VW	46.5 (9.0)		1.0 (1.2)					0.3 (0.6)					
	**ITT**								.53						.36
		IP	45.8 (9.2)		2.6 (1.2)					0.9 (0.6)					
		VW	46.1 (8.9)		1.1 (1.2)					0.7 (0.5)					
**Patient activation^i^**															
	**PP**								.02						.63
		IP	67.5 (20.4)		7.7 (2.8)					1.6 (1.4)					
		VW	68.8 (17.4)		0.1 (2.5)					0.7 (1.2)					
	**ITT**								.12						.36
		IP	66.5 (21.1)		5.1 (2.5)					1.1 (1.2)					
		VW	66.0 (20.3)		0.4 (2.3)					1.1 (1.2)					
**Weight (lb)**															
	**PP**								.02						.91
		IP	193.2 (38.7)		–0.6 (5.6)					–0.6 (2.8)					
		VW	199.7 (45.6)		0.8 (6.1)					–0.6 (3.1)					
	**ITT**								.30						.83
		IP	194.0 (40.5)		–0.2 (5.0)					–0.6 (2.4)					
		VW	196.2 (44.7)		3.1 (5.1)					1.13 (2.9)					
**Step count**															
	**PP**								.01						.24
		IP	9779.4 (3187.1)		397.0 (483.2)					–219.8 (217.6)					
		VW	10,008.1 (2977.4)		–476.7 (417.1)					–338.2 (202.4)					
	**ITT**								.03						.35
		IP	9631.4 (3304.2)		297.2 (424.4)					–150.9 (198.6)					
		VW	9798.9 (3060.2)		–317.9 (378.7)					–168.3 (175.0)					

^a^LS: least squares.

^b^Assessed using the Diabetes Distress [DD] Scale-17, which ranges from 1 to 6 [27,28].

^c^PP: per-protocol.

^d^IP: in-person.

^e^VW: virtual world.

^f^ITT: intention-to-treat.

^g^Assessed using the Patient Health Questionnaire-8 (PHQ-8), which ranges from 1 to 8 [29].

^h^Assessed using the physical function subscale on the Patient-Reported Outcomes Measurement Information System (PROMIS-29) measure [30].

^i^Assessed using the Patient Activation Measure (PAM)-13 [31].

### Lifestyle Behaviors

Self-management behaviors were assessed through a weekly self-report to detect changes in diet, exercise, and diabetes-related medication. Nearly a third of all participants (89/309, 28.8%) reported ≥1 dietary change, with a greater proportion in the VW group compared to the IP group (55/158, 34.8% vs 34/151, 22.5%).

Of all participants, 65.7% (203/309) engaged in at least 20 minutes of exercise weekly during the intervention (VW: 106/158, 67.1% vs IP: 97/151, 64.2%). Only 17.2% (53/309) reported any type of change (increase, decrease, or switch) in their diabetes medication regimen (VW: 29/158, 18.4% vs IP: 24/151, 15.9%).

### Adverse Events

One study-related severe adverse event in the VW group occurred due to emotional distress.

## Discussion

### Principal Findings

To our knowledge, this is the first fully powered clinical trial to demonstrate the effectiveness of delivering DMGVs using an immersive 3D telemedicine platform versus IP care. Both approaches were similarly effective in reducing mean HbA_1c_ over 6 months. Our PP sample is indicative of a high patient retention rate. No significant changes in physical activity were detected. Altogether, this research demonstrates that 3D immersive telemedicine DMGVs are an effective alternative to IP group diabetes care for high-risk patients in a safety net health system.

Our preliminary study compared IP versus immersive DSME delivery among 89 low-income African American women with uncontrolled T2DM [34]. Results showed substantial improvements in mean HbA_1c_. Other pilot studies demonstrated positive impacts on patient outcomes but faced methodological limitations, including lack of a comparison group, small sample size, and inadequate power, and none used a DMGV format [40,41]. In contrast, the WIC2 study was randomized with an active DMGV control condition and fully powered to rigorously test the primary outcomes.

Nearly half of our participants at baseline had measurable depressive symptoms and diabetes distress. Prior research has revealed a strong correlation between depression, diabetes distress, and uncontrolled diabetes [42,43]. Interestingly, we found that the proportion of WIC2 participants with depressive symptoms (PHQ-8≥5) and diabetes distress (DD≥2) decreased from baseline to 6-month follow-up, indicating the WIC2 intervention improves glucose control and mental health. This finding is important as interventions that address both physical and mental health can reduce patients’ treatment burden.

Given our pilot study showed increased physical activity among study participants, the null finding in physical activity in WIC2 was unexpected [34]. We experienced challenges with accelerometry wear due to participants’ discomfort with the devices. A literature review revealed that accelerometry-measured activity for middle-aged women with chronic disease has limitations [44], such that physical activity can be underestimated or inconsistent across the life span [37,45,46]. More research is needed to establish activity assessment guidelines for older adults.

### Limitations

We acknowledge several study limitations. We had a small imbalance in HbA_1c_ at baseline. After careful assessment of participant characteristics, it was determined that this imbalance occurred at random and was unrelated to the fidelity of the study protocol. It is not possible to rule out unobserved confounding of protocol adherence, such as participants’ access to transportation, digital literacy, work or childcare conflicts, or financial constraints impacting access to medication. Finally, this study was conducted with women in an urban safety net health system, which may limit its generalizability. The ongoing challenges with access to digital resources and digital literacy for underserved communities may also limit the immediate generalizability of our study findings to similar populations.

### Conclusions

Immersive technologies can reduce disparities by improving effectiveness and access to evidence-based diabetes care. We showed that when given the tools, adults from digitally underserved communities robustly adopt health technology tools with improved health outcomes. More effort is warranted to design technology tailored to the needs, capabilities, and life perspectives of diverse communities to avoid leaving behind those most in need of better health care.

## Appendix

Multimedia Appendix 1 Women in Control 2.0 (WIC2) trial protocol.

Multimedia Appendix 2 Additional statistical analyses.

Multimedia Appendix 3 CONSORT eHEALTH checklist (V 1.6.1).

## Abbreviations

CONSORT: Consolidated Standards of Reporting Trials
DD: diabetes distress
DMGV: diabetes medical group visit
DSME: diabetes self-management education
HbA_1c_: hemoglobin A_1c_
IP: in-person
ITT: intention-to-treat
MET: metabolic equivalent of task
PHQ-8: Patient Health Questionnaire-8
PP: per-protocol
T2DM: type 2 diabetes mellitus
VW: virtual world
WIC2: Women in Control 2.0

## Data Availability

The WIC2 team agrees to share deidentified individual participant data that underlie the results reported in this study, the study protocol, and the statistical analysis plan. Data will be available for 6 months following publication for 5 years. Data will only be shared with academic researchers who provide a methodologically sound proposal to achieve aims related to primary or secondary outcomes and upon completion of a data use agreement. Requests should be directed to Suzanne.Mitchell2@umassmed.edu.

## References

[1] Peyrot M, Egede LE, Campos C, Cannon AJ, Funnell MM, Hsu WC, Ruggiero L, Siminerio LM, Stuckey HL (2014). Ethnic differences in psychological outcomes among people with diabetes: USA results from the second diabetes attitudes, wishes, and needs (DAWN2) study. Curr Med Res Opin.

[2] Chow EA, Foster H, Gonzalez V, McIver L (2012). The disparate impact of diabetes on racial/ethnic minority populations. Clin Diabetes.

[3] Marquez I, Calman N, Crump C (2019). A framework for addressing diabetes-related disparities in US Latino populations. J Community Health.

[4] Peek ME, Cargill A, Huang ES (2007). Diabetes health disparities: a systematic review of health care interventions. Med Care Res Rev.

[5] Walker RJ, Strom Williams J, Egede LE (2016). Influence of race, ethnicity and social determinants of health on diabetes outcomes. Am J Med Sci.

[6] Burke RE, Ferrara SA, Fuller AM, Kelderhouse JM, Ferrara LR (2011). The effectiveness of group medical visits on diabetes mellitus type 2 (dm2) specific outcomes in adults: a systematic review. JBI Libr of Syst Rev.

[7] Quiñones AR, Richardson J, Freeman M, Fu R, O'Neil ME, Motu'apuaka M, Kansagara D (2014). Educational group visits for the management of chronic health conditions: a systematic review. Patient Educ Couns.

[8] Edelman D, Gierisch JM, McDuffie JR, Oddone E, Williams JW (2015). Shared medical appointments for patients with diabetes mellitus: a systematic review. J Gen Intern Med.

[9] Cunningham SD, Sutherland RA, Yee CW, Thomas JL, Monin JK, Ickovics JR, Lewis JB (2021). Group medical care: a systematic review of health service performance. Int J Environ Res Public Health.

[10] Thompson-Lastad A (2018). Group medical visits as participatory care in community health centers. Qual Health Res.

[11] Adjei Boakye E, Varble A, Rojek R, Peavler O, Trainer AK, Osazuwa-Peters N, Hinyard L (2018). Sociodemographic factors associated with engagement in diabetes self-management education among people with diabetes in the United States. Public Health Rep.

[12] Rutledge SA, Masalovich S, Blacher RJ, Saunders MM (2017). Diabetes self-management education programs in nonmetropolitan counties - United States, 2016. MMWR Surveill Summ.

[13] Shaw K, Killeen M, Sullivan E, Bowman P (2011). Disparities in diabetes self-management education for uninsured and underinsured adults. Diabetes Educ.

[14] Rush KL, Hatt L, Janke R, Burton L, Ferrier M, Tetrault M (2018). The efficacy of telehealth delivered educational approaches for patients with chronic diseases: a systematic review. Patient Educ Couns.

[15] Mitchell SE, Mako M, Sadikova E, Barnes L, Stone A, Rosal MC, Wiecha J (2014). The comparative experiences of women in control: diabetes self-management education in a virtual world. J Diabetes Sci Technol.

[16] Boulos MN, Hetherington L, Wheeler S (2007). Second life: an overview of the potential of 3-D virtual worlds in medical and health education. Health Info Libr J.

[17] Peterson M (2005). Learning interaction in an avatar-based virtual environment: a preliminary study. PacCALL J.

[18] Fox J, Bailenson JN (2009). Virtual self-modeling: the effects of vicarious reinforcement and identification on exercise behaviors. Media Psychol.

[19] Webb TL, Joseph J, Yardley L, Michie S (2010). Using the internet to promote health behavior change: a systematic review and meta-analysis of the impact of theoretical basis, use of behavior change techniques, and mode of delivery on efficacy. J Med Internet Res.

[20] Mitchell S, Gardiner PM, Weigel G, Rosal M (2016). Women in control: pioneering diabetes self-management medical group visits in the virtual world. J Clin Trials.

[21] Mitchell S, Bragg A, Moldovan I, Woods S, Melo K, Martin-Howard J, Gardiner P (2021). Stigma as a barrier to participant recruitment of minority populations in diabetes research: development of a community-centered recruitment approach. JMIR Diabetes.

[22] Power to prevent: A family lifestyle approach to diabetes prevention. National Diabetes Education Program.

[23] Sousa VD, Rojjanasrirat W (2011). Translation, adaptation and validation of instruments or scales for use in cross-cultural health care research: a clear and user-friendly guideline. J Eval Clin Pract.

[24] Maneesriwongul W, Dixon JK (2004). Instrument translation process: a methods review. J Adv Nurs.

[25] Koster R (2013). A Theory of Fun for Game Design.

[26] Garber AJ, Handelsman Y, Grunberger G, Einhorn D, Abrahamson MJ, Barzilay JI, Blonde L, Bush MA, DeFronzo RA, Garber JR, Garvey WT, Hirsch IB, Jellinger PS, McGill JB, Mechanick JI, Perreault L, Rosenblit PD, Samson S, Umpierrez GE (2020). Consensus statement by the American Association of Clinical Endocrinologists and American College of Endocrinology on the comprehensive type 2 diabetes management algorithm - 2020 executive summary. Endocr Pract.

[27] Fisher L, Hessler DM, Polonsky WH, Mullan J (2012). When is diabetes distress clinically meaningful?: establishing cut points for the diabetes distress scale. Diabetes Care.

[28] Polonsky WH, Fisher L, Earles J, Dudl RJ, Lees J, Mullan J, Jackson RA (2005). Assessing psychosocial distress in diabetes: development of the diabetes distress scale. Diabetes Care.

[29] Kroenke K, Strine TW, Spitzer RL, Williams JB, Berry JT, Mokdad AH (2009). The PHQ-8 as a measure of current depression in the general population. J Affect Disord.

[30] Craig BM, Reeve BB, Brown PM, Cella D, Hays RD, Lipscomb J, Simon Pickard A, Revicki DA (2014). US valuation of health outcomes measured using the PROMIS-29. Value Health.

[31] Hibbard JH, Mahoney ER, Stockard J, Tusler M (2005). Development and testing of a short form of the patient activation measure. Health Serv Res.

[32] Harris PA, Taylor R, Minor BL, Elliott V, Fernandez M, O'Neal L, McLeod L, Delacqua G, Delacqua F, Kirby J, Duda SN, REDCap Consortium (2019). The REDCap consortium: building an international community of software platform partners. J Biomed Inform.

[33] Harris PA, Taylor R, Thielke R, Payne J, Gonzalez N, Conde JG (2009). Research electronic data capture (REDCap)--a metadata-driven methodology and workflow process for providing translational research informatics support. J Biomed Inform.

[34] Rosal MC, Heyden R, Mejilla R, Capelson R, Chalmers KA, Rizzo DePaoli M, Veerappa C, Wiecha JM (2014). A virtual world versus face-to-face intervention format to promote diabetes self-management among African American women: a pilot randomized clinical trial. JMIR Res Protoc.

[35] Taveira TH, Friedmann PD, Cohen LB, Dooley AG, Khatana SA, Pirraglia PA, Wu WC (2010). Pharmacist-led group medical appointment model in type 2 diabetes. Diabetes Educ.

[36] Khatana SA, Taveira TH, Choudhary G, Eaton CB, Wu WC (2009). Change in hemoglobin A(1c) and C-reactive protein levels in patients with diabetes mellitus. J Cardiometab Syndr.

[37] Freedson PS, Melanson E, Sirard J (1998). Calibration of the computer science and applications, Inc. accelerometer. Med Sci Sports Exerc.

[38] Matthews CE, Freedson PS, Hebert JR, Stanek EJ, Merriam PA, Ockene IS (2000). Comparing physical activity assessment methods in the seasonal variation of blood cholesterol study. Med Sci Sports Exerc.

[39] Schulz KF, Altman DG, Moher D, CONSORT Group (2010). CONSORT 2010 statement: updated guidelines for reporting parallel group randomised trials. BMC Med.

[40] Johnson C, Feinglos M, Pereira K, Hassell N, Blascovich J, Nicollerat J, Beresford HF, Levy J, Vorderstrasse A (2014). Feasibility and preliminary effects of a virtual environment for adults with type 2 diabetes: pilot study. JMIR Res Protoc.

[41] Ruggiero L, Moadsiri A, Quinn LT, Riley BB, Danielson KK, Monahan C, Bangs VA, Gerber BS (2014). Diabetes island: preliminary impact of a virtual world self-care educational intervention for African Americans with type 2 diabetes. JMIR Serious Games.

[42] Fisher L, Mullan JT, Arean P, Glasgow RE, Hessler D, Masharani U (2010). Diabetes distress but not clinical depression or depressive symptoms is associated with glycemic control in both cross-sectional and longitudinal analyses. Diabetes Care.

[43] Katon WJ, Von Korff M, Lin EH, Simon G, Ludman E, Russo J, Ciechanowski P, Walker E, Bush T (2004). The pathways study: a randomized trial of collaborative care in patients with diabetes and depression. Arch Gen Psychiatry.

[44] Moldovan IA, Bragg A, Nidhiry AS, De La Cruz BA, Mitchell SE (2022). The physical activity assessment of adults with type 2 diabetes using accelerometer-based cut points: scoping review. Interact J Med Res.

[45] Troiano RP, Berrigan D, Dodd KW, Mâsse LC, Tilert T, McDowell M (2008). Physical activity in the United States measured by accelerometer. Med Sci Sports Exerc.

[46] Miller NE, Strath SJ, Swartz AM, Cashin SE (2010). Estimating absolute and relative physical activity intensity across age via accelerometry in adults. J Aging Phys Act.

