# Supplementary material for: Effectiveness of an Immersive Telemedicine Platform for Delivering Diabetes Medical Group Visits for African American, Black and Hispanic, or Latina Women With Uncontrolled Diabetes: The Women in Control 2.0 Noninferiority Randomized Clinical Trial
Source: J Med Internet Res. 2023 May 10;25:e43669. doi: 10.2196/43669 (PMC10209787; doi:10.2196/43669)
Supplement: Multimedia Appendix 1 [file jmir_v25i1e43669_app1.pdf]

Study Application (Version 1.51)

1.0 General Information

***Please enter the official title of your study:**

Women in Control: A Virtual World Study of Diabetes Self-Management (Women in Control 2.0)

***Please enter the Study Nickname you would like to use to reference the study:**

Women in Control 2.0
* This field allows you to enter an abbreviated version of the Study Title to quickly identify this study.

2.0 Add Department(s)

2.1 List departments associated with this study (Note: The primary department should accurately reflect the primary Department or Section of the PI. Please verify that the primary department listed is correct (in some cases the "default" department of BU/BMC Medicine has been selected). For large departments, the PI's appropriate "section" should be listed as the primary department (e.g. if the PI is from Neurology or Infectious Disease). If the PI is from the SPH - select the appropriate department within SPH (e.g. Epidemiology) as primary.):

Primary Dept?	Department Name
○	**BMC/BUMC** - *BMC* - Endocrinology, Diabetes, Nutrition & Weight Management
●	**BMC/BUMC** - *MED* - Family Medicine
○	**BMC/BUMC** - *SPH* - Community Health Sciences
○	**BMC/BUMC** - *SPH* - School of Public Health

3.0 List of Internal (BMC/BUMC) Study Personnel. All personnel listed in this section will have access to this study (limited or full access).

3.1 * Please add a Principal Investigator for the study:
(Note: Only faculty members can serve as Principal Investigators on IRB protocols for studies at the School of Dental Medicine)

Mitchell, Suzanne, MD, MS

Select if applicable

☐ Student ☐ Resident

☐ Fellow

If the Principal Investigator is a Student, Resident, or Fellow, the name of the Supervising Principal Investigator (formerly known as Faculty Sponsor) must be supplied BOTH in Section 3.3 (Study Contact) AND in Section 3.4 (Supervising Principal Investigator) below.

3.2 If applicable, please select the Research Staff personnel. Individuals must be listed if they will have contact with research subjects or their identifiable data in the performance of any research related activities, including enrollment, consenting, collection of study data,

interventions, long-term follow-up or data analysis, either as Co-Investigators in A) or as Research Support Staff in B).

A) Additional Investigators

Gardiner, Paula, MD
Co-Investigator

B) Research Support Staff

Bragg, Alexa
Research Assistant
Data Analytics Center, Biostatistics
Biostatistician
De La Cruz, Barbara Amir, BA or BS
Research Assistant
Howard, Jessica, MA, MPH
Project Manager
Laird, Lance, Th.D.
Data Manager/Analyst

3.3 *Please add a Study Contact:

Bragg, Alexa
Howard, Jessica, MA, MPH
Mitchell, Suzanne, MD, MS

The Study Contact(s) will receive all important system notifications along with the Principal Investigator. The study contact(s) are typically either the Study Coordinator or the Principal Investigator. A Study Contact must also be listed in Section 3.1, 3.2, 3.4, or 3.6. If the PI is a student, resident, or fellow, the Supervising Principal Investigator MUST be entered here.

3.4 If the PI is a student, resident, or fellow, you MUST add the Supervising Principal Investigator here:

3.5 Please ONLY list the PI's Department Chair/Section Chief below. The system will automatically route for signoff to any additional "Special Routing" approvals, so please do not list those here.

Jack, Brian, MD
Department Chair/Section Chief

Add the name of the individual authorized to approve and sign off on this study from your Department (e.g. the Department Chair or Dean). This should be someone other than the Principal Investigator. For more information, **click here.

3.6 If applicable, please select the Administrative Assistant(s):

List here anyone performing administrative tasks only (not engaged in research and having no contact with subjects or identifiable data; where training and COI disclosure are not required) An Administrative Assistant can also be a Study Contact.

4.0 Review Path Determination

4.1 Review Path Determination

- ○ This project meets the definition of Not Human Subject Research (NHSR). Examples are non-research Quality Improvement/Quality Assurance projects; studies that involve obtaining anonymous data /tissues or coded data; or BMC/BU Medical Campus is not 'engaged' in human subjects research.
- ○ BMC/BU Medical Campus (the Relying Institution) cedes IRB review to another institution (the Reviewing Institution) under an Authorization Agreement.
- ○ The only research activities in this study involve chart reviews.
- ○ This study fits into one or more of the federal Exempt categories or the study does not have external funding and fits into one or more of the Equivalent Protections Exempt categories.
- ◉ None of the above. This study requires Expedited review or the review of the Full Board.

4.2 Emergency Use Report

Is this a report of an Emergency Use of an Investigational Drug or Device that has already occurred? For more information, click **here**.

○ Yes ◉ No

4.3 Individual Patient IND

Is this application for an FDA approved Individual patient (single use) IND under **21 CFR 312.310**?

○ Yes ◉ No

4.4 Humanitarian Use Device

Is this application for an FDA approved Humanitarian Use Device under **21 CFR 814**?

○ Yes ◉ No

5.0 Required Training and Conflict of Interest

5.1 BMC/BU Medical Campus Institutional Requirements for training

☑ The PI confirms the following:

- All individuals at Boston Medical Center or Boston University Medical Campus who will have contact with subjects or their identifiable data have been listed on this application in Section 3.0 (including those who will obtain informed consent, analyze identifiable data, perform study interventions, recruit subjects, etc.)
- All individuals listed in Section 3.0 have completed their INSPIR profile or have been asked to do so.
- All individuals listed in Sections 3.1, 3.2, and 3.4 are up to date with human subjects training and with GCP training if required. For more information, click **here**.

5.2 Conflict of Interest Disclosure

I confirm that **all** those responsible for the design, conduct, or reporting of the proposed program, including at minimum, all Senior/key personnel in the grant application, will, before this application is submitted, have completed the required financial interest disclosure through **COI Smart** for **Boston Medical Center** or through the **Financial Interest Disclosure form** for **Boston University**. NOTE: The IRB considers any missing financial interest disclosures to be noncompliance by the Principal Investigator.

☑ I confirm

Of the financial interest disclosure forms that will be completed, will any significant financial interests that are related to the research be disclosed?

○ Yes ◉ No

6.0 Funding Source

6.1 Funding Source

What is the source of your research funding? If you have multiple sources of funding (including sub-awards), check all that apply.

☐ Student/Resident/Fellow Research with no External Funding (choose if the PI is a student/resident/Fellow and the study is student/resident/Fellow research)
☐ Department/Internally Funded (choose if the PI is not a student/resident/Fellow and the study has no specific funding)
☑ Government
☐ Industry
☐ Foundation/Other
☐ Training Grant (e.g. T32, K-award)

6.2 Study Type

This study is:

◉ Initiated by the BMC/BU Medical Campus PI
○ Other

Does this study meet the definition of a clinical trial as defined by the International Committee of Medical Journal Editors? (See help for definition)

◉ Yes ○ No

NOTES:

- Studies that meet the ICMJE definition will **not** receive final IRB approval until the IRB is provided with the NCT number from **clinicaltrials.gov**. The responsibility for registering falls to the PI or Sponsor of the trial. For more information, click **here**.
- Clinical trials that are also initiated by the BMC/BU Medical Campus PI are **required** to consult with the **CRRO** prior to submission. Note that this pre-review consultation requirement is not satisfied by a consultation about registering with clinicaltrials.gov.
- All **BMC** and **BU Medical Campus** investigators or research team members that need assistance with **ClinicalTrials.gov** should contact ***Karla Damus*** *in the CRRO (**damusk@bu.edu**, (617) 358-7382)* . Examples of help include: registration of a clinical study to obtain the **NCT identifier** required by the IRB for all clinical trials, accessing/becoming a ClinicalTrials.gov user, resetting a forgotten password, and updating or reporting results for registered studies.

Date of pre-review consultation with the CRRO:

If this trial has been registered, please enter the 8 digit NCT number in the box, below:

02726425

6.3 Funding Details

For instructions on how to complete this section, click on the Help icon.

View Details	Sponsor Name	Sponsor Type	Contract Type:	BU SAP Grant Number or BMC AU Number	Award Number
⊟	NIH/National Institute of Diabetes and Digestive and Kidney Diseases (NIDDK)	Federal - NIH	Grant		1R01DK106531-01

Sponsor Name:	NIH/National Institute of Diabetes and Digestive and Kidney Diseases (NIDDK)
Sponsor Type:	Federal - NIH
Sponsor Role:	Payor;
**Grant/Contract Number:**	
**Project Period:**	From:08/01/2015 to:05/31/2020
**Is Institution the Primary Grant Holder:**	Yes
Contract Type:	Grant
BU SAP Grant Number or BMC AU Number:	
Award Number:	1R01DK106531-01
Grant Title:	
PI Name:<br>(If PI is not the same as identified on the study.)	

6.4 Grants Office

In the check boxes below, please indicate which grants office is handling your award/ sub-award.

☐ BU Office of Sponsored Programs (OSP-med)

☑ BMC Research Finance (RF)

☐ BMC Clinical Trial Office (CTO)

☐ Charles River Campus Office of Sponsored Programs (OSP-CRC)

☐ Other (must list below)

Funding Notifications:

☑ I have received a Notification of Award (NoA)
☐ I have received a Just In Time notice (JIT)
☐ I have received a fundable score for this study.

7.0 Study Summary

7.1 Provide a brief summary of the project in terms understandable to a non scientist (in 500 words or less). Do NOT copy from a grant application.

The prevalence of diabetes mellitus (DM) in the US is disproportionately high among minority women. Hispanic and Black/ African American women also report low rates of physical activity, low consumption of fruits and vegetables, and low leveles of diabetes self-efficacy, all significant risk factors for poor glycemic control and serious health complications. In order to participate as partners in healthcare, DM patients need self-management education and support provided in collaboration with teams of clinicians. Diabetes self-management (DSM) support is effective in helping DM patients make good choices and achieve clinical goals but is difficult to deliver in medical practice settings. Virtual reality technology can assist DM patients and their clinical teams with DSM support by providing effective educational tools in an engaging, learner-centered context that fosters self-efficacy and skill proficiency. Our prior work demonstrated that virtual worlds, like Second Life (SL), are suitable for supporting DSM education for patients. SL, an Internet-based virtual world, is an example of an immersive, three-dimensional environment which supports social networking and interaction with information. We now aim to enhance our curriculum using a medical group visit design to study whether the *Women in Control* virtual world group medical visit leads to similarly effective health and educational outcomes copared to face-to-face group medical visits. Our aims are to conduct a randomized, controlled trial of the comparative effectiveness of a virtual world DSM group medical visit format vs. a face-to-face DSM group visit format to increase physical activity and improve glucose control among Black/ African American and Hispanic women with uncontrolled DM at six month follow up, and to conduct a qualitative, ethnographic study of participant engagement with the virtual world platform during the virtual world group sessions, between group sessions, and following completion of the eight-week curriculum to characterize learners' self-directed interactions with the technology platform and assess the correlation of these interactions with the DSM behaviors and diabetes control.

8.0 Navigation Menu

Please note: Questions in the Navigation Menu section determine which subsequent sections will be displayed and which ones will be hidden. If later you make any change to the Navigation Menu section, you will need to click on the "Save and Continue to Next Section" button throughout the whole application to display any new required section or hide any sections that are no longer required.

8.1 Separate Protocol

Is this a new submission with a separate protocol? This protocol must be from the sponsor or cooperative group or be based on the **protocol template** found on the IRB website, and must include the purpose, inclusion/exclusion criteria, design/procedure, and data safety and monitoring plan. A separate protocol is REQUIRED for all initial submissions of medical or surgical clinical trials. Depending on the complexity of the study, the IRB Director or Chair may require a separate protocol for other types of initial submissions. Please contact **medirb@bu.edu** if you have questions about whether a separate protocol is needed.. A GRANT APPLICATION IS NOT A PROTOCOL.

- ○ Yes
- ◉ No
- ○ Not applicable, this is not a new submission

8.2 International Research

Are any BU/BMC investigators involved in any way in research activities at non-US sites, including oversight of international research activities?

○ Yes ◉ No

8.3 Subjects Recruitment

Is the PI/study staff recruiting subjects for this study?

- ◉ Yes
- ○ No

8.4 Subjects Consent

Will informed consent be obtained from any of the subjects?

- ◉ Yes
- ○ No

8.5 Genetics

Does this research involve genetic testing, gene therapy, or collection of genetic information?

○ Yes ◉ No

8.6 Biological Samples Collection

Does this study involve collecting biological samples for research purposes?

◉ Yes ○ No

8.7 Drugs/Biological Agents

Does this study involve administering drugs or biological agents?

○ Yes ◉ No

8.8 Devices

Does this study involve the use of one or more device (as **defined** by the FDA) other than for routine measurements or monitoring (e.g., an EKG machine)?

○ Yes ◉ No

8.9 Radiation

As part of this study, will subjects be exposed to any procedures involving ionizing radiation for research purposes only?

○ Yes
◉ No

8.10 Samples or Data Retained for Extra Use

Will you be collecting samples or data that will be retained for extra use by yourself or other investigators? Extra use means any analysis that is in addition to that required for the study endpoints. Please also answer Yes if this study has been submitted solely to establish a repository.

○ Yes ◉ No

8.11 StudyFinder Listing

Do you agree to have the study title, summary, and PI name and e-mail address listed on StudyFinder, a publicly viewable medical campus website for general publicity and collaboration purposes? (If you also want to use StudyFinder to recruit subjects, there is another question to answer in the Recruitment section.)

◉ Yes ○ No

9.0 Study Site Information

9.1 Select one:

◉ Single site research - conducted by BMC/BU Medical Campus investigator(s)
○ Multi-site research project - BMC/BU Medical Campus is a research site but is NOT the main study site
○ Multi-site research - BMC/BU Medical Campus is the main research site and/or the BMC/BU Medical Campus Principal Investigator is the overall PI of the entire study or the FDA sponsor

9.2 IRB Authorization Agreement – BMC/BU Medical Campus is the Reviewing Institution

Does this study have or require an Authorization Agreement for External (non-BMC/BU Medical Campus) investigators who will rely on BMC/BU Medical Campus IRB review? ***

◉ Yes ○ No

***If this study has or will require an IRB Authorization Agreement where BMC/BU Medical Campus investigators will rely on IRB review by another institution, do not check YES here, but instead, go to Section 4.1 and check the 2nd option, "BMC/BU Medical Campus (the Relying Institution) cedes IRB review to another institution (the Reviewing Institution) under an Authorization Agreement.".

10.0 IRB Authorization Agreement – BMC or BU Medical Campus is the Reviewing Institution

10.1 Identify the category which best describes the Relying Institution(s) and/or External Investigators. The BMC/BU Medical Campus IRB will be the IRB of Record (the Reviewing Institution) through an appropriate Authorization Agreement.

☑ B. External Investigators who fall into any of these Special Categories:

SPECIAL CATEGORIES

- BU-CRC faculty/staff (Institution: Enter "BU-CRC")
- BU-CRC students who are obtaining academic credit for their research activities (Institution: Enter "BU-CRC")
- Students from an outside institution (non-BU) who are obtaining academic credit for their research activities, including a Supervising Principal Investigator from their home institution (if required and listed in Section 9.3). (Institution: Enter name of institution)
- Physicians and staff from any of the Boston HealthNet Community Health Centers who will be engaged in research for the study (Institution: Enter name of Health Center)
- External Investigators who are working on the study independent of and/or not affiliated with any employer, institution, or organization (Institution: Enter "IIA")
- External Investigators who are affiliated with an institution that does not have an FWA (Institution: Enter "CIIA")
- External Investigators who are affiliated with an institution that has an FWA but is not a SMART IRB member. Only list the Site Principal Investigator.

10.2

TABLE OF EXTERNAL INVESTIGATORS

FOR OPTION B: List here all External Investigators (and their information) who fall into any of the "Special Categories" for Option B in 10.1 above. Enter the information in the Institution column as noted above in these "Special Categories".

Name	Institution	Telephone	E-mail	Role
Maria Pompeya Gomez	See IIA	9789856033	eddievgomez@yahoo.com	Project Staff
Jessica Baker	Boston University-CRC	6174146329	bakerj@bu.edu	Research Assistant
Sydney Georges	Boston University - CRC	617-414-6349	sgeorges@bu.edu	Research Assistant
Olivia Albenze	Boston University-CRC	617-414-6349	oalbenze@bu.edu	Research Assistant
Eddie Gomez	See IIA	626-298-0162	eddievgomez@yahoo.com	Research Assistant
Katherine Melo	Boston University - CRC	617-414-6349	katherine.melo@bmc.org	Research Assistant

Alexa Bragg	see IIA	2033797792	Alexa.Bragg@gordon.edu	Research Assistant
Dequindre Cummings	See IIA	617-905-7495	cdequindre@gmail.com	Technician

For each role that you have assigned to the external investigators listed above, explain what the role's tasks and duties will be on the study in the following table:

Role/Institution	Role Description
**Role:** Roles: Research Assistant<br>**Institution:** (Leave this as "None" if this role does not differ among institutions, or when the external investigator is not affiliated with any institution or organization)<br>IAA List: BU-Charles River Campus	BU-CRC Research Assistants will perform tasks such as data entry and verification, data collection, screening, enrolling, and consenting participants. All tasks will be performed under the supervision of the research program manager. CRC students added after the new 12/13/18 IRB policy regarding academic credit are indeed receiving academic credit for their work on the study.
**Role:** Roles: Technician<br>**Institution:** (Leave this as "None" if this role does not differ among institutions, or when the external investigator is not affiliated with any institution or organization)	Dequindre Cummings will be working with participants to assist them with technology. He will be providing troubleshooting support for iPhones, mifis, Second Life, and computers. He has completed Medical Campus CITI and GCP training.
**Role:** Roles: Research Assistant<br>**Institution:** (Leave this as "None" if this role does not differ among institutions, or when the external investigator is not affiliated with any institution or organization)	Eddie Gomez will be working as a research assistant to help facilitate the Spanish speaking Patient Advisory Groups and to assist with community outreach and community management of Spanish-speaking participants. He has completed Medical Campus CITI and GCP training.
**Role:** Roles: Intervention Facilitator<br>**Institution:** (Leave this as "None" if this role does not differ among institutions, or when the external investigator is not affiliated with any institution or organization)	Maria Pompeya Gomez will be serving in the capacity as a Peer Facilitator/ Ambassador during the group visits (Spanish only) portion of the research. Her role will be to encourage participation among the research participants, as well as to step in as a facilitator during various group activities. She will have access to identifiable data (participant's names), and she will be interacting with participants for the purpose of facilitating participation. Ms. Gomez may also attend enrollment day and community outreach events.

10.3 Conflicts of Interest

Do any of the External Investigators have a COI or potential COI? Please state Yes or No. If yes, please describe the nature of the COI and whether the Relying Institution has a COI program.

NO

10.4 **PI agreement to the terms of the IAA - PI must agree to these terms. I understand that, if this request is approved, the BMC/BU Medical Campus IRB ("the IRB") will be the IRB of record responsible for conducting the initial and continuing review of this protocol. I understand that the decision to cede IRB review is made jointly with the IRBs of the Relying Institution (or the Independent External investigator) and will not be the decision of the PI. The IRB, as the IRB of record, will have full responsibility for oversight of all aspects of the protocol EXCEPT for the following: The PI will have full responsibility for ensuring that the engaged research staff of the Relying Site Principal Investigator(s) in OPTION A have met all their home institutional requirements for ceded research. I will comply with the applicable policies of the IRB. I understand that this agreement is NOT considered approved until a formal Authorization Agreement is signed by the Institutional Officials of both institutions (or with each Independent External Investigator), and the fully signed Agreement is attached to this protocol. I understand that as PI for this study I am responsible the ethical conduct of this study. Oversight responsibilities include:**

- **Ensuring that all OPTION B External Investigators are listed in the Table of External Investigators of this application**
- **Ensuring that all the investigators follow the IRB protocol as approved and make no changes to the protocol without the approval of the IRB (except to eliminate immediate harm to subjects)**
- **Reporting to the IRB (per policy) any adverse events, protocol deviations, or unanticipated problems related to the research activities conducted by the External Investigators**
- **Reporting to the IRB any changes related to the status of the External Investigators**
- **Following all applicable HIPAA rules and using appropriate safeguards to prevent the unauthorized use or disclosure of PHI (Protected Health Information)**
- **Ensuring that External Investigators follow any determinations related to conflict of interest from BMC/BU Medical Campus or from their own Relying Institution.**

(•) Yes, as PI, I agree to the above terms.
( ) No, I do not agree to these terms (at which case BUMC will not agree to serve as the IRB of record for the External Investigators.)

11.0 Purpose

11.1 Background/Rationale/Purpose

Provide background information, study rationale, and purpose / study objective(s) and/or hypotheses for this study.

The prevalence of diabetes mellitus (DM) in the US is disproportionately high among minority women. Hispanic and Black/African American women also report low rates of physical activity, low consumption of fruits and vegetables, and low levels of diabetes self-efficacy, all significant risk factors for poor glycemic control and serious health complications. In order to participate as partners in healthcare and improve DM outcomes, patients need diabetes self-management (DSM) education and social support provided in collaboration with teams of clinicians. In practice, however, the actual delivery of this care model is fraught with challenges that limit the uptake of evidence-based care. With a shortage of healthcare providers and a growing prevalence of diabetes, there is great need for innovative approaches to DSM education and collaborative medical care to address disparities in DM outcomes through enhanced access and uptake of effective diabetes care.

Women in Control 2.0 represents an extension of our prior work, a pilot study, *Women in Control 1.0* , which tested a virtual world DSM group education intervention using the CDC's *Power to Prevent* curriculu m with excellent results. We now intend to expand the virtual world DSM group education program to include a medical consult consistent with the group medical visit model and a social ecological framework. While evidence shows the potential efficacy of Internet-based DSM education programs, to date, little work has focused on the use of virtual world technology for a group medical visit. Second Life, an Internet-based virtual world, is an example of an immerseive, three-dimensional environment which supports social networking, interaction with information, and skill simulations. The greater sense of "presence" in a virtual world affords opportunities for simulation experiences and influences peer group processes and cohesiveness more effectively than conventional eHealth applications.

Our prior work suggests that, in addition to expanding access to DSM education, virtual world technology provides tailored, experiential learning opportunities and impacts multiple levels of health behavior influences (individual, peers, healthcare organization and community). These attributes increase knowledge transfer and lead to higher uptake of key health behaviors compared with face-to-face, didactic classroom learning. We now aim to conduct a fully powered, randomized controlled trial to test the comparative effectiveness of delivering our innovative virtual world medical group visit intervention, *W omen in Control 2.0*, compared to a face-to-face medical group visit format. The intervention is an eight-week DSM program that uses the evidence-based Power to Prevent curriculum formatted as a medical group visit led by a healthcare clinician. If successful, this study will provide evidence demonstrating how the unique, untapped opportunities of virtual world platforms can enhance the delivery, access and uptake of evidencebased diabetes care for minority women with poorly controlled DM. We propose to test the following hypotheses with these specific aims:

Hypothesis 1: *The Women in Control DSM medical group visits delivered in a virtual world setting will be noninferior*
in increasing participant physical activity levels at six-month follow-up as DSM medical group visits conduc ted via face-to-face format among Black/African American and Hispanic women with uncontrolled DM.
Hypothesis 2: *The* Women in Control *DSM medical group visits delivered in a virtual world setting will be noninferior in improving HbA1c at six-month follow-up than DSM medical group visits conducted via face-to-face format among Black/African American and Hispanic women with uncontrolled DM.*

Specific Aim 1. To conduct a randomized controlled trial of the comparative effectiveness of a virtual world DSM group medical visit format vs. face-to-face DSM medical group visit format to increase physical activity at six-month follow-up among Black/African American and Hispanic women with uncontrolled DM.
Specific Aim 2. To conduct a randomized controlled trial of the comparative effectiveness of a virtual world DSM group medical visit format vs. face-to-face DSM medical group visit format to improve diabetes control (HbA1c) at six-month follow up among Black/African American and Hispanic women with uncontrolled DM.
Specific Aim 3. To conduct a qualitative, ethnographic study of participant engagement with the virtual world platform during DSM group medical visits in comparison to face-to-face visits, between group sessions, and following completion of the eight-week curriculum up to six months from baseline to characterize learners' selfdirected interactions with the technology platform and assess the correlation of these
interactions with real-world DSM behaviors.

Our study builds upon our prior work by engaging patients and clinicians in a novel virtual world DSM medical group visit. Using technology, we aim to offer a tailored, individualized simulation-based learning experience that links DSM education with patients' clinical care, daily lifestyle, peer support, home and virtual world community. If successful, this program would increase access to culturally-sensitive diabetes care and improve patient engagement in online DSM learning, leading to higher uptake of DSM behaviors and better diabetes control. Importantly, the program can be easily expanded to other chronic disease areas and scaled for widespread use.

12.0 Subjects

12.1 Inclusion Criteria

Include age ranges and sex. If study involves different criteria for different cohorts, please list separately.

Order Number	Criteria
1	Self-identifies as Black/African American and/or Hispanic
2	Self-identifies as female
3	Diagnosed with Type 2 Diabetes Mellitus
4	Last HbA1c level ≥ 8.0 (documented in medical chart) no more than 90 days prior to start of intervention
	On a clinician-supervised diabetes treatment plan (diet, oral hypoglycemic, insulin).

5	
6	18 years of age or older
7	Has telephone access
8	Can understand and participate in study protocol; able to understand and provide informed consent
8	Can communicate comfortably in English
9	Lives in permanent/stable housing
10	Patient seen at East Boston Neighborhood Health Center, if participating in a Spanish-speaking group

12.2 Exclusion Criteria

Include age ranges and sex. If study involves different criteria for different cohorts, please list separately. Do NOT duplicate inclusion criteria; if no additional criteria, indicate "None."

Order Number	Criteria
1	History of diabetic ketoacidosis
2	Currently pregnant or planning pregnancy
3	Unable or unwilling to provide informed consent
4	Plans to leave area during the 6 month study period for more than 2 weeks.
5	Requiredsystemic glucocorticoid therapy within past 3 months (i.e., prednisone, prednisolone, dexamethasone)
6	Experienced an acute coronary event (myocardial infarction) or heart valve condition like aortic stenosis) within the previous 6 months or has been diagnosed with a chronic heart condition (congestive heart failure, atrial fibriliation, unstable angina, abnormal heart rhythms, etc)
7	Medical condition that precludes adherence to study dietary recommendations (i.e., Crohn's disease, ulcerative colitis, end-stage renal disease)
8	Serious psychiatric illness (i.e., dementia, bipolar disorder, schizophrenia, paranoia) or psychiatric hospitalization in the last 6 months.
9	Experienced a stroke in the previous 6 months
10	Has been diagnosed with oxygen-dependent COPD or is oxygen-dependent for any other reason.
11	Active alcohol or illegal/prescription drug dependence or abuse
12	Is currently enrolled in a diabetes program at BMC (or elsewhere)
13	On dialysis
	Currently receiving treatment for cancer

14	

12.3 Race / Ethnicity

Will the expected demographic breakdown of the study population reflect either the Boston population or BMC population?

- (•) Yes
- ( ) No

12.4 Limited- and non-readers

NOTE: This question is new. If this submission is an amendment and your study is still consenting subjects, as a separate step, you must check the signature page of your active consent form(s). If any do not comply with the below requirements, you MUST submit edited consent form(s) meeting these requirements with this submission. See **Editing-Signature-Page** for more detailed instructions.

1. Limited- and non-readers excluded:

- No witness signature line AND
- Subject statement does not say “(or has been read to me)”

2. Limited and non-readers not excluded:

- Subject statement says “(or has been read to me)”
- If the study is greater than minimal risk, either a witness signature line appears or another method to assure and document subject comprehension is described in the Consent section

Are limited- and non-readers EXCLUDED from the study?

- ( ) Yes
- (•) No
- ( ) Not Applicable - No subjects are to be consented for this study (consent will be waived or enrollment is complete)

12.5 Special Populations (for more information, click on the (?) Help icon)

Please indicate if ANY (even one) of the following populations will be recruited (Note: Enrollment from any of these categories requires prior IRB approval):

- [ ] Minors who are wards of the State**
- [ ] Cognitively impaired subjects (will require use of an LAR, and assessment of ability to consent)**
- [ ] Employees, students, or trainees under the direct supervision of the PI**
- [ ] Minors**
- [ ] Minors independently making their own healthcare decisions**
- [x] Non-English speaking subjects**
- [ ] Pregnant Women**
- [ ] Prisoners**
- [ ] Women of child-bearing potential
- [ ] Individuals whose HIV testing status is provided to the study team prior to consent being obtained (e.g., for recruitment)
- [ ] Individuals identified as a patient of a federally-assisted substance use disorder clinic (Project RESPECT, Office-Based Addiction Clinic, CATALYST Clinic, or others - see (?) Help Icon for full list)

Please indicate if any of the following populations will be targeted by your research:

- [ ] BMC Residents or Fellows
- [ ] BU Dental Students

☐ BU Medical Students and/or Graduate Medical Sciences Students
☐ BU School of Public Health Students
☐ Homeless**
☐ Individuals with psychiatric disorders**
☐ Terminally ill patients**

**designated as vulnerable

12.6 You have selected one or more vulnerable population (indicated by ** above) that requires special protections. How will you protect their rights and welfare, obtain informed consent, and prevent undue influence and/or coercion? For Minors, Non-English speaking subjects and cognitively-impaired subjects, note that you will be asked additional questions in the Consent Procedures section which you may cross-reference here rather than repeating.

A portion of our study population will be Spanish-speaking women. We will translate all informed consent forms and curriculum materials into Spanish for these partcipants and all group visists will be conducted in Spanish for this cohort. Their confidentiality will be protected in the same ways as the English-speaking participants' confidentiality, and we will prevent undue influence and/or coercion by having all Spanish speaking women interact with Spanish-speaking research staff and clinicians as to avoid any language barriers and miscommunication.

13.0 Design/Procedure

13.1 Design and Procedure

Describe in detail the experimental design, including all materials and all procedures to be performed. Do NOT copy from a grant application – your application will be returned to you for revision if you do so.

Please include a clear timeline of the procedures to be performed. Clarify which procedures /test articles are investigational and which are part of standard clinical care. This description may include:

1. methods
2. specific information concerning experimental interventions, such as dose and frequency of drug (and placebo) administration, or deception/debriefing process for social behavioral studies
3. number, frequency and duration of subject contacts (visits, telephone calls, mail outs, emails)
4. entire duration of participation for a single subject
5. any additional requirements of the subject (post treatment follow-up, diary cards, questionnaires, etc.)
6. If any nursing staff (other than research nurses) are expected to interact with subjects, include a brief description of the plan for inservice training of nursing staff.

(Note: For multiple sites, indicate which of the procedures will be done at any other sites other than BMC/BU Medical Campus (see Study Site Information). Attach, in the Study Attachments section, copies of any surveys, questionnaires, and other data collection instruments.)

Patient Advisory Group:
Purpose & eligibility: Pre-RCT implementation, we will recruit a patient advisory group (PAG) to advise and provide feedback on the appropriateness/usefulness of the curriculum and the user-friendliness of the virtual world technology. The patient advisors will be adult women (18+ years) with diabetes or pre-diabetes who speak English. For the PAG, we will not exclude participants based on race/ethnicity, however will ensure that some patient advisors are bi-lingual (English and Spanish) and some are African American/Black, so they may be representative of the study population.

Screening and recruitment: We will recruit patient advisors by networking with BMC-affiliated staff known to care for diabetic patients, and who may be able to identify individuals who could serve as patient advisors. We will also make announcements at faculty meetings for the Department of Family Medicine. These doctors will then follow up with identified patients to explain the PAG and provide them with a flyer on the patient advisor role and contact information (See attached document). These flyers may also be posted by study staff around BMC. If the patient is interested, they will contact our research staff on the phone, who will ask them basic eligibility questions like gender, age and diabetes status (see the attached phone screening questionnaire). Interested participants will be included if they live in permanent, stable housing and/or do not live in a shelter program. If eligible, the research staff will schedule an in-person interview with the patient to further assess their suitability for the role. This interview will include open ended questions about the individual's interest in the study, and their availability to participate in the PAG (see attached contact and availability sheet). If the research staff believes the individual would be able to serve as a patient advisor, they will consent the individual into the study.

PAG responsibilities: Patient advisors will be asked to commit 4 hours per week to the PAG fora total of 10 weeks. Of the 10 weeks, 2 weeks will be for computer training and the remaining time for the 8-week group sessions; two of these hours per week will be attending in-person sessions at BMC in which they will be set up with a computer, internet and trained on the Second Life technology, progress through and review the 8-week DSM curriculum, and comment on the usability of the technology. They will also review the RCT enrollment form, to comment on its length and appropriateness of the outcome measures and surveys. The other 2-hrs per week, they will be asked to meet in Second Life, to further test the technology. After the initial 8 weeks, patient advisors will be asked to meet periodically as needed in Second Life (in the convenience of their own homes), to further comment on the iterative process of curriculum and technology development. The total length of commitment for the patient advisors will be up to 4 hours/week for 6 months.

Patient Advisory Group (SPANISH)

Prior to the start of our Spanish-Speaking groups in East Boston, we will recruit a Spanish-speaking participant advisory group (PAG) of 5-8 adult women who have been diagnosed with diabetes or pre-diabetes, who have telephone access, and who are available at the time of the PAG. The purpose of this PAG is to pilot our Spanish materials. This group will meet once or twice for 4 hours at a time at BMC to discuss the materials presented to them and to provide feedback about the Spanish curriculum to be implemented in East Boston. We will provide light refreshments during the PAG. PAG participants will be recruited from the pool of Spanish-speaking individuals originally recruited for English groups who preferred to be in Spanish groups. All of these individuals agreed to be recontacted when we first made contact with them.

RCT Implementation

Defining the Control Condition: Participants assigned to the control condition for this RCT will, like those assigned to the intervention group, participate in an 8-week DSM medical group visit program, consisting of 8-10 participants, however the context will be a face-to-face clinical classroom setting at the Boston Medical Center campus. Each of the sessions will include a 10-15 min individual clinical consult with a clinician. Didactic instruction in DSM will be presented by a study clinician and supported by a written manual tested in our pilot study and PAG for literacy level. The control curriculum content will be the same as the virtual world intervention and also based on the Power to Prevent DSM program. All materials will be culturally adapted and translated to Spanish for Spanish speaking participants. Thus, the primary difference between intervention and control arms will be the use of a virtual world platform as a learning environment compared to a traditional in-person classroom setting.

At the time of the 9-week follow-up visit, we will distribute paper trackers to control (and intervention) participants for use during 8 weeks of the "Maintenance" period following the 8 weeks of group visits. This tracker is intended to help participants engage in self-monitoring behaviors (i.e., checking blood glucsoe and blood pressure, assessing dietary and exercise patterns, etc.). No personal health information will be collected on the trackers, which will only be labeled with the participants' ID numbers. We will provide control (and intervention) participants with pre-addressed and stamped envelopes to return the paper trackers to the study team via mail at the end of the 8-week use period.

Defining the Intervention:
The intervention is characterized by specific attributes known to support successful health behavior change such as individually tailored information, peer support, access to healthcare advice and experiential learning to foster adoption of new health behaviors. The Women in Control 2.0 intervention involves:

- 8-wk series of interactive, virtual world medical group visit sessions
- Groups of 8-10 participants
- Weekly clinician & peer-led sessions based on Women in Control curriculum lasting ~180'' in length (up to 3 hours)

- Conducted either in Spanish or English
- Virtual individual consultation in Second Life with a clinician lasting 10-15 minutes
- Collaborative, experiential activities to reinforce learning and skill mastery
- Access to the peer community at any time
- All intervention participants will receive a computer training session that includes computer skills basics and Second Life Training. We will offer additional computer and Second Life training on an ad hoc/ as needed basis for individual participants. During the sessions, we will offer technical assistance to members of the Second Life group via telephone or via remote assistance technology.

The intervention has several distinct advantages from usual care. For example, a virtual world personally tailored experience involves the design of a unique avatar representation of self that then allows the individual to engage in self-directed activities with peers (such as selecting a meal from a menu in a virtual cafe). Ongoing access to peers engaged in a common health behavior experience for social and moral support addresses the frequent lapse in newly adopted health behaviors that occurs once formal group meetings end. The virtual medical group visit allows engagement with healthcare providers within the context of the individual's home community (like a virtual house call), linking the DSM intervention with clinical care. The virtual world group DSM education likewise links new DSM behaviors with one's daily life encouraging uptake of new lifestyle behaviors. Our virtual world curriculum will include DSM simulation experiences and constructionist educational activities such as visits to Club One, a SL health club, where participants engage in virtual physical activity, and other movement-based activities such as surfing, roller blading, dancing, etc.

Other activities might involve the prototyping of a community garden to grow healthy vegetables, working in the garden or designing a food pantry in the learner's virtual home dwelling to encourage collaborative learning, and critical thinking and agency toward healthy eating at home. Our prior work suggests these experiences lead to real world health behavior changes, such as increased physical activity, and improvements in diabetes control.

At the time of the 9-week follow-up visit, we will distribute a paper tracker to the intervention participants, as well as the control participants. No personal health information will be collected using the paper trackers. Participants will be asked to use the tracker for 8 weeks and to return it in a self-addressed, pre-stamped envelope provided to them.

Randomization: Once participants are recruited from participating community health centers and BMC primary care and endocrinology clinics and community partners, they will be randomly assigned either to intervention or control DSM group medical visit cohorts. There will be 12 cohorts of participants randomly assigned to a study arm either to participate in virtual world or face-to-face group medical visits.

After informed consent, we will randomize patients to either face-to-face or virtual diabetes group medical visits. Randomization will be at the level of the individual. Participants will not be assigned to group cohorts based on where they receive their care.We will use one-to-one block randomization (alternating blocks of 6 and 8) stratified for English or Spanish speakers. During recruitment, we will target one specific language group per cohort.

At least one cohort will be a Spanish-speaking cohort (n=24 participants per cohort, with groups of 12 participants assigned to either the control or intervention condition).

*Please note that the only retrospective aspect of this study is the prior assessment of HbA1c levels of participants, to confirm inclusion eligibility in this study.

Intervention and Study Design:
Curriculum Design:
The Women in Control curriculum content is based on the Power to Prevent (P2P) curriculum developed by the CDC for African Americans living with DM and the Mindfulness-Based Stress Reduction Curriculum and addresses the intrapersonal and interpersonal factors related to successful DSM. Topics covered in the 8-week series include: monitoring blood glucose and coping with symptoms, healthy food choices for those living with DM, fun and safe physical activity, overcoming mood challenges, talking with health professionals, shared decision making and involving family members in healthy lifestyles. The P2P curriculum is based in social cognitive theory and informed by the Diabetes Prevention Program, which demonstrated success in weight loss, reduction of HbA1c and DM incidence, reduced dietary fat and caloric intake, and increased physical activity relative to controls. The educational activities designed for the Women in Control virtual world DSM curriculum are based on the Positive Technological Development (PTD) pedagogical theory. PTD is an education technology pedagogical construct consistent with the social ecology model suggesting that learning with computers translates to multifaceted gains in knowledge, attitudes and skills. The emphasis is on designing constructivist learning activities (learning by building, creating and doing) within a larger social context (in collaboration with peers) to foster development of

personal assets that promote positive behaviors. Within this framework, the Women in Control curriculum targets intrapersonal characteristics (such as competence, confidence and character) and fosters interpersonal assets (such as caring, connection and contribution) to enhance participants' DSM skill mastery and to achieve a greater sense of personal agency and self-efficacy to sustain DSM behavior change over time. For the Women in Control virtual world intervention, the curriculum will be enhanced with experiential learning, simulation and collaborative constructivist activities to build DSM skill and mastery. For example, learners may visit a virtual world café to practice ordering healthy meals outside of home or be asked to collaboratively construct virtual world objects related to DSM support that contribute to the customization of the virtual world community for the peer group. Ambassadors for our subcontract partner, Virtual Ability Inc, will program supports ("point-and-click short cuts") to help users easily construct objects and design spaces within the Second Life environment. Clinicians will participate in formal instructional training to ensure all session leaders are familiar and knowledgeable of the curriculum content and skilled in facilitating group discussion and use of Second Life. To maintain a high degree of fidelity in the educational sessions, we will audio record all sessions and monitor randomly selected sessions for consistency in content and facilitator technique using a standardized checklist. Group leader remediation will be provided as needed.

Translation and Cultural Competency Enhancement to Curriculum for Hispanic Participants:
We will culturally adapt and translate the Women in Control curriculum for Spanish language instruction. We will engage a professional translation service in the Boston area to conduct the translation effort of our Women in Control curriculum booklet for use with Spanish language participants. Dr. Rosal, bilingual research staff, and peer leaders (TBD) will provide reviews of translated materials and field test the translated materials with members of the target participant community before materials are finalized. We will use whenever possible Spanish language patient education materials free of charge from the CDC website. To ensure that the Women in Control DSM curriculum is culturally competent for Hispanic participants, we will design culturally tailored adaptations of the Power-to-Prevent curriculum for Hispanic participant cohorts. Adaptations will include review and revision of dietary recommendations, modes of food preparation, recommendations for leisure activities, culturally sensitive strategies for overcoming mood challenges in diabetes self-management and addressing health beliefs about diabetes. Drs. Mitchell and Rosal are experienced in addressing unique concerns for Hispanic communities regarding diabetes care, in translation of patient education materials and the design of culturally tailored health information and will lead this effort.

Participant Involvement
The total duration of participation for each participant in this study is about 6 months, or fewer for those who might choose to withdraw earlier. The participant's main involvement will be over the course of 6 months; data collection will take place at baseline, during the 8-week session blocks, post-intervention and at 6 months follow up. Participants will spend about a 180 minutes per week, for 8 weeks in group DSM sessions, and 10-15 minutes per week for 8 weeks consulting with a clinician. Baseline, post-intervention and 6 month follow up data collection of all primary and secondary outcomes will take about 100 minutesm each.

Study clinicians meet with patients in both study arms each week during the group sessions for an individual consultation. The PCPs of these patients will only be flagged following a group session if the clinical consultation results in an actionable outcome (i.e. recommending medication or dosage change, continual high blood pressure, the study clinician recommending that a patient should be seen, an adverse event, etc.), to ensure that the PCP is aware of the outcome.

We will conduct up to 25 key informant interviews with select intervention and control participants during the 8-week group visit period, during the maintenance period, or after their study period has ended. These key informant interviews will focus on understanding the participant's experience with the DSM curriculum and Second Life, if applicable. Key informants will be paid $25 each.

We will choose a random sample of 50 praticipants from both the intervention and the control arms to participate in focus groups during the maintenance phase (following the 8 weeks of group visits) or after their study participation is complete. We will conduct up to 5 focus groups – 2 groups of control participants, 2 groups of intervention participants, and 1 group of participants from both study arms, during the maintenance phase (following the 8 weeks of group visits) or post-study period. These focus groups will focus on understanding the participant's experience with the DSM curriculum and/or Second Life. Individuals will be paid $25 for participation.

We will provide light refreshments during group sessions and data collection visits held at Boston Medical Center if the visit/ session is expected to last at least 2 hours.

Contingency Plans
In case of emergency or unexpected staffing problems (i.e. weather-related emergency, family emergency, etc) the clinician may hold the doctor visit outside of the schedule group time. If this were to occur, the individual clinician consults for both face-to-face and virtual world groups must take place within +/- 48 hours of the scheduled group. In addition, all consult notes must be documented in the

patient's EPIC medical record within 48 hours of the doctor visit. In case of an emergency, the research staff may also decide to cancel a group session if it is deemed unsafe (i.e. during winter blizzard), at the discretion of the PI and BMC updates. At this time the research team will decide if it is best to reschedule the missed group session during another time that week or if it is best to push back all groups by one week.

Participants who choose to leave the group visit (either the Second Life or the in-person group visit) without having had their clinician visit effectively "forfeit" that clinician visit.

For Second Life group visits: To preserve the integrity of our study protocol, if the Second Life platofrm is down for any reason, we will continue to conduct the one-on-one clinician visits with the clinician is the Doxy.me platform, as that platform does not require Second Life to be functioning. We will also include telephone "visits" with the study clinician as a (worst case scenario) option should if there is limited internet function (i.e. during a winter storm). The content and activities covered in the rest of the group visit will be included in the next group visit. If all platforms happen to be down due to a mass outage, we will discuss the possibility of rescheduling the entire group session for another date.

Data Collection*

*Please note: The data collection windows described below will be applied to all participants for the purposes of auditing and reporting, as previous windows were not clearly described.

**Data collection blood draws for Spanish-speaking participants (in the East Boston groups) will take place not at BMC, but at the East Boston Neighborhood Health Center phlebotomy labs.

<u>Baseline:</u> Following informed consent, the participant will complete a baseline survey of demographic data such as age, race, income, education, marital status, family status, housing, etc. Baseline clinical parameters will be measured by trained staff from BMC Yawkey Blood Draw Center. We will examine serum blood tests at baseline, including HbA1c, blood pressure and BMI. We will also conduct baseline surveys to collect information about diet, physical activity, computer and health literacy, depression, etc. Baseline vitals (blood pressure, weight, height), baseline accelerometer use*, and the baseline blood draw data collection window opens 30 days prior to the start of the first group session. This is to ensure that baseline information is the most accurate. If a participant has an A1C blood draw done by a health care provider the same day as her baseline A1c would be taken for study purposes, we will use the results from the provider, so as not to place the undue burden on a participant of having two blood draws for the same purpose on the same day.

In order to be eligible for our study, participants must have a *qualifying* A1c ≥ 8.0 (up to 90 days prior to the start of the intervention). However, participants will not become ineligible if their *baseline* A1c results (taken within 30 days of the first group session) drops below 8.0

<u>Post-intervention</u>: The surveys collecting data about diet, physical activity, computer and health literacy, depression, etc will be administered to each participant at the post-intervention (or 9-week) mark. Vitals collection (blood pressure, weight, height), week-long accelerometer use, and post-intervention blood draw will also be collected.We will make every effort to collect our outcome data with a 28-day window (post-group visit 8), but will allow data to be collected up to 56 days post group visit 8 (please see Table 1 below). If the study participant does not respond or attend the data collection time-point after three telephone calls or voicemails, we will mail the participant a letter asking them to reach out to study staff.

<u>6-month:</u> The surveys collecting data about diet, physical activity, computer and health literacy, depression, etc will be administered to each participant at the 6-month time point. Vitals collection (blood pressure, weight, height), week-long accelerometer use, and 6-month A1c blood draw will also be collected. The 6-month data collection window begins **173 days** after the first group visit. We will make every effort to collect our outcome data with a 28-day window (173-201 days from group visit 1), but will allow data to be collected up within a 56-day window (173-229 days from group visit 1). Please refer to Table 1 below. If the study participant does not respond or attend the data collection time-point after three telephone calls or voicemails, we will mail the participant a letter asking them to reach out to study staff.

Table 1. Post Intervention Data Collection Windows

	Ideal	Will allow

9 week	28 days post GV 8	Up to 56 days post GV 8
6 month	173 -201 days from GV 1	173 – 229 days from GV 1

Under extenuating circumstances, we will allow for the use of use provider-drawn A1c results for data collection timepoints (provided that the results are within the appropriate data collection windows). The PI will use discretion to determine when patients' provider-drawn A1c results can be used (typically, we require patients to have a study-specific blood draw). Allowing provider-drawn A1c results to be used for alltime points (under extenuating circumstances/ PI discretion) will allow us to collect A1c data we might not be able to collect otherwise (from women with transportation issues, for example), as well as eliminate undue burden on certain participants for whom an additional blood draw poses a physical or emotional challenge. We will document when provider-drawn blood results are used.

13.2 Outcomes

Describe anticipated primary outcome and any secondary outcomes and how they will be measured:

The RCT's primary outcomes will be the average change in HbA1c (%) from baseline to six months among all participants by study arm and the average change in total physical activity (MET/hr) from baseline to 6 months of all participants by study arm. Secondary outcomes for the RCT portion of the study will include changes in patient activation (based on PAM 13 scores); depression scores (based on PHQ-8); changes in hypertension, and body weight; health-related quality of life (Q-LES-Q screening survey); dietary habits (dietary habits survey), functional status (Sheehan Disability Scale, Promis-29), stress (Perceived Stress Scale Survery, PSS-10), Social Support (MOS Social Support Survey), health service utilization (measued by self-report and chart review), and diabetes distress (diabetes distress screening scale).

For BMC patients ONLY, we will collect AGGREGATE counts from the CDW for pre-baseline health utilization data, which will include ED visits, Observation visits, and hospital admissions. The data window will begin 6 months prior to the start of the intervention and end at the start of the intervention. We have confirmed with Linda Rosen that these counts are not considered linkable to patients in any way since each arm of our study has >6 instances of utilization and that this information is therefore not PHI.

For BMC patients ONLY, we will receive matched controls via EMR query in order to compare the characteristics of participants who enrolled in the study versus participants who did not enroll in the study.

Participants will be matched according to our baseline data collection window dates, for each of the cohorts. For example, controls from Cohort 1's IRB-approved recruitment window (11/18/2016- 2/9/2017) are matched to participants enrolled for Cohort 1. The entire recruitment period ran from 11/18/2016 – 10/24/2019.

The matched controls will include potential participants who did not enroll but were identified by EMR query during recruitment. Potential participants who declined to participate in the Women in Control study or were deemed ineligible will not be selected as controls. A list of three matched controls will be selected for every enrolled participant. They will be matched with enrolled participants based race/ethnicity and exact age when possible.

If 3 controls are not identified by exact age, we will then identify controls by age range in 5-year increments. For example, participant X is a 39-year old Black/African American female, our recruitment pool only includes two Black/African American 39-year-olds. We will select one additional control who is between the ages of 35-39.

Using the matched controls, the following characteristics from the CDW EMR query will be obtained.

- Utilization (#ED/inpatient/obs visit) 6 months prior, 3 months prior, 3 months post, 6 months post index date
- Prior participation in a DSME program (using CPT and/or billing codes) 12 months prior to index date
- HbA1c value reported 1 month prior and 6 months post-index date.

We define the index date as the date of the first group visit for each cohort.

The primary outcomes of the qualitative ethnographic portion of the proposed study include the following: An assessment of technology's influence on learning and skill and development (Positive Technological Development Questionnaire, PTDQ); a characterization of participants' virtual world resource use, user

patterns, and peer interactions in SL (by means of studying researchers' field notes and session audio recordings); patients' perceived DSM educational experience (via key informant interviews and PTDQ responses); and patient-clinician interactions in SL Group Visit (SL clinician consultations and key informant interviews). Exploratory outcomes for the qualitative ethnographic portion of the study will include changes in social network metrics (measured using Social Network Diagnostic Tool).

13.3 Data Analysis

Provide a description of your plan for data analysis. State the types of comparisons you plan (e.g. comparison of means, comparison of proportions, regressions, analysis of variance). Which is the PRIMARY comparison/analysis? How will the analyses proposed relate to the primary purposes of your study? If you are doing qualitative research please state how comparisons will be made.

Measures and Timeline for Outcome Assessment and Statistical Analysis

Primary Outcome Measures:
We will use Actigraph accelerometers to assess participants' physical activity levels and measures of HgbA1c (%) from blood samples analyzed at the BMC Blood Draw labs. The primary outcomes will be the average change in HbA1c (%) from baseline to six months among all participants by study arm and the average change in total physical activity (MET/hr) from baseline to 6 months of all participants by study arm. For pre-intervention physical activity assessment, we will use accelerometry readings for the 1 week period before the 8 week group visit sessions commence. We will again collect accelerometry readings for the 1 week period immediately following the 8-week intervention and then for 1 week at six months from baseline. Four valid days of accelerometer wear are required for an adequate assessment with valid days determined according to published protocols. A daily journal of usage of the acceleromter will be kept by participants to correlate data readings with description of usage. The accompanying computer software uses a combination of the work energy theorem and Freedson equations to calculate total activity counts, total steps, and minutes of light, moderate, hard, and very hard activity (User's Manual, www. theactigraph.com). Similarly, we will assess HbA1c levels obtained from all participants prior to the commencement of the intervention, at the end of the 8-week intervention, and at 6 months post enrollment.

Secondary Outcome Measures:
Secondary outcomes and covariate measures, including patient activation (PAM 13), medication adherence, depression (PHQ-8), clinical parameters (systolic and diastolic blood pressure, and weight /BMI), dietary patterns, stress levels (Perceived Stress Scale), functional status (Sheehan Disability Scale, Promis-29), health literacy, social support, social network changes, quality of life, health service utilization, and diabetes distress, will be compared between the baseline, 9-week, and 6-month time points. We will also collect process measure data to monitor recruitment activities and the fidelity of the intervention and control arm instruction. Process measures that will be tracked include recruitment data (number of eligible participants, number approached, number enrolled, number declined, number found ineligible), intervention adherence (sessions completed/participant) by study arm, completion of computer skills training, data on any participants lost to follow-up. All face-to-face sessions will be audio recorded and virtual world sessions will be audio and video recorded using Zoom. A random sample of recordings will be reviewed by the research team to ensure fidelity of the intervention in each study arm. Additionally, we will assess participant satisfaction with the curriculum, as well as the participants' confidence in using computers, and self-reported adherence to self-monitoring of DSM behaviors. We will survey participants regarding perceived computer skill literacy, time spent in the virtual world, time spent on other computer-related activities, and confidence in using the technology.

Primary Statistical Analysis:
We will compare baseline socio-demographic and clinical characteristics of SL and face-to-face study arms using Chi-square tests for categorical and dichotomous variables and two-sample t-tests for continuous variables. We expect the randomization process to eliminate imbalances in the baseline characteristics. Any baseline measures that are significantly different between interventions and controls will be considered as potential confounders in outcome analyses. Specifically, we strive to balance the language variable (English vs. Spanish) by conducting stratified block randomization. The baseline to 6-month changes in physical activity (MET/hr) and HbA1c will be co-primary outcomes of this study. Since the two measures are highly correlated, no adjustment for multiple testing is necessary. Intention to treat (ITT) and per-protocol (PP) analysis results will be compared for both outcomes utilizing non-inferiority margins of 0.7 for HbA1c and 12 for total physical activity. The per-protocol analysis will be considered primary (more conservative for a non-inferiority analysis), where only those adherent to their study assignment will be included. Adherence to the study assignment will be defined as the attendance of 6 out of the 8 group sessions (virtually or face-to-face). For the ITT analysis, the last value carried forward method will be used to fill in missing follow-up data. We will utilize unadjusted linear regressions with one-sided confidence intervals to compare the change in PA levels and HbA1c from baseline to 6 months between the interventions and controls. The results of the ITT and PP analyses will be reported (mean differences, standard deviations, onesided 95% confidence intervals, t-statistics and p-values), and the PP results will

be considered primary. If the Second Life group proves to exceed the face-to-face group in HbA1c and physical activity improvements, we will assess for superiority. We will stratify the analysis by the language (English vs. Spanish) in which the virtual world or face-to-face group medical visits were conducted. Other potential effect modifiers will also be considered as stratifying factors. If any imbalances in baseline characteristics occur, we will adjust the primary analysis for the potential confounders using linear regression modeling.

Secondary Outcome Analysis:
We will compare the SL and face-to-face conditions with two-sided tests for the secondary outcomes. We will test the superiority of the Second Life intervention over the face-to-face control group in increasing patient activation between baseline and 6 months. Patient activation, measured with the PAM-13 tool, will be analyzed as a categorical variable, where ranges of the PAM score determine stage of activation (Stage 1 (score 39-41) Believes Active Role Important; Stage 2 (scores 42-47) Confidence and Knowledge to Take Action; Stage 3 (scores 50-51) Taking Action; Stage 4 (scores 52-53) Staying the Course under Stress). We will utilize the McNemar tests to compare the shift in activation stages (baseline-6 months) within the two intervention groups, and report the Pearson Chi-Square test comparing the kappa agreement coefficients between the interventions and controls.   Dietary patterns will be assessed using a dietary habits survery at baseline, 9-week, and six month follow up, and will be reported using descriptive statistics for the baseline and 6-month time points. For continuous measures and scores (PHQ8, Sheehan Disability Scale, SBP, DBP, BMI, Promis-29, diabetes distress scale) we will compare the baseline to 6-month average differences between the SL and face-to-face groups using linear regression models. For each continuous outcome, we will first regress the outcome difference on the treatment assignment only, and then adjust the regression for the baseline value. For all outcomes, we will conduct stratified analyses by language (English vs. Spanish) to assess for effect modification. We will use multiple imputation methods to assign missing outcome values. We will perform all analyses with SAS 9.3.

Given the length of our survey questionnaires, the voluntary nature of answering questions, and the fact that data collection forms are completed by participants (unless indicated otherwise), participants may choose not to answer questions and missing data is to be expected. Our study procedures and internal checks do, however, ensure that all forms are reviewed immediately upon completion to maintain data integrity.

For post-intervention data collection time-points, study staff will make every effort to collect data within a 28-day data collection window, however we will allow data to be collected up to 56 days (see Table 1 in Section 13.1). Although we will consider date of data collection in analyses, all data collected within the two month period will be usable and generalizeable.

Patient Advisory Group: Primary and secondary outcome measures will not be collected on patient advisors. The research team will record their name and contact information, for study purposes of convening the PAG. PAG sessions will be audio recorded, however will be stripped of all identifiable information. Recordings will be deleted once all sessions are transcribed, verified for accuracy and analyzed.

13.4 Sample Size/Specimens

How many subjects (or records, or specimens, or charts) will be enrolled in this study? Be sure to include all subjects who will be consented - even those who will be disqualified following consent because they did not meet the enrollment criteria.

Subjects under BMC/BU Medical Center PI
400

Sample Size Justification

Describe how you will have access to a population that will allow recruitment of the necessary number of subjects. Indicate why you chose the sample size proposed. Provide your sample size calculations. If this is a pilot study, this justification does not necessarily require a formal sample size calculation, but should provide a rationale for choosing the sample size proposed (e.g. to estimate a mean to a certain accuracy, to determine if the response rate is above a certain percentage, etc.) Note: Once the IRB approves a certain study sample size then you may not enroll beyond that sample size without first obtaining approval from the IRB. Explain how many evaluable subjects you will need to end up with to answer your study question and how many subjects you will need to enroll and consent to achieve this number.

PAG members: We aim to have 10-15 patient advisors to the Women in Control Study. Due to the time commitment needed for the PAG, we anticipate that some of the women who originally consent to the PAG will not show up consistently to the meetings. For this reason, we will aim to recruit 25 women, so that we may meet our goal of a minimum of 10 women completing the full PAG responsibilities.

RCT Participants:
Participants will be considered "complete" (i.e. data can be used for outcomes analysis) if they have enrolled, attended at least 1 group session, and have completed both the 9-week and 6-month follow up visits.

This study will consider the discrete difference in the average change in physical activity and HbA1c by study arm to be co-primary outcomes. We will first calculate the difference in physical activity levels from baseline to 6 month follow up for each participant. We will then determine the average change across all participants per study arm. Then we will compare the average change in physical activity level between the intervention (SL arm) and control (face to face) arms. We will do the same calculation for change in HgbAc1. We will aim to establish non-inferiority of SL to face-to-face on both accounts. We utilized outcome data obtained with the Women In Control 1.0 pilot study to estimate the sample size necessary to establish non-inferiority of SL to face-to-face in reducing HbA1c (%) and increasing total physical activity levels (MET/hr). We used pilot data due to the paucity of available data determining accelerometry cutoff points for populations with chronic disease. While the results of the pilot study indicate a significant superiority of the Second Life intervention over the face-to-face control in physical activity, we will be conservative in our methods and test for non-inferiority due to the variability in the physical activity data (standard deviation of 35). If the improvements in the outcomes in the Second Life group exceed those in the face-to-face group, we will assess the superiority of the intervention condition over the control. The results of the pilot indicate that the SL group and face-to-face group had roughly similar discrete reductions in HbA1c on average from baseline to 6 months (-0.46 and -0.31, respectively, with a common standard deviation of 2). Assuming a non-inferiority margin of 0.7, a pooled standard deviation of 2, alpha of 0.05, and targeting a power of 80%, we require 102 participants per study arm to show non-inferiority of SL to face-to-face to account for a clinically meaningful decrease in HbA1c. Total physical activity, measured in MET/hr, increased significantly more from baseline to 6 months for the SL group when compared to the face-to-face group (6.70 vs. -9.20, respectively), with a pooled standard deviation of 35.We will assume a non-inferiority margin of 12, and under the assumption of a pooled standard deviation of 35, alpha of 0.05, and target power of 80%, we will require 106 participants per group, for a total of 212. Considering that the dropout rate for the pilot was low (5%), we expect the dropout rate for this study not to exceed 7%. With this consideration, we will enroll and randomize 228 participants, and expect to retain 212.

**Amended: Given higher than anticipated attrition rates, we now recognize that we will need to enroll a greater number of participants in the RCT in order to reach our sample size of 212 for our per protocol analysis.

13.5 Study Attachments

You must attach to this application all surveys, interviews, questionnaires, focus group outlines, etc. that will be used in this study. The IRB must review these materials. If these items are included as part of the attached protocol they do not have to be submitted again. Failure to provide this information could result in a delay in IRB review. If some of the materials are not finalized- submit the DRAFT versions. The final versions will need to be approved by the IRB via an amendment PRIOR to use.

Version	Sponsor Version	Title	Category	Expiration Date	Document Outcome	Checked Out	View Document
No Document(s) have been attached to this form.							

14.0 Risks & Benefits & Justification for Approval

14.1 Potential Risk/Discomforts

List the reasonably foreseeable risks or discomforts to subjects as a result of their participation in the research. Be sure to include physical harms, discomforts, hazards, inconveniences as well as the potential for any social harms (e.g. loss of job or insurability due to breach in confidentiality). For each risk listed be sure to describe the magnitude (seriousness) of the risk, the probability of occurrence, and the potential duration.

PAG members: We do not anticipate PAG members will encounter any risks or discomforts. As with RCT participants, their participation is voluntary, and minimal PHI will be collected on them (name and contact information), which will be password protected at all times, accessible only by research staff.

RCT particpants: Participation will be strictly voluntary. The potential risks are minimal given the fact that the intervention promotes evidence-based diabetes self-management education and incorporates low-risk/ minimal movement (i.e., walking in place and chair yoga). This type of activity is low-risk and does not warrant PCP approval and/or cardiac clearance. We have consulted with Gary Balady, MD, the director of Preventive Cardiology at BUSM, and he has confirmed that the study activities represent "safe low, intensity activity" that do not raise risk concerns. Moreover, low-risk, minimal movement activities are no more intensive than the "exercise-based cardiac rehabilitation" that has been found to reduce mortality rates, as described by Thompson et al. (2003) in their Statement From the Council on Clinical Cardiology (Subcommittee on Exercise, Rehabilitation, and Prevention), which was reviewed and endorsed by the American Heart Association. Aside from low-risk movement activities, there are no known risks for participants participating in this study beyond the potential for embarrassment and/or mental anguish associated with discussing the subjects' histories of chronic illness and interactions with the healthcare system, as well as the breech of privacy and confidentiality that some questions might cause. Protections against risk of breach of privacy/confidentiality for participant self-reported information are described in Section 15.2 below. Additionally, participants might experience discomfort during phlebotomy, however this risk is again, minimal. The participants will be able to contact the investigators and/or their primary care providers for advice on any potential study-related problems that might arise. Dr. Mitchell will review regularly all issues brought by subjects to the investigative team, looking for systematic trends suggestive of risk and clinically important issues. Any potential trends suggestive of risk will be referred to the Data and Safety Advisory Committee. Clinically important concerns will be referred to subjects' primary care providers. The chief risk associated with this protocol is that of a breach of confidentiality and privacy rights relating to protected health information. These potential risks are considered very slight, given the protections that have been put in place (see Section 15.2). Participants will already have a health provider or will be referred to a primary care provider. If a participant experiences emotional distress while interacting in the virtual platform, she will be able to signal to the research staff to initiate a human call to the participant for counseling, support, and potential referral. Any potential risk of the interventions will be minimized by the methods used to develop and teach the protocols. Every effort will be made to make the protocols safe and standardized. Participants will be reminded regularly to inform study staff if they experience any adverse effects.

Because all participants have a diagnosis of uncontrolled Type 2 diabetes, it is likely that the following expected adverse events may occur at one or more time points during the patient's participation in the study over 6 months: hyperglycemia (high blood sugar), hypoglycemia (low blood sugar), dehydration, an infection (i.e. yeast infection, urinary tract infection, oral/dental infection, etc.), neuropathy, retinopathy, kidney failure, cardiac disease/cardiovascular conditions, diabetic ketoacidosis, depression or anxiety, hypertension (high blood pressure), hypotension (low blood pressure), dizziness, sweating, irritability, headache, increased urination, increased thirst, gastrointestinal symptoms including but not limited to nausea, vomiting, abdominal pain, diarrhea, constipation, and/or ED/hospitalization due to adverse side effects from diabetes medications. We will be tracking and monitoring all expected, unexpected, related, unrelated, and serious adverse events using the Adverse Event Description form and the Adverse Events tracker and will report to the IRB during each continuing review or sooner if it is a SAE or unanticipated problem.

Provide a description of how risks will be minimized including, if appropriate, the availability of medical or psychosocial resources that subjects might need as a consequence of the research.

Protection Against Risk at Recruitment Stage:

All recruitment procedures will strictly adhere to HIPAA regulations. Community health center sites will designate a provider or staff member to act as a liaison or "champion" to the study staff, help publicize the study, and coordinate logistics. We will give brief presentations about the study to community health center providers and staff during their regularly scheduled meetings. We will place

advertisements for the study in exam rooms, waiting rooms, nursing stations, the health center's surrounding neighborhood, and local newspapers.

For potential participant's who are are eligible based on the phone screening but have not had a recent Hemoglobin A1c test, they will be asked to come in to BMC to have their blood drawn. The risks and discomforts of drawing blood include temporary discomfort from the needle stick, the possibility of pain or bruising at the site of the blood draw, occasional feelings of lightheadedness and, rarely, infection at the site of the blood draw.

Our inclusion/ exclusion criteria are sufficiently stringent to exclude patients with medical conditions that increase their risk profile and for whom even low-risk physical movement might be dangerous. For example, we do not enroll patients with chronic heart conditions like heart failure, atrial fibrillation, unstable angina, or abnormal heart rhythms or with recent history of acute coronary events; nor do we include patients who have experienced a stroke in the past 6 months.

Protection Against Loss of Privacy and Confidentiality:

All end users of BMC data systems are required to sign and abide by the BMC Information and Systems Confidentiality and Usage Agreement. Under this agreement, the unauthorized possession, use, copying, reading or transmitting of paper or computerized medical records or the disclosure of any information contained in the medical record to unauthorized persons (including unauthorized employees, staff, students, or volunteers) is strictly forbidden. Information generated through contact between patients and healthcare providers at the hospital is privileged and confidential. This privilege extends to all forms and formats in which the information is maintained and stored, including, but not limited to, hard copy, photocopy, microfilm, or automated/electronic form. All persons accessing patient records must adhere to the following guidelines: (1) Information in a patient's record cannot be disclosed without the patient's knowledge and consent; however, there are occasions when there is a legal obligation or duty to disclose information. Requests for patient information from external sources must be directed to the Medical Records Department; (2) Paper medical records must be signed out by an authorized person whenever they are removed from the department; (3) All paper records must be returned to the Medical Records Department; (4) Medical records must not be left unattended where unauthorized persons might read them (access to business information, including BMC billing information, is also granted on a need-to-know basis). Every user must sign the Information and Systems Confidentiality and Usage Agreement before access to any computer system will be granted; this includes medical students, volunteers, consultants, business partners, and vendors who access our data. Some departments may require additional permission before access to a specific system is granted. In addition, risks to confidentiality of the data collected throughout the proposed study will be addressed as follows: all information in the database will be indexed by subject identifier only, so that even if the database server is compromised, subjects cannot be identified, thus maintaining the privacy of their information. Also, assurance and confidentiality of information will be made to all participants.

Data will be handled with the same confidentiality accorded to patient's medical records. Specific procedures protecting participant confidentiality will be as follows: (1) ID number only will be placed on electronic (or paper) study forms or records on which data are collected and/or stored; (2) Access to data files will be secured with a password-filing system (that logs entry) and is restricted to authorized staff only; (3) Necessary hard-copy records containing study data of any type will be kept in locked files; (4) Master lists linking participant information with ID number will be numbered consecutively and prepared before data collection (to ensure accurate accounting). These lists will be kept locked, in duplicate, with access only by the PI and the other investigators; (5) All project staff will sign an oath of confidentiality to ensure their understanding of the terms of confidentiality required. They will be trained in specific procedures to ensure confidentiality; (6) Sign-out procedures for all access to data files will be strictly enforced; (7) All reports and publications will preserve the subjects' anonymity. Any breach of confidentiality will be subject to a root cause analysis and preventive measures taken as appropriate. In addition, outbound communication will include confirmation that the phone call is reaching the intended person, and not someone else that may have picked up the phone.

Protection Against Phlebotomy Related Risks:

The common risks associated with phlebotomy will be minimized by utilizing a laboratory testing service that employs trained phlebotomists. Our research group will not be performing any blood draws, and only trained professionals will be responsible for the drawing and handling of blood.

14.2 Potential Benefits

Describe potential benefit(s) to be gained by the individual subject as a result of participating in the research. (Payments to subjects should not be included in this section.) If there are no direct benefits to individual subjects, you must include a societal benefit that may result from this study.

All study participants will have the opportunity to receive evidence-based diabetes self-management education at no charge. Participation in this study may provide patients with practical knowledge for managing their chronic medical condition and improving their relationships with their healthcare providers. Additionally, participants may experience improvement in diabetes symptoms.
Those individuals assigned to the intervention group will also receive a training course at Boston Medical Center on how to use the computer to access virtual reality technology.

The problem of successful delivery and uptake of diabetes self-management education for Black/African American and Hispanic/Latina women has been well described. Unfortunately, successful intervention models have not been established. The study at hand harnesses the opportunities of a novel health information technology system using Web 2.0 technology (SL) in a rigorous randomized clinical trial offered by a team of investigators that has exhibited the capacity to conduct successful interventions. If effective, the proposed intervention can be disseminated broadly to improve health and reduce disparities chronic disease self-management as well as improve the transition to the Patient-Centered Medical Home model of care. If the proposed intervention is not effective, the study will provide important information to investigators and policy makers looking for ways to reduce disparities in chronic disease self-management delivery systems and outcomes, as well as control escalating health costs due to lack chronic disease education.

14.3 Risk to Benefit Ratio

Describe how risks to subjects are reasonable in relation to anticipated benefits:

The risk of the virtual DSM interventions is minimal. Coupled with the potential for patients to experience health benefits, the risk-benefit ratio is therefore favorable. Each participant will also be contributing to a clinical trial, which will answer important questions regarding the effectiveness of the intervention.

15.0 Data & Safety Monitoring

A data and safety monitoring plan (DSMP) is meant to assure that each clinical investigation has a system for oversight and monitoring of the conduct of the clinical investigation. This oversight is intended to ensure the safety of the participants and the validity and integrity of the data. A DSMP should be commensurate with the risks.

A DSMP can be as simple as the investigator reporting Unanticipated Problems, Adverse Events, and Protocol Deviations to the IRB. A DSMP can be as complex as having a Data and Safety Monitoring Board.

A DSMP can include clinical trial monitoring. Clinical trial monitoring refers to the methods used to oversee the conduct of, and reporting of data from, clinical investigations including appropriate clinical investigator supervision of study site staff. Monitoring activities include communication with the investigator and the study site staff; review of the study site's processes, procedures, and records; and verification of the accuracy of the data.

15.1 For more than minimal risk research, your application needs to include a separate Data and Safety Monitoring Plan. For more information, click here. Please check-off one of the options below:

☑ This study is not greater than minimal risk. Unanticipated Problems, Adverse Events, and protocol deviations will be reported to the IRB as required by IRB policies.

☐ A DSMP is attached in a detailed protocol (provide page number in textbox below).

☐ A DSMP is attached in the Study Attachments section below.

15.2 Who will monitor the research for safety of the participants? (check all that apply)

☑ The Principal Investigator at Boston Medical Center or BU Medical Campus who will report all adverse events and Unanticipated Problems to the IRB in compliance with IRB policy, Federal/State regulations, and sponsor requirements (as applicable).
☐ An independent Data Safety Monitoring Board/Data Monitoring Committee
☐ The Sponsor or Funding Agency
☐ Other:

15.3 DSMP Attachments

Here you can attach any Data and Safety Monitoring Plan documents including your DSMP, Data Safety Monitoring Board charter, and any other related documents.

Version	Sponsor Version	Title	Category	Expiration Date	Document Outcome	Checked Out	View Document
No Document(s) have been attached to this form.							

16.0 Recruitment Procedures/Materials

16.1 Recruitment Procedures

Describe in detail how the research population will be identified and your methods for contacting potential subjects and providing them with information about the study.

Note: The IRB has approved informational brochures that you may provide to potential subjects covering topics such as general participation in research and specific research procedures. These brochures do not have to be listed or uploaded in this submission. For access to these approved brochures, click **here**.

Recruitment Plan

Recruitment at BMC
1. Flyers, Communications Emails, Community outreach: The Principal Investigator will present on the Women in Control study to PCPs and providers at BMC and at the HealthNet CHCs. She will provide them with flyers to give to patients who may be eligible and interested. We will also be displaying flyers in clinics at BMC and at the HealthNet CHCs. In addition, we plan on using the BMC /BUMC weekly communications blasts to provide information to BMC/BUMC employees and providers about the study so they may self-refer or refer family/friends or refer patients if they are a provider (please see RCT e-communications announcement). We will also be recruiting in community spaces such as the YMCA, local libraries, community centers, and churches as well as using a recruitment tool called ResearchMatch. Research Match is a free and secure online recruitment tool funded by the NIH to help connect volunteers interested in learning more about and participating in research studies nationwide. We will be able to use ResearchMatch to target local volunteers who identify as interested in learning more about the WIC study.

We will post our flyer and study information (and have community partners share our flyer and study information) on popular social media platforms (i.e Facebook, Instagram, Twitter). Team members will not be posting on their individual pages, but will share the flyers on community pages and on group pages. In order to post flyers on community and group pages (so that staff personal accounts are not used), our team will need to create a private page on these social media platforms (i.e. Facebook, Instagram, etc.). The study staff will have control over the content of these study-specific social media pages and will apply the strictest privacy settings. This page will not be designed for two-way engagement.

We have developed a study website to better engage potential and current research subjects. The URL for this website will be womenincontrol.org. We will share this website with current, former, and potential participants, as well as with community partners.

We will increase engagement and retention by sending out a quarterly newsletter to current and past particpants.

We will be having routine recruitment and community outreach events at different venues. These outreach events will be open to the public and will be shared with current and past participants who have expressed eagerness in continued involvement, as they are in the same community circles as our target population. Examples of outreach events may include diabetes cooking classes at BMC's demonstration kitchen, low-impact movement events at community centers, networking events, and church workshops. We will announce upcoming community outreach events in our newsletter and announce upcoming events in current groups. For past participants who have consented to be re-contacted, we will notify them of upcoming events via letter invitation or phone call. Because past participants have expressed eagerness in continued involvement, we would like to ask current and past participants to voluntarily speak or share their experiences at outreach events.

Patients will be recruited from the BMC Family Medicine Clinic, internal medicine clincs, and other clinics at BMC. We will place advertisements for the study in exam rooms and waiting rooms in the Family Medicine Clinics. The PI and study clinicians in the Family Medicine department will also present to departmental staff, faculty, providers, during grand rounds presentations. Because clinicians are very busy, the study staff will generate weekly reports in EPIC which will provide a list of that patients have upcoming outpatient appointments who meet our initial inclusion criteria (i.e. female, 18 years of age or older, Black/African American and/or Hispanic Latina, diagnosis of Type 2 diabetes, A1c # 8.0 in the last 3 months). Study staff will then contact the providers of each patient who meets this initial criteria (via weekly “huddle” meetings or EPIC flags) to check-in and to have the provider inform the patient of the study & assess interest during the upcoming appointment. When providers meet with their patients, they will inform the patient of the study & assess interest. If the patient is interested in learning more about the study, the provider will give the patient a study flyer and provide a warm handoff to an RA or member of the study team. The RA will meet with the patient after the appointment with the clinician to provide detailed information about the study, assess interested, and to screen the patients to determine eligibility. If the patients in eligible, the RA will schedule a time for the patient to attend an enrollment session. If the patient is ineligible, the RA will explain the reasons why and thank the patients for their interest and time. The provider will be notified of the patient’s eligibility status and will also be notified if and when the patient consents & enrolls in the study.

Patients will also be recruited through flyers and events are community spaces and organizations (i.e YMCA, libraries, community centers, churches, sorority groups), as well as the employee e-communications blasts. We will also use a snowball sampling to recruit new patients through the networks of previous participants. We will have a “refer-a-friend” program where past participants can refer their friends, family, and acquaintances.

Current and past participants (Alumni) will be compensated with $20 per friend that enrolls and attends at least 1 group session, up to three times (max $60). We will be informing current participants and alumni of the refer-a-friend program by word of mouth and when in communication with participants (during reminder calls or scheduling their follow-up visits). The only materials that current participants or alumni will give to potential participants are copies of the WIC2 RCT flyer. They can make a referral and have their friend (i.e. interested potential subject) contact us for the initial phone screening. It is at this time that we would check to see if they were referred from a current participant or alum and this will be noted in the subject's file, should they enroll, and in our enrollment pool tracking spreadsheet. (i.e. XYZ was referred by WIC20XX). We will be screening referred potential participants just as we would non-referred callers.

2. Recruitment Letter: Another method of recruitment will be through BMC’s Clinical Data Warehouse and ReSPECT Registry. The Clinical Data Warehouse (and ReSPECT Registry) will allow research staff to identify eligible patients based on the following search criteria.

Search Criteria:

- Female
- >=18 years
- Black/African American and/or Hispanic/Latina
- Speaks English
- Diagnosis of Type 2 Diabetes (ICD-10 code: E11)
- Hemoglobin A1c >8.0 in past 3 months
    - date of HbA1c
    - value of HbA1c

If found eligible through the clinical data warehouse/ReSPECT Registry, research staff will contact the patient’s PCP to inform them that a letter will be sent from their colleague, the PI, Dr. Suzanne Mitchell, inviting their patient to join the study. Research staff will explain to the PCPs that there are no consequences for their patient if they decide not join the study (see Recruitment Letter). If the PCP's patient does decide to enroll in the WIC study, the PCP will be notified.

This letter will include introductory information and a fact sheet explaining the study (see WIC2 Fact Sheet). The letter will encourage the patient to contact research staff if they are interested in participating. If the patient does not wish to participate, the letter will provide information on how to inform research staff. If research staff has not heard from the patient 7-10 days post-mailing of the PCP letter, research staff will contact the patient to invite her to discuss the study and the consent process. If the patient is interested, the research staff will obtain verbal consent to ask questions that will determine additional eligibility (i.e. stable housing, plans to leave the area, etc). Please refer to the phone screening script for if research staff calls the patient.

3. Inpatient Hospital Recruitment: Given the high prevalence of Type 2 Diabetes among patients at BMC, there are a number of patients who are admitted daily who may meet our inclusion criteria to participant in the study.We will be recruiting on inpatient floors at BMC using the hospital’s daily census, with permission of each floor. We will receive a copy of the hospital's daily census (which is a list of all patients admitted in the last 24 hours) and check the EMR problem list for all women patients whose diagnoses will not exclude them from participating. The daily census list will exclude patients in the ICU, maternity ward, MICO, and trauma patients.

RAs will then check-in with the nurses on each floor and the patient's schedule to visit the patient to assess eligibility, interest, and availability in participating in the study. The RA will use the preliminary screening form to assess eligibility and interest and schedule a time for the patient to come in to enroll in the study if they are found to be eligible initially.

The Principal Investigator confirms the following:

- No direct or indirect remuneration that constitutes an inducement for recruiting or enrolling subjects will be accepted by any member of the research team; and
- No bonus payments based on the rate or timing of subject recruitment or enrollment will be accepted by any member of the research team; and
- Research involving medical services will comply with federal and state anti-kickback laws and applicable anti-kickback polices of Boston Medical Center and Boston University; and

- No payment or financial incentives (finder's fees) will be offered to any healthcare providers for referring patients to research studies.

☑ I Confirm

16.2 Recruitment Material

Attach all study related recruitment documents including, but not limited to, materials such as: posters, flyers, newspaper ads, script for in-person or telephone recruitment (if any). You may download a recruitment script template **here**. Final versions of all materials should be attached. If a video, submit the tape. If a website, provide the URL and attach screenshots for every page. Note that approved brochures downloaded from **here** are IRB-approved and do not need to be attached. PLEASE NOTE: If you are mailing recruitment materials to Boston Medical Center patients, you must ensure that the patient is not on a DO NOT MAIL list. For more information click the (?) icon.

Version	Sponsor Version	Title	Category	Expiration Date	Document Outcome	Checked Out	View Document
No Document(s) have been attached to this form.							

Are you using promotional recruitment materials for your research study?

○ Yes
◉ No

16.3 Recruitment using the StudyFinder website

The BMC/BU Medical Campus Study Finder is a medical campus website that lists research studies for public view. If you are using Study Finder to recruit subjects, you should complete the Study Finder Form in the Submission Forms section of INSPIR.

Will you be listing your study in Study Finder to recruit subjects? If "yes," select "yes" below and complete the Study Finder Form (located in the Submissions Forms section of the Study Management view of INSPIR II - for more information, click **here**).

○ Yes
◉ No

16.4 Will you be recruiting using one or more Boston HealthNet Community Health Centers (CHC)?

◉ Yes
○ No

Provide details below about which Boston HealthNet CHCs will be involved and the activities that will occur at the CHC(s).
See **Guidance for Working with Boston HealthNet Community Health Centers (CHCs) on INSPIR Studies** and the **Boston HealthNet CHC Contact Information List** for detailed instructions on the approval processes involved for adding CHCs to INSPIR studies. Boston Health Care for the Homeless Program requires approval from the BHCHP internal review committee and Boston HealthNet prior to being added to the INSPIR application (see **Guidance**).
For the Dimock Community Health Center and other non-Boston HealthNet CHCs, contact them directly to obtain approval from their IRBs to participate in this study, and attach the approval letter to the submission.

Recruitment at East Boston Neighborhood Health Center (EBNHC)
Recruitment for Spanish-speaking cohorts will occur at EBNHC as the patient population of this health center is largely Spanish-speaking and of Hispanic/Latina origin. All recruitment procedures will strictly adhere to HIPAA Regulations.

Recruitment at East Boston will operate as follows: Our research assistant will work with the EBNHC medical assistant (non study-staff) to review the list of upcoming appointments at the health center for that day to conduct an initial eligibility screen in the patient's EMR. For patients who meet preliminary eligibility criteria, only name and Primary Care Provider information will be recorded. This information will be used to create a list for each health center provider of patients with appointments that day who might be appropriate for the WIC study. At his or her discretion, the provider can refer his/her patient to the study via a "warm hand-off" (should the RA be stationed at the clinic for the day) or via a "hotline"— the telephone number the study staff. The providers will give the contact information of the study team to the patient, who will then be able to make a decision to call study staff should they be interested in learning more about the study. Communication with study staff prior to screening will be patient-initiated.

We will also give brief presentations about the study to community health center providers and staff during their regularly scheduled meetings. We will place IRB-approved advertisements for the study in exam rooms, waiting rooms, nursing stations, and the health center's surrounding neighborhood.

Because it would be a barrier for patients at EBNHC to travel to BMC for group visits and enrollment activities, research assistants will use a space at EBNHC to conduct the enrollments, weekly group visit sessions, and follow-up data collection.

Spanish-Cohort Implementation at East Boston Neighborhood Health Center (EBNHC):
The EBNHC Research Committee and Medical Directors approved the addition of the East Boston Neighborhood Health Center as a new reruitment site for the WIC study. This will accomodate the screening, recruitment, and enrollment of primarily Spanish-speaking, Latina patients to the study, as well as to serve as an implementation site for the weekly group diabetes self-management visits. East Boston's IRB form was submitted to the EBNHC committee and we were approved to conduct our research at EBNHC after formally presenting to the Research Committee and Medical Directors on July 18, 2018.

Because it would be a barrier for patients at EBNHC to travel to BMC for group visits and enrollment activities, research assistants will use a space at EBNHC to conduct the enrollments, weekly group visit sessions, and follow-up data collection. Screening & implementation of the group visits will occur the same way as the English-speaking groups.

16.5 Screening

Does the study require any clinical screening procedures (blood draw, fasting, etc) performed solely for the purpose of determining eligibility in this research?

(●) Yes ( ) No

Will any potential subjects be directly contacted to obtain screening information?

(●) Yes
( ) No
( ) Not Applicable - all screening activities have been completed for this already-approved study

17.0 Screening Procedures

17.1 Describe the procedures that will be used for screening to determine subject eligibility. If screening procedures are described in a separate protocol, indicate the protocol section here.

PAG Screening: If a patient is interested in being a patient advisor, they will contact our research staff on the phone, who will ask them basic eligibility questions like gender, age and diabetes status (see the attached PAG phone screening questionnaire). If eligible, the research staff will schedule an in-person interview with the patient to further assess their suitability for the role. This interview will include open ended questions about the individual's interest in the study, and their availability to participate in the PAG. If the research staff believes the individual would be able to serve as a patient advisor, they will consent the individual into the study. No data on eligibility will be recorded. Eligibility will be assessed orally by research staff. Thus for screen failures, the research team will not retain any of these data.

RCT Screening:

1. Flyers, Communications Emails, Community outreach: For those patients who call to inquire about the study, research staff will provide a brief introduction about the study and obtain verbal consent to ask questions regarding eligibility (screening questions). All responses the screening questions will be based on self-report with the exception of the date and value of the potential participant's last Hemoglobin A1c. WIC research staff will ask participants to provide documentation of their last date and value of HbA1c. Participants can fax or email this documentation to WIC research staff to wic2@bmc.org (with "SECURE" in the subject line).

2. Recruitment letters from CDW list: For those patients who received a recruitment letter, research staff will provide a brief introduction about the study and obtain verbal consent to ask questions regarding eligibility (screening questions). All responses the screening questions will be based on self-report with the exception of the date and value of the potential participant's last Hemoglobin A1c. For those patients that are ineligible, their screening form will be shredded and destroyed immediately.

3. Warm-hand offs from PCPs/providers (at East Boston): Research staff will provide a brief introduction about the study and obtain verbal consent to ask questions regarding eligibility (screening questions). All responses the screening questions will be based on self-report with the exception of the date and value of the potential participant's last Hemoglobin A1c. Research staff will confirm the data/value of the patient's last A1c with their PCP or medical assistant who is assisting generating a list of patients who meet initial eligibility criteria (gender, age, race, diagnosis of Type 2 diabetes, date/value of last A1c).

Often times, patients do not know if they have been diagnosed with a certain study exclusion criterion (i. e. DKA) or do not know the exact date/value of their last A1c test. We have included a section on pg. 3 of the preliminary phone screening form for the RA to ask and obtain permission from the patient to either contact their PCP (via EPIC flag or phone call) or review their EMR to reduce the burden on the patient of confirming certain eligibility criteria.

For example, if the patient does not have a recent Hemoglobin A1c reading up to 3 months/90 days from the start of the intervention plus 2 weeks (14 days), if needed, study staff will have participants check with their PCPs/EMR chart to have an HbA1c blood draw at their next appointment or obtain their permission to confirm with their PCP/EMR chart (the standard of care for someone with uncontrolled diabetes is to have an A1c blood draw every 3 months). This is necessary in order for the patient to have a qualifying A1c result to be eligible for the study as A1c results may change ever 2-3 months. Since this is standard of care, the study will not be providing A1c blood draws to determine eligibility.

*Please note: The 90 (+14 days, for extenuating insurance circumstances)- day A1c eligibility window described will be applied to all participants for the purposes of auditing and reporting, as previous eligibility windows were not clearly described.

For potential non-BMC participants who do not have documentation of a recent HbA1c value, they will be asked to confirm with their PCP (or give permission to research staff to confirm) that they have had an A1c within 3 months of their scheduled enrollment session that was greater than 8.0. These participants will also sign an authorization of release to obtain and share PHI from a non-BMC primary care provider so the study clinician can communicate with the participant's PCP, following our collaborative care model of care.

Each cohort will have multiple scheduled group enrollment sessions for all screened and eligible patients. The preliminary (phone) screenings will ask patients about their availability to attend these enrollment sessions and the two respective days/times for the intervention and control group visits. If a patient is unable to attend the scheduled group enrollment and/or is unavailable to attend two respective days /times for the group visits, the patient will be placed on a list of eligible patients for future cohorts, which will be securely stored. Research staff will ask permission to re-contact the patient during the screening process to check their availability for upcoming cohorts.

For those patients that are eligible, their screening form will be temporarily stored in a locked, secure file cabinet until the enrollment session/formal in-person consent process. If the eligible patient does not consent, their screening form will also be shredded and destroyed. If the eligible patient does consent, their screening form will become part of the patient's file (which will also be stored in a secured, locked file cabinet). The consented patient will then receive a Study ID#. For all patients, contact information will be collected in a separate form. The screening form will be linked by the patient's Screen ID#; however, the contact information sheet will not be linked to the participant's Screen ID#. The contact information sheets will be stored separately from the screening forms, in a locked, secure file cabinet (see the WIC2 Contact Information sheet).

Once the patient is considered eligible to participate in the study, the WIC2 staff will schedule an enrollment session. At the enrollment session the participant will review and sign the informed consent form, be randomized to the control or intervention group, complete the enrollment questionnaire and view an interactive diabetes education simulation. Participants will be given a pre-/post-assessment about the interactive diabetes education simulation to assess how the baseline knowledge about diabetes for each patient and to understand the effectiveness of the simulation at teaching standard diabetes education.

Participants in the Spanish-speaking groups will watch a diabetes education video in Spanish instead of viewing the diabetes simulation.They will not complete pre- or post- video knowledge assessments. Baseline vitals (blood pressure, weight, height) and the baseline blood draw must occur within 30 days of the first group session. This is to ensure that baseline information is the most accurate. All blood draws will take place at the Yawkey blood draw center. Please refer to the phone screening script. Parking vouchers will be provided to patients, if needed. The patient's PCP will be notified of the patient's participation in the study if she consents and enrolls.

Contact information: For patients that wish to be called back at a later time, only their name & phone number will be collected and stored on the patient contact information sheet in a locked, secure file cabinet. For patients who are ineligible and for patients that are eligible but do not consent during the enrollment session, their contact information sheets will be shredded and destroyed. For patients that are eligible and do consent during the enrollment session, their contact information will be placed in the contact sheets binder which is also stored in a secure, locked file cabinet.

The telephone screen is a preliminary screen. All participants will be screened on enrollment day to ensure that current eligibility criteria are met at the time of enrollment. Eligibility requirements at the time of enrollment supersede those at the time of initial screen. We have two separate screening forms (see attached "Enrollment Day Screening Form" and "Preliminary Screening Form") to ensure that all participants are re-screened at the time of enrollment for all time-sensitive inclusion and exclusion criteria.

Informed Consent: For those patients who meet the basic enrollment criteria and have agreed over the phone to come to BMC to discuss further eligibility, the RA will meet with each individual patient to discuss: (1) the purpose of the study; (2) possible randomization to a control group; (3) IRB safeguards; (4) informed consent. RA will review the Authorization to Use or Disclose Protected Health Information form with the patient and ask her to sign it. We will request permission to audio and video record all group sessions for later analysis and to assess fidelity of intervention across sessions and across cohorts. Individual counseling sessions will not be recorded. Bilingual research staff will be available for informed consent of participants who speak Spanish only.

We will conduct a final brief medical exclusion criteria screening of all participants at the time of Baseline Data Collection, as Baseline Data Collection happens within 1 month of the intervention starting (Enrollment can take place up to 90 days before the intervention begins). This baseline screening will be for medical exclusion criteria (i.e., pregnancy, cancer treatment, dx of DKA, recent stroke history, etc.). As part of good clinical practice, we want to ensure that all participants are medically suitable for the intervention as close as possible to the start of the intervention.

17.2 Describe what eligibility data will be stored and how it will be stored, who will have access, and when these data will be destroyed. For screening failures, please detail how and what data will be retained, if any, along with when these data will be destroyed. Please describe whether identifiers are being retained from those who screen out. Please note if contact information is being retained for future research.

SPANISH PAG Screening: We *will* be retaining eligibility data (gender, age, language, diagnosis of Type II diabetes or pre-diabetes, availability) for individuals who participate in the Spanish PAG (see Spanish PAG Screening Form), but this data will not be linked to individual participants in any way. We will retain screening information for those individuals who are eligible, as we need to reserve documentation that we only enrolled individuals who meet our eligibility criteria.The screening forms, however, will be identified according to an identifier that does *not* link to the participant. (i.e., "SPAG1, SPAG2, SPAG3"). We will not retain screening information for individuals who are not eligible for the PAG.

17.3 Describe the consent process for screening activities. Full consent is required if the screening involves clinical procedures or involves obtaining PHI directly from potential subjects. A Brief Screening Agreement may be used for all other screening involving direct contact with potential subjects. For the Screening Questions Full Consent template and the Brief Screening Agreement template, click here.

We will be using a Brief Screening Agreement that will be read as part of our Screening Script (Please see Spanish PAG Screening Form document attached to the study application). We will not be retaining identifiable sensitive information or PHI, and the screening does not involve a clinical procedure.

17.4 Screening Related Documents

Here you can attach any screening scripts, surveys, or forms and other screening related documents. Do NOT attach screening consent forms here; instead, attach them to the Initial Review Form after completing this Study Application.

Version	Sponsor Version	Title	Category	Expiration Date	Document Outcome	Checked Out	View Document
No Document(s) have been attached to this form.							

18.0 Consent Procedures

18.1 Consent Procedures

You indicated in the "Navigation Menu" section that informed consent will be obtained from subjects. Describe in detail the informed consent process. Include:

1. How and where potential subjects will be provided with an opportunity to discuss the information provided to them
2. The individual(s) (identified by role) who will be involved in obtaining consent. If the study involves a drug, device, or surgical procedure, there are **new requirements** for studies submitted for initial approval on or after September 1, 2019: You must either (A) Confirm that a member of the study team who is a Licensed Independent Practitioner (for example, Physician, Dentist, Physician Assistant, Nurse Practitioner) will discuss the purpose, risks, benefits, and alternatives with potential subjects (either by conducting the entire consent discussion or by participating along with another study team member) or (B) Provide a justification for a different process; and
3. How long subjects will have to decide if they want to participate.
4. If the study includes limited- and non-readers and is greater than minimal risk, how you will use an impartial witness or another method to assure and document subject comprehension (such as a quiz or "teach back"), for limited- and non-readers only or for all subjects.

PAG Members

For those patients who meet the criteria for the PAG and are deemed qualified to serve as a patient advisor, the research assistant will initiate the consent process for the PAG and will discuss: (1) the purpose of the PAG and the overall purpose of the Women in Control RCT, (2) permission to contact them by phone, (3) responsibilities and time commitment of the PAG. The patient will then be asked to sign the consent form, and will be given a copy of the consent form, while the research team will keep the second copy for our records. We will request permission to audio record all PAG sessions.

RCT Particiapnts

Pre-screening verbal consent:

Study personnel will obtain verbal consent from participants to be asked a few eligibility questions, to see if they meet the requirements of the study.

Post-screening written consent:

For those patients who meet all criteria for participation, the research assistant in charge of enrollment that day will then initiate the consent process for the trial and will discuss: (1) the purpose of the study; (2) randomization to either the control or intervention arm; (3) IRB safeguards; (4) informed consent; (5) permission to contact them by phone; and (6) permission to review their medical records. The research assistant will answer questions and fully inform the person about the risks and requirements of the study. If the person indicates interest in participation, she will be told that she can stop any time. Next, the interviewer will read the written informed consent document and assess the potential participant's comprehension of the protocol by asking the potential participant to explain in her own words several aspects of the study. The interviewer will explain any part of it the person requests as well as any part the potential participant did not describe accurately. The interviewer will then check to see if the potential participant has understood. This process will continue until the potential participant has exhibited comprehension of the protocol or, if after three rounds of teaching, the potential participant still does not exhibit comprehension, she will become ineligible. The patient will then be asked to sign the consent form, including HIPAA authorization for the release of medical records from their health provider if they are a non-BMC patient so that study staff can create a

collaborative model of care between the study team and the patient's PCP and to identify subsequent admissions that might occur at other hospitals. One signed copy of the consent forms will be given to the patient and another will be kept in a locked file in the research office. We will request permission to audio record in-person group sessions, and audio and video record virtual world sessions for later analysis and to assess fidelity of intervention across sessions and across cohorts. We will not audio or video record individual sessions between patients and clinicians to protect patient privacy. Bilingual research staff will be available for informed consent of participants who speak Spanish only.

The study team will retain our permission to return to a patient’s medical record to confirm eligibility criteria, if need be, and to improve safety monitoring. This permission does not contradict the original Prep to Research form we filled out, nor does it contradict the language in the consent form.

Research assistants will be trained in the conduct of a high quality informed consent procedure and will follow the activities described in the AHRQ *Informed Consent and Authorization Toolkit for Minimal Risk Research* (http://www.ahrq.gov/fund/informedconsent/). This toolkit was developed to facilitate the process of obtaining informed consent and Health Insurance Portability and Accountability Act (HIPAA) authorization from potential research subjects. This toolkit contains information for people responsible for ensuring that potential research subjects are informed in a manner that is consistent with medical ethics and regulatory guidelines. As recommended in the toolkit, potential subjects who are not able to exhibit comprehension of the protocol will not be eligible for enrollment. Several aspects of informed consent will need to be emphasized in this protocol. It will be critical to differentiate the research activities from clinical care and to dispel any therapeutic misconception or sense of coercion that could arise from enrollment that will transpire in clinical settings, as will occur in the current proposal. Clinical staff will not participate in the informed consent process.

18.2 Verbal Consent/Assent - Waiver of Documentation of Informed Consent

Will this research include an informed consent process, but require a Waiver of Documentation of Consent? For more information, click **here**.

- ○ No
- ◉ Yes, because the research presents no more than minimal risk of harm to the subjects and involves no procedures for which written consent would normally be required outside of the research.
- ○ Yes, because the only record linking the subjects to the research would be the consent document and the main risk in the research would be the potential harm because of a breach of confidentiality. Each subject will be asked whether the subject wants documentation linking the subject with the research, and the subject's wishes will govern.
- ○ Yes, because the subjects or legally authorized representatives are members of a distinct cultural group or community in which signing forms is not the norm, the research presents no more than minimal risk of harm to the subjects, and there is an appropriate alternative mechanism for documenting that informed consent was obtained

18.3 Waiver or Alteration of Informed Consent Process

Does the study meet the criteria for a Waiver or Alteration of Consent Process?

- ○ No
- ◉ Yes

In the text box below, please provide study-specific reasons that justify how the study meets each of the following five criteria for Waiver or alteration of Consent. For more information, click **here**

1. the study is not greater than minimal risk; AND
2. that waiving the requirements for informed consent will not adversely affect the rights and welfare of study subjects; AND
3. that the research cannot be practicably carried out without the waiver of informed consent or alteration of the consent process; AND

4. If the research involves using identifiable private information or identifiable biospecimens, the research cannot practicably be carried out without using such information or biospecimens in an identifiable format (for research that is submitted for initial approval on or after July 1, 2017); AND
5. that (if applicable) there is a plan to disseminate pertinent information to study subjects or legally authorized representatives after the study is completed.

The Waiver of Consent request is only for the matched controls analysis:

1. Obtaining a limited data of historical EMR data is not greater than minimal risk. The limited dataset will be password protected and stored on the G drive which is restricted to authorized staff only.
2. The limited data set will only contain one of the HIPAA identifiers (utilization and A1c results dates), will be password protected, will only be available to limited number of research staff, and all identifiers destroyed at the end of analysis. With these protections the rights and welfare of study subjects will not be adversely affected.
3. We are seeking hisotorical data that is 4-7 years old and thus it would be impractible to obtain this information without the waiver of informed consent as these patients may no longer be receiving their care at BMC, moved away, or changed contact information. In addition, if authorization was attempted to be obtained, there would be systematic differences between those who do and do not provide authorization resulting in bias.
4. The limited data set must contain utlization dates to ensure the data falls within the defined windows of interest and can be compared with the RCT participants.
5. Disseminating infomration to the matched controls is not applicable.

18.5 Consent by Substituted Judgment

Do you intend to obtain consent from a Legally Authorized Representative (LAR) for cognitively impaired/decisionally impaired adult subjects?? For more information, click **here**.

○ Yes
◉ No

18.6 Non-English Language Consent Forms:

Will you obtain consent from subjects who are not fluent in English? For more information, click **here**.

◉ Yes ○ No

Describe how and by whom the consent process will be conducted for non-English speaking subjects. Describe how concerns or questions from the non-English speaking subjects will be addressed during the study. (If the study involves more than minimal risk, provisions must be established to respond to concerns or questions of ALL subjects 24/7.)

For our Spanish speaking participants, all study activities will be conducted in Spanish, including screening, enrollment, consent, computer training, DSM 8-week sessions, follow up, and clinician visits. The consent form will be translated in writing to Spanish and reviewed with a Spanish speaking research staff. If any of our Spanish speaking participants have questions or concerns during the study, they may contact our Spanish speaking research staff or talk to our bilingual peer leader and clinician.

What other subject materials, if any, will be provided to subjects and how will these be translated for non-English speaking subjects?

Copies of consent forms and the 8-week curriculum will be provided to all participants. These materials will all be translated into Spanish.

For each Non-English language, add the language to the table below and indicate which consent document you will use (for the list of available short form consent languages see here: http://www.bumc.bu.edu/irb/submission-requirements/special-submission-requirements/non-english-speaking-subjects/short-consent-form-process/):

Language	Translation
Languages: Spanish	(•) Will translate entire consent after the English language version is approved by the IRB.<br>( ) Requesting the use of Short Form.

If you are requesting use of a short form process for any non-English speaking subjects, please either attach the narrative describing the study that will be verbally translated during the consent process or type into the text box below that the entire IRB-approved consent is to be used as the narrative. When the request is approved, the short-form in the requested language(s) and a separate signature page to be attached to the English narrative will be provided in the study documents.

Version	Sponsor Version	Title	Category	Expiration Date	Document Outcome	Checked Out	View Document
No Document(s) have been attached to this form.							

19.0 Privacy and Confidentiality

19.1 Privacy (Privacy refers to an individual's control over who has access to him/herself)

Please check one:

[x] The following measures will be used to protect the privacy of subjects and potential subjects:

- The information that will be obtained from and/or about subjects and potential subjects is the minimum necessary to conduct the study; and
- If any interventions and interactions occur with subjects and potential subjects, they will take place in private settings.

[ ] Other appropriate measures will be used to protect the privacy of subjects and potential subjects (describe):

19.2 Confidentiality of the Data

In the section below indicate how the study will ensure subject confidentiality and privacy on all study data/results, documents, CRFs, and other documents/files:

[x] Study data/results, documents, CRFs, and other documents/files will be identified with a unique study ID #. The study ID # will be linked to a master-code list that contains all study ID #s and direct subject identifiers (i.e. name, address, DOB, MRN, etc). The master-code list will be maintained separately from study files and access limited to the researchers.

[ ] All study data, documents, CRFs, and other documents/files will be recorded as anonymous. There is NO master-code. There will be no reasonable way to link study data and documents to individual subjects, even temporarily AND subject identities cannot be reasonable ascertained via deductive

disclosure.

☐ There is an alternate plan for how subject will be identified in study data, documents, CRFs, and other documents/files. Please specify in text box below.

You have indicated above that Study data/results, documents, CRFs, and other documents /files will be identified with a unique study ID #. Please select one of the options below:

○ Study data/results, CRFs, and other documents/files for subjects who have been assigned a study ID # may also contain subject identifiers that by themselves or when combined with other identifiers, could result in identifying a subject (ex. maintaining paper medical records that contains a subject's name and MRN in a participant's research file.)

◉ Study data, documents, CRFs, and other documents/files for subjects who have been assigned a study ID # will NOT contain any subject identifiers that by themselves or when combined with others identifiers, could result in identifying a subject.

- **Please describe in the text box below how you will secure the data (e.g. how the master-code will be stored relative to the study data).**
- **If the dataset contains protected health information (PHI) or Personal Information (as defined under Massachusetts law) and is being stored electronically, please provide explicit confirmation that it will be stored according to BMC and/or BU policy for secure storage of such data. Please see the (?) Help Icon to the right for the definitions of PHI and Personal Information and for the appropriate storage options for BMC and BU and specify which will be used.**

All data will be coded. The master sheet which links the participants to their study ID numbers will password protected and only authorized study staff will have access.

19.3 Release of identifiable data.

Is identifiable data being released outside of BMC/BU Medical Campus? (e.g. to sponsors, because of mandated reporting, etc).

○ Yes

◉ No

Pertinent findings (related to the aims of the study) and incidental findings (unrelated to the aims of the study): Does the research (including screening) involve any test or procedure done for research purposes only that may yield findings that are of potential health or reproductive importance to the individual subjects (e.g., disease risk, abnormal lab values, imaging abnormalities, genetic results)?

◉ Yes

○ No

○ Not Applicable - no additional research results will be collected for this study.

Please describe whether or not you will communicate such pertinent and/or incidental findings to subjects and/or their physicians. If you will communicate findings, include the criteria for choosing which findings will be communicated (such as analytic validity and known clinical significance/utility), what the subjects will be told during the consent process, including any option to decline the communication, and to whom the findings will be communicated, when, and how. If you will not communicate findings, please provide your reasons.

NOTE: Consent forms must be consistent with the information in this section. For consent form templates that contain language about communication of pertinent and/or incidental findings, click **here**.

Hemoglobin A1c blood draw results will be reported in the "Labs" section of the patients medical record so that the study doctor as well as the patient's PCP can see the baseline, post-intervention, and 6-month follow-up A1c results. The consent forms explains that lab results will be documented in the patients BMC medical record.

Do you plan to share data with a third-party vendor or software application or program? Some examples include transcription services and smartphone apps. Note: sponsors are not considered third parties. Please contact the IRB @ medirb@bu.edu if you have questions about whether this applies to your study.

- ○ Yes
- ● No

19.4 Destruction of Identifiers

If the data are identifiable and/or if a master-code exists, when and how will the data be de-identified or the master-code be destroyed?

Research data will be stored using Research Electronic Data Capture (REDCap), which is accessible online via a secure web portal.

Participants' ID numbers only will be placed on electronic (or paper) study forms or records on which data are collected and/or stored; Access to data files will be secured with a password-filing system (that logs entry) and is restricted to authorized staff only; Necessary hard-copy records containing study data of any type will be kept in locked files; Master lists linking participant information with ID number will be numbered consecutively and prepared before data collection (to ensure accurate accounting). These lists will be kept locked, in duplicate, with access only by the PI and the other investigators.

We anticipate retaining/ storing data through the data analysis phase of the study (Year 5).

19.5 Certificate of Confidentiality from the NIH

Please check one option below. For more information, click **here**.

- ● This study IS funded by the NIH or CDC; therefore, the study automatically has a Certificate of Confidentiality
- ○ This study is NOT funded by the NIH or CDC

Note: Consent forms must be consistent with the above information. For consent form templates that show confidentiality language, click **here**.

20.0 HIPAA Compliance

20.1 Do you need access to protected health information (PHI) without signed authorization from the individual whose information you need?

- ● Yes
- ○ No

20.2 Do you need PHI (without authorization) *only* to identify subjects for recruitment?

- ● Yes

○ No

20.3 Note: All questions below only pertain to data that you are requesting to access without signed HIPAA Authorization from research participants. Do not include information below on data that you will collect AFTER obtaining signed HIPAA Authorization from the participants.

Please indicate your selection criteria for the records: (e.g. all Type 2 diabetics prescribed metformin, all men aged 50-75 with diagnosis of BPH)

All females 18 years or older with type II diabetes who are African American/Black or Hispanic/Latina and have a PCP at at BMC or one of their affiliated health centers.

For matched controls analysis:

Controls: Females 18 years or older with type II diabetes who are African American/Black or Hispanic /Latina and have an A1c => 8. between 11/18/2016- 10/24/2019.

20.4 Indicate what date range is needed for the records: (e.g. 11/14/98-12/1/13)

1/1/2017 (Start of recruitment) - 5/31/2020 (End of study).

Matched Controls Analysis variables will be extracted from 8/18/2016 - 5/19/2020.

20.5 Please list all data variables that are needed from the medical record or attach the file containing the data variables below. NOTE: If you are using the CDW to provide some or all of the data, the variables you list here will be utilized as your official data request by the CDW:

Please see the attached Medical Record Screening - Data Variables (within old Prep to Research form) document.

Matched Controls Analysis:

HbA1c value and date of collection, utilization dates, and type of encounter (in-patient, observation, or emergency department), prior participation in diabetes-self management education, age, race/ethnicity, gender

Version	Sponsor Version	Title	Category	Expiration Date	Document Outcome	Checked Out	View Document
No Document(s) have been attached to this form.							

20.6 Will you be using the Clinical Data Warehouse (CDW) or will study staff be accessing the records?

○ CDW
○ Study staff will access records
◉ Both

20.7 Does your research require access to any of the HIPAA identifiers?

◉ Yes
○ No

If Yes, what identifiers will you be accessing?

1. Name
2. Date of last HbA1c
3. Date of birth
4. Contact information of patient

Matched Control Amendment:

1. Date of HbA1c
2. Date of hospital encounter

20.8 Please describe why the research cannot be conducted without access to protected health information:

Access to PHI without authorization from the patients is necessary in order to better identify and target those who may be eligible for the study, and recruit these individuals. It is not possible to obtain authorization because we are accessing patients' records with the sole purpose of identifying potential participants. To access PHI only after consent is given would require undue burden for those who would be found ineligible. The recruitment phase of the study will progress much more rapidly if the researchers are allowed access to the protected information in order to recruit only patients who fit the inclusion criteria.

Matched Control Analysis:

Access to PHI without authorization from the patients is necessary in order to identify and match the sample to the enrolled participants. If authorization was attempted to be obtained, there would be systematic differences between those who do and do not provide authorization resulting in bias.

20.9 Why is it not practicable to carry out the research if authorization must be obtained from the participants?

It is not practical to obtain authorization from subjects because only patients with specific disease criteria will be eligible for the study. It will be efficient for the research staff to be able to screen participants from a pool only of patients will eligible disease criteria.

Matched Control Analysis:

It is not practical to obtain authorization because it would require staff to consent patients who may no longer be receiving their care at BMC, moved away, or changed contact information.

20.10 What is your plan to protect any identifiable information from use and disclosure by unauthorized parties?

A limited number of research staff will have access to PHI for the purpose of recruitment and screening. After a particpant is found ineligible or does not wish to participate, all PHI will be destroyed immediately (electronic file deleted, paper file shredded). For patients who enroll in the study, the confidentiality of the data is protected by assigning a unique study number (which is different to from the medical record and social security number) for each subject. Only this study number will be recorded on data forms. A master code list linking the participant's identity to the unique study number will be kept in a password protected electronic file. The research staff will have access to records only necessary to carry out their roles and responsibilities. No additional individuals or entities will have access to identifiable research data.

Matched Control Analysis:

A limited number of research staff will have access to the password protected data set for the purpose of analysis. After the analysis is complete, all PHI will be destroyed (spreadsheet from the Clinical DataWarehouse deleted). No additional individuals or entities will have access to identifiable research data.

20.11 When and how will you destroy any identifiers linked to the data?

(Please note: identifiers should be destroyed at the earliest opportunity as consistent with the design of the research study)

For patients found ineligible for the study or who chose not to participate in the study, the limited set of PHI available to research staff at the time for recruitment purposes will be destroyed immediately. Destruction will occur by permanently deleting electronic files from the drive and shredding paper

documents. For participants who choose to consent and enroll in the study, data identifiers will be kept for a minimum of three years after the conclusion of the study before destruction. What will remain will be totally de-identified data that will be kept indefinitely. Again, destruction will occur by permanently deleting electronic files from the drive and shredding paper documents.

Matched Controls Analysis:
The destruction of identifiers extracted for Matched Control analysis will be kept for a minumum of three years. The destruction will occur by permanently deleting the spreadsheet containing dates of hospital encounters and HbA1c blood draws.

20.12 Please affirm the items below:

- [x] I agree that the protected health information will not be re-used or disclosed to any other person or entity, except as required by law, for the authorized oversight of the research study, or for other research for which the use or disclosure of protected health information would be permitted by the Privacy Regulation (45 CFR 164.512)
- [x] I declare that the requested information constitutes the minimum necessary data to accomplish the goals of the research.

21.0 Cost/Payment

21.1 Cost

Please describe the costs of research visits and procedures and who (the sponsor, the subject's insurance, or the subject) will be responsible for these costs. If any research costs will be billable to insurance, the costs to the subjects will include deductibles and co-payments. Costs of travel and/or parking should be included if the study requires additional visits beyond what would be required for standard clinical care.

Potential subjects who express transportation needs and come to BMC for Enrollment and Baseline procedures will be provided with parking vouchers, cab voucher equivalents, or parking reimbursement via ClinCard (if parked at the Crosstown parking garage) for transportation to and from BMC (including PAG members) for all study-related visits. Study funding will cover the clinical tests required of participants; patients will not be billed for the (not standard/ typical care) outpatient laboratory work collected at baseline, at the conclusion of the 8-week session, and at 6-months post-intervention.

21.2 Payment

Will the subject be reimbursed for participating in this study? (e.g. money, gift certificates, coupons, etc.)

- (•) Yes
- ( ) No

Describe the frequency, method, timing, and amount of payment. Please include the total amount paid to subjects and the plan to prorate payment for subjects who withdraw early from the study.

PAG Members: If PAG members complete 80% study activities (8-10 in person weekly sessions, followed by period sessions in Second Life for up to 6 months), ownership of the laptop they were using for study purposes will be issued to them (Value of ~$350).

SPANISH-SPEAKING PAG MEMBERS: All PAG participants will be compensated with ClinCard. They will receive $25/hour, for a total of up to $200 (8 hours).

RCT participants: Enrolled participants will be compensated for their time and effort as follows:
All study participants will receive $25 at the time of enrollment. $25 per group visit attended and $50 for each follow-up data collection time point (after the 8 week sessions have ended and at 6-months). For example, if a participant enrolls, attends 6 group visits and both follow-up visits, they will receive $275. If a participant attends 7 group visits and only one follow-up visit, they will receive $250. For participants that attend all 8 group visits and both follow-up visits, they will have the option to receive either a brand

new Dell Latitude computer or $300.

Depending on the circumstances, participants may be excused for 1 to 2 study visits due to medical or family emergencies. Participants' absences from a group visit and/or follow-up data collection time point can be excused at the discretion of the study PI. Examples of excused absences include family member or participant hospitalization and/or emergency room visit, surgical procedures, and illness. If participants have any unexcused group visit absences or follow-up data collection time point absences, participants will not eligible for the full compensation unless determined at the discretion of the PI.

All participants will receive their compensation for enrollment after they've completed enrollment activities. The remainder of compensation will be received after the 6-month follow-up visit and after returning all study equipment.

We feel this is a reasonable incentive given the time commitment required for participation. Participants who wish to terminate study participation before completion of the study will be asked to return the study equipment
and will be compensated according to the number of sessions and follow-up visits attended.

For those that participate in the refer-a-friend program, the $20 referral payment will be provided via ClinCard. Payments will be processed up to 1 week after the referrers friend attends their first group session. Participants who have referred friends will be tracked in our compensation spreadsheet, which tracks ClinCard payments & time points. Additionally, the WIC ID number of the subject they referred will be documented in this spreadsheet.

The 50 participants randomly selected for focus groups will receive $25 for their participation in the groups. Key informant interviewees will also receive $25 each.

22.0 Biological Sample Collection

22.1 Add New Samples

Sample Collection

Sample

Select a sample type:

Blood

What is the purpose of the sample collection?

We will be collecting blood from each participant for the purpose of tracking their blood sugar control and diabetes control over the course of the study. We will assessing HbA1c levels for each participant.

Will these samples be collected at the same time as clinical samples are being collected?

○ Yes ◉ No

For blood draws, specify the amount drawn at each visit and across the course of the subject's entire participation time.

Approximately 5-10 ml of blood will be drawn from each patient at three separate points over the course of the study.

Will the sample be stripped of identifiers? Check **No** if you will maintain a master-code list linking Unique sample study ID #s to identifiers(An identifiable

sample is any sample accompanied by codes or data that could facilitate 1) re-contacting the subject or 2) gaining access to identifiable private information about the subject)

○ Yes ◉ No

If sample will be released outside BMC/BU Medical Campus:

Will any samples be released to anyone not listed as an Investigator and/or will the samples be sold or transferred to any third-parties? If so, explain who this will be and what information will also be provided with the samples and why (e.g., any identifiable participant information, test results, health information):

All collection and testing of blood samples will be done by Laboratory Medicine at BMC. Our study personnel will never come in contact with the blood samples. Instead, participants will go to the a BMC blood draw lab, have their blood drawn, and the test results will be sent to our research team. The lab requires each blood test has the participants name and date of birth. This information will not be de-identified. Other than the BMC laboratory who will conduct the blood draws, no one else will have access to the samples.

Data collection blood draws for Spanish-speaking patients in our East Boston groups take place not at BMC, but at the East Boston Neighborhood Health Center phlebotomy labs.

If a subject withdraws from the study:

Will the remaining sample be discarded? (If no, please explain why)

Yes, the sample will be discarded if a participant withdraws from the study.

23.0 Study Attachments

23.1 Attach here any remaining study documents that you have not attached in previous sections.

Version	Sponsor Version	Title	Category	Expiration Date	Document Outcome	Checked Out	View Document
No Document(s) have been attached to this form.							
