# Supplementary material for: Effectiveness of an Immersive Telemedicine Platform for Delivering Diabetes Medical Group Visits for African American, Black and Hispanic, or Latina Women With Uncontrolled Diabetes: The Women in Control 2.0 Noninferiority Randomized Clinical Trial
Source: J Med Internet Res. 2023 May 10;25:e43669. doi: 10.2196/43669 (PMC10209787; doi:10.2196/43669)
Supplement: Multimedia Appendix 2 [file jmir_v25i1e43669_app2.docx]

**Multimedia Appendix 2**

Table S1: Characteristics of WIC2 participants with and without baseline HbA1c

Table S2: Balance among WIC2 participants whose eligibility status changed between baseline and first group visit

Table S3· Changes in HbA1c and Physical Activity from baseline to 9 weeks

Table S4: Proportion of Diabetes Distress, Depressive Symptoms, and Patient Activation among WIC2 participants from baseline to follow-up

Table S5: Characteristics of WIC2 participants that fulfilled vs. did not fulfill protocol

Table S6: Controlling for Potential Confounders to Compliance

Table S7: Subgroup Analysis of Changes in HbA1c and Physical Activity by Language

Table S8: Changes in HbA1c and Physical Activity (Unimputed results)

Table S9· Changes in HbA1c and Physical Activity from baseline to 9 weeks (Unimputed results)

Table S10: Group Changes in Secondary Outcomes among WIC 2 Study Participants (Unimputed results)

**Table S1. Characteristics of WIC2 participants with and without baseline HbA1c.**

| **Characteristic** | **Missing**  **(N = 25)** | | **Present**  **(N = 284)** | |
| --- | --- | --- | --- | --- |
| Virtual World study arm | 14 | (0.48) | 144 | (0.51) |
| Age, years (mean, SD) | 53.95 | (13.64) | 55.48 | (10.36) |
| Race (N, %) |  |  |  |  |
| Black or African American | 11 | (0.38) | 188 | (0.67) |
| White | 2 | (0.07) | 27 | (0.1) |
| Other | 7 | (0.24) | 64 | (0.23) |
| Hispanic/Latina/Spanish Ethnicity (N, %) | 8 | (0.4) | 97 | (0.35) |
| Spanish-speaking (N, %) | 3 | (0.15) | 53 | (0.19) |
| Insurance (N, %) |  |  |  |  |
| Medicare and/or Medicaid | 12 | (0.41) | 174 | (0.62) |
| Commercial | 5 | (0.17) | 59 | (0.21) |
| No insurance | 0 | (0) | 4 | (0.01) |
| Education (N, %) |  |  |  |  |
| High school graduate or less | 12 | (0.41) | 140 | (0.5) |
| Any college/vocational/trade school | 7 | (0.24) | 125 | (0.45) |
| Any Postgraduate | 0 | (0) | 14 | (0.05) |
| Employment Status (N, %) |  |  |  |  |
| Full-time | 6 | (0.21) | 69 | (0.25) |
| Part-time | 5 | (0.17) | 39 | (0.14) |
| Not employed | 9 | (0.31) | 147 | (0.52) |
| Financial Insecurity (N, %) |  |  |  |  |
| Does not have enough money to make ends meet | 7 | (0.24) | 154 | (0.55) |
| Annual Income (N, %) |  |  |  |  |
| ≤ $29,999 | 10 | (0.34) | 130 | (0.46) |
| ≥ $30,000 | 3 | (0.1) | 56 | (0.2) |
| Marital Status, (N, %) |  |  |  |  |
| Married | 5 | (0.17) | 69 | (0.25) |
| Single with partner | 1 | (0.03) | 33 | (0.12) |
| Single for any reason | 14 | (0.48) | 178 | (0.64) |
| Healthcare head of household (N, %) | 3 | (0.15) | 115 | (0.41) |
| Has internet access (N, %) | 14 | (0.7) | 170 | (0.61) |
| Has smartphone (N, %) | 14 | (0.7) | 223 | (0.8) |
| Low Health Literacy (N, %) | 4 | (0.2) | 83 | (0.3) |
| Body mass index, kg/m2, mean (SD) | 27.2 | (3.74) | 34.27 | (6.92) |
| HbA1c (mean, SD) | 10.04 | (2.91) | 10.32 | (2.89) |
| PHQ-8 score >5 (mean, SD) ^a^ | 6 | (5.45) | 5.46 | (4.98) |

^a^ Assessed using the Patient Health Questionnaire-8.^29^

**Table S2. Balance among WIC2 participants whose eligibility status changed between baseline and first group visit.**

|  | **In-Person**  **(N = 15)** | | **Virtual World**  **(N = 14)** | |
| --- | --- | --- | --- | --- |
| Age, years (mean, SD) | 53.5 | (11.32) | 54.4 | (16.26) |
| Race (N, %) |  |  |  |  |
| Black or African American | 5 | (0.33) | 6 | (0.43) |
| White | 1 | (0.07) | 1 | (0.07) |
| Other | 4 | (26.7) | 3 | (0.21) |
| Hispanic/Latina/Spanish Ethnicity (N, %) | 4 | (0.4) | 4 | (0.4) |
| Spanish-speaking (N, %) | 3 | (0.3) | 0 | (0) |
| Insurance (N, %) |  |  |  |  |
| Medicare and/or Medicaid | 7 | (0.47) | 5 | (0.36) |
| Commercial | 2 | (0.13) | 3 | (0.21) |
| No insurance | 0 | (0) | 0 | (0) |
| Education (N, %) |  |  |  |  |
| High school graduate or less | 6 | (0.4) | 6 | (0.43) |
| Any college/vocational/trade school | 3 | (0.2) | 4 | (0.29) |
| Any Postgraduate | 0 | (0) | 0 | (0) |
| Employment Status (N, %) |  |  |  |  |
| Full-time | 3 | (0.2) | 3 | (0.21) |
| Part-time | 2 | (0.13) | 3 | (0.21) |
| Not employed | 5 | (0.33) | 4 | (0.29) |
| Financial Insecurity (N, %) |  |  |  |  |
| Does not have enough money to make ends meet | 2 | (0.13) | 5 | (0.36) |
| Annual Income (N, %) |  |  |  |  |
| ≤ $29,999 | 6 | (0.4) | 4 | (0.29) |
| ≥ $30,000 | 2 | (0.13) | 1 | (0.07) |
| Marital Status, (N, %) |  |  |  |  |
| Married | 3 | (0.2) | 2 | (0.14) |
| Single with partner | 1 | (0.07) | 0 | (0) |
| Single for any reason | 6 | (0.4) | 8 | (0.57) |
| Healthcare head of household (N, %) | 1 | (0.1) | 2 | (0.2) |
| Has internet access (N, %) | 6 | (0.6) | 8 | (0.8) |
| Has smartphone (N, %) | 7 | (0.7) | 7 | (0.7) |
| Low Health Literacy (N, %) | 3 | (0.3) | 1 | (0.1) |
| Body mass index, kg/m2, mean (SD) | 28.3 | (3.96) | 26.65 | (4.11) |
| HbA1c (mean, SD) | 10.15 | (1.52) | 9.92 | (1.39) |
| PHQ-8 score >5 (mean, SD)^a^ | 7.8 | (6.09) | 4.2 | (4.29) |

^a^ Assessed using the Patient Health Questionnaire-8.^29^

**Table S3. Changes in HbA1c and Physical Activity from baseline to 9 weeks.**

|  | | **HbA1c, % Baseline to 9 weeks** | | | | | **HbA1c non-inferiority**  **(margin of 0.7)** | |
| --- | --- | --- | --- | --- | --- | --- | --- | --- |
| Analysis Type | Study Arm | Baseline, % [mmol/mol]  (SD) | 9 weeks  [mmol/mol]  (SD) | Change (SD) | Within-group *P* | Between-group *P* | Mean difference (one-sided 95% CI) | *P-*value |
| **PP** | IP | 10.1 [87]  (1.8) | 9.5 [80]  (2.0) | -0.6 (1.2) | 0.0001 | 0.39 | 0.2 (-∞, 0.14) | <0.0001 |
|  | VW | 9.6 [81]  (1.7) | 9.2 [77] (1.9) | -0.4 (1.3) | 0.0076 |  |  |  |
| **ITT** | IP | 10.2 [88]  (1.8) | 9.5 [80] (2.0) | -0.7 (1.4) | 0.0605 | 0.25 | 0.2 (-∞, 0.09) | < 0.0001 |
|  | VW | 9.7 [83]  (1.7) | 9.2 [77] (2.0) | -0.5 (1.4) | 0.1231 |  |  |  |
|  | | **Work week MET-hours Baseline to 9 week** | | | | | **Work week MET-hours non-inferiority**  **(margin of 12)** | |
| Analysis Type | Study Arm | Baseline  (SD) | 6-month | Change (SD) | Within-group *P* | Between-group *P* | Mean difference (one-sided 95% CI) | *P-*value |
| **PP** | IP | 105.3  (35.3) | 107.4 (43.1) | 0.9 (33.7) | 0.7274 | 0.0759 | -8.7 (0.64, ∞) | <0.0001 |
|  | VW | 106.6 (33.4) | 99.9 (37.1) | -7.76 (32.2) | 0.2883 |  |  |  |
| **ITT** | IP | 105.5 (40.2) | 40.2 (106.1) | -0.8 (39.2) | 0.9598 | 0.1613 | -7.7 (-1.39, ∞) | 0.0004 |
|  | VW | 106.4  (37.1) | 97.8 (46.6) | -8.51 (38.4) | 0.4114 |  |  |  |

|  |  | Baseline, N | % | 9 weeks, N | 9 weeks, % | 6 months, N | 6 months, % |
| --- | --- | --- | --- | --- | --- | --- | --- |
| Diabetes Distress ^a^ | *Little or none, <2* | 138 | 47 | 153 | 64 | 160 | 68 |
|  | *Moderate, 2-3* | 87 | 30 | 59 | 25 | 47 | 20 |
|  | *High, >3* | 69 | 23 | 27 | 11 | 30 | 13 |
|  |  | | | | | | |
| Depressive Symptoms ^b^ | *None, 0-4* | 153 | 52 | 148 | 62 | 142 | 6 |
|  | *Mild, 5-9* | 85 | 29 | 49 | 21 | 60 | 25 |
|  | *Moderate, 10-15* | 41 | 14 | 30 | 13 | 26 | 11 |
|  | *Severe, ≥ 16* | 16 | 5 | 11 | 5 | 9 | 04 |
|  | | | | | | | |
| Patient Activation ^c^ | *Level 1* | 38 | 13 | 16 | 6 | 16 | 6 |
|  | *Level 2* | 32 | 11 | 19 | 7 | 20 | 7 |
|  | *Level 3* | 90 | 3 | 76 | 26 | 89 | 31 |
|  | *Level 4* | 136 | 46 | 128 | 44 | 112 | 39 |

**Table S4. Proportion of Diabetes Distress, Depressive Symptoms, and Patient Activation among WIC2 participants from baseline to follow-up.**

^a^ Assessed using the Diabetes Distress Scale-17.^27,28^

^b^ Assessed using the Patient Health Questionnaire-8.^29^

^c^ Assessed using the Patient Activation Measure (PAM)-13.^31^

**Table S5. Characteristics of WIC2 participants that fulfilled vs. did not fulfill protocol.**

|  | **Non-Compliers**  **(N = 102)** | | **Compliers**  **(N = 207)** | |
| --- | --- | --- | --- | --- |
| Virtual World study arm (N, %) | 50 | (0.49) | 108 | (0.52) |
| Age, years (mean, SD) | 54.87 | (11.13) | 55.6 | 10.35 |
| Race (N, %) |  |  |  |  |
| Black or African American | 55 | (0.54) | 144 | (0.7) |
| White | 8 | (0.08) | 21 | (0.1) |
| Other | 30 | (0.29) | 41 | (0.2) |
| Hispanic/Latina/Spanish Ethnicity (N, %) | 38 | (0.41) | 67 | (0.32) |
| Spanish-speaking (N, %) | 25 | (0.27) | 31 | (0.15) |
| Insurance (N, %) |  |  |  |  |
| Medicare and/or Medicaid | 60 | (0.59) | 126 | (0.61) |
| Commercial | 19 | (0.19) | 45 | (0.22) |
| No insurance | 0 | (0) | 4 | (0.02) |
| Education (N, %) |  |  |  |  |
| High school graduate or less | 57 | (0.56) | 95 | (0.46) |
| Any college/vocational/trade school | 33 | (0.32) | 99 | (0.48) |
| Any Postgraduate | 2 | (0.02) | 12 | (0.06) |
| Employment Status (N, %) |  |  |  |  |
| Full-time | 23 | (0.23) | 52 | (0.25) |
| Part-time | 13 | (0.13) | 31 | (0.15) |
| Not employed | 51 | (0.5) | 105 | (0.51) |
| Financial Insecurity (N, %) |  |  |  |  |
| Does not have enough money to make ends meet | 45 | (0.44) | 116 | (0.56) |
| Annual Income (N, %) |  |  |  |  |
| < $29,999 | 42 | (0.41) | 98 | (0.47) |
| > $30,000 | 17 | (0.17) | 42 | (0.2) |
| Marital Status, (N, %) |  |  |  |  |
| Married | 23 | (0.23) | 51 | (0.25) |
| Single with partner | 12 | (0.12) | 22 | (0.11) |
| Single for any reason | 58 | (0.57) | 134 | (0.65) |
| Healthcare head of household (N, %) | 39 | (0.42) | 79 | (0.38) |
| Has internet access (N, %) | 56 | (0.6) | 128 | (0.62) |
| Has smartphone (N, %) | 76 | (0.82) | 161 | (0.78) |
| Low Health Literacy (N, %) | 29 | (0.31) | 58 | (0.28) |
| Body mass index, kg/m2, mean (SD) | 33.11 | (6.51) | 34.51 | (7.08) |
| HbA1c (mean, SD) | 10.29 | (1.05) | 10.29 | (2.13) |
| PHQ-8 score >5 (mean, SD)^a^ | 6.17 | (5.21) | 5.19 | (4.89) |

^a^ Assessed using the Patient Health Questionnaire-8.^29^

**Table S6. Controlling for Potential Confounders to Compliance.**

| **Outcome** | **Arm** | **Within-group** | | | **Between-group** | | |
| --- | --- | --- | --- | --- | --- | --- | --- |
|  |  | **LS Mean** | **St. Error** | **p value** | **LS Mean** | **St. Error** | **p value** |
| HbA1c (PP) | IP | -0.6 | 0.3 | 0.0384 | 0.0 | 0.2 | 0.88 |
|  | VW | -0.4 | 0.2 | 0.14 |  |  |  |
| HbA1c (PP, Imputed) | IP | -0.6 | 0.3 | 0.0361 | 0.0 | 0.3 | 0.97 |
|  | VW | -0.4 | 0.2 | 0.1135 |  |  |  |
| HbA1c, ITT | IP | -0.7 | 0.2 | 0.0022 | 0.1 | 0.2 | 0.67 |
|  | VW | -0.4 | 0.2 | 0.05 |  |  |  |
| HbA1c, ITT (Imputed) | IP | -0.7 | 0.2 | 0.0084 | 0.1 | 0.2 | 0.76 |
|  | VW | -0.4 | 0.2 | 0.0308 |  |  |  |
| MET hours (PP) | IP | 1.2 | 4.9 | 0.80 | -2.4 | 4.0 | 0.55 |
|  | VW | -9.4 | 4.3 | 0.0324 |  |  |  |
| MET hours (PP, Imputed) | IP | 2.2 | 4.9 | 0.65 | 0.3 | 5.5 | 0.95 |
|  | VW | -8.8 | 4.4 | 0.0446 |  |  |  |
| MET hours, ITT | IP | 3.3 | 4.9 | 0.49 | -2.4 | 3.8 | 0.53 |
|  | VW | -8.1 | 4.0 | 0.0452 |  |  |  |
| MET hours, ITT (Imputed) | IP | 0.50 | 4.4 | 0.91 | -0.9 | 5.5 | 0.87 |
|  | VW | -5.2 | 4.5 | 0.26 |  |  |  |

PP: Per-Protocol; ITT: Intention-to-Treat; IP: In-person; VW: Virtual World; MET: metabolic equivalent of task

**Table S7. Subgroup Analysis of Changes in HbA1c and Physical Activity by Language.**

|  | | **HbA1c, % Baseline to 6 months** | | | | | | |
| --- | --- | --- | --- | --- | --- | --- | --- | --- |
| Analysis | Arm | Subgroup | Baseline, mean [mmol/mol]  (SD) | 6 months, mean  [mmol/mol]  (SD) | Within-  Group  *P* | Coef | Std. Error | *P-value* |
| PP, Unimputed | IP | English | 10.0 [86] (1.9) | 9.4 [79] (2.2) | 0.93 | 0.04 | 0.24 | 0.8719 |
|  |  | Spanish | 10.3 [89] (1.6) | 9.4 [79] (1.6) |  |  |  |  |
|  | VW | English | 9.5 [80] (1.7) | 9.1 [76] (1.8) | 0.98 |  |  |  |
|  |  | Spanish | 9.9 [85] (1.7) | 9.0 [75] (1.5) |  |  |  |  |
| PP, Imputed | IP | English | 10.0 [86] (1.9) | 9.3 [78] (2.3) | 0.90 | 0.04 | 0.25 | 0.8895 |
|  |  | Spanish | 9.5 [80] (1.7) | 9.1 [76] (1.9) |  |  |  |  |
|  | VW | English | 10.3 [89] (1.6) | 9.4 [79] (1.6) | 0.98 |  |  |  |
|  |  | Spanish | 9.9 [85] (1.7) | 9.1 [76] (1.6) |  |  |  |  |
| ITT, Unimputed | IP | English | 10.2 [88] (1.9) | 9.4 [79] (2.2) | 0.77 | 0.09 | 0.23 | 0.7101 |
|  |  | Spanish | 10.2 [88] (1.6) | 9.4 [79] (1.6) |  |  |  |  |
|  | VW | English | 9.6 [81] (1.7) | 9.1 [76] (2.0) | 0.81 |  |  |  |
|  |  | Spanish | 9.8 [84] (1.7) | 9.1 [76] (1.7) |  |  |  |  |
| ITT, Imputed | IP | English | 10.2 [88] (1.9) | 9.4 [79] (2.3) | 0.87 | 0.07 | 0.27 | 0.8035 |
|  |  | Spanish | 9.6 [81] (1.75) | 9.2 [77] (2.1) |  |  |  |  |
|  | VW | English | 10.1 [87] (1.7) | 9.4 [79] (1.9) | 0.57 |  |  |  |
|  |  | Spanish | 9.8 [84] (1.7) | 9.2 [77] (2.0) |  |  |  |  |
|  | | **Work-week MET-hours, Baseline to 6 months** | | | | | | |
| Analysis | Arm | Subgroup | Baseline, mean  (SD) | 6 months, mean (SD) | Within-  Group  *P* | coef | std. error | *P-value* |
| PP, Unimputed | IP | English | 100.5 (30.9) | 98.3 (31.0) | 0.28 | 4.12 | 5.45 | 0.4508 |
|  |  | Spanish | 113.7 (33.6) | 104.9 (34.1) |  |  |  |  |
|  | VW | English | 106.7 (33.1) | 97.4 (29) | 0.42 |  |  |  |
|  |  | Spanish | 102.9 (35.0) | 99.9 (32.3) |  |  |  |  |
| PP, Imputed | IP | English | 101.4 (33.2) | 100.0 (35.3) | 0.34 | 5.56 | 6.27 | 0.37956 |
|  |  | Spanish | 107.4 (34.5) | 101.0 (37.5) |  |  |  |  |
|  | VW | English | 115.2 (38.7) | 104.9 (34.1) | 0.66 |  |  |  |
|  |  | Spanish | 104 (38.2) | 99.3 (41.6) |  |  |  |  |
| ITT, Unimputed | IP | English | 103.1 (36.1) | 98.5 (33.2) | 0.17 | 6.32 | 5.08 | 0.2148 |
|  |  | Spanish | 103.5 (35.1) | 104.94 (32.7) |  |  |  |  |
|  | VW | English | 105.8 (32.5) | 96.4 (30.0) | 0.57 |  |  |  |
|  |  | Spanish | 102.4 (35.4) | 104.0 (31.2) |  |  |  |  |
| ITT, Imputed | IP | English | 105.2 (39.9) | 106.2 (45.5) | 0.37 | 5.00 | 5.76 | 0.38628 |
|  |  | Spanish | 107.2 (36.5) | 105.1 (46.4) |  |  |  |  |
|  | VW | English | 106.3 (41.24) | 106.5 (45.5) | 0.96 |  |  |  |
|  |  | Spanish | 104.3 (39.12) | 105.2 (48.3) |  |  |  |  |

PP: Per Protocol; ITT: Intention-to-Treat; CI: confidence interval; IP: In-person; VW: Virtual World; MET: metabolic equivalent of task

**Table S8. Changes in HbA1c and Physical Activity (Unimputed results).**

|  | | **HbA1c, % Baseline to 6 months** | | | | | **HbA1c non-inferiority**  **(margin of 0.7)** | |
| --- | --- | --- | --- | --- | --- | --- | --- | --- |
| Analysis Type | Study Arm | Baseline, % [mmol/mol]  (SD) | 6 months  [mmol/mol]  (SD) | Change (SD) | Within-group *P* | Between-group *P* | Mean difference (one-sided 97.5% CI) | *P-*value |
| PP  N=198 | IP | 10.1 [87]  (1.8) | 9.4 [79]  (2.1) | -0.7 (1.8) | 0.0001 | 0.423 | 0.19 (-∞, 0.28) | 0.0001 |
|  | VW | 9.6 [81]  (1.7) | 9.0 [75] (1.8) | -0.5 (1.6) | 0.001 |  |  |  |
| ITT  N=228 | IP | 10.2 [88]  (1.8) | 9.4 [79]  (2.1) | -0.7 (1.8) | <0.0001 | 0.2799 | 0.24 (-∞, 0.28) | < 0.0001 |
|  | VW | 9.7 [83]  (1.7) | 9.1 [76]  (1.9) | -0.5 (1.6) | 0.0013 |  |  |  |
|  | | **Work week MET-hours Baseline to 6 month** | | | | | **Work week MET-hours non-inferiority**  **(margin of 12)** | |
| Analysis Type | Study Arm | Baseline  (SD) | 6-month | Change (SD) | Within-group *P* | Between-group *P* | Mean difference (one-sided 97.5% CI) | *P-*value |
| PP  N=179 | IP | 103.9 (32.0) | 100.3 (31.9) | -4.1 (33.0) | 0.25 | 0.3196 | -4.73 (-4.62, ∞) | 0.0003 |
|  | VW | 105.8 (33.5) | 98.0 (29.6) | -8.8 (30.3) | 0.0071 |  |  |  |
| ITT  N=207 | IP | 103.2 (35.7) | 100.4 (33.0) | -2.5 (32.3) | 0.43 | 0.2886 | -4.67 (-3.98, ∞) | 0.0001 |
|  | VW | 104.8  (33.2) | 98.3 (30.3) | -7.2 (30.8) | 0.0201 |  |  |  |

PP: Per Protocol; ITT: Intention-to-Treat; CI: confidence interval; IP: In-person; VW: Virtual World; MET: metabolic equivalent of task

**Table S9. Changes in HbA1c and Physical Activity from baseline to 9 weeks (Unimputed results).**

|  | | **HbA1c, % Baseline to 9 weeks** | | | | | **HbA1c non-inferiority**  **(margin of 0.7)** | |
| --- | --- | --- | --- | --- | --- | --- | --- | --- |
| Analysis Type | Study Arm | Baseline, % [mmol/mol]  (SD) | 9 weeks  [mmol/mol]  (SD) | Change (SD) | Within-group P | Between-group P | Mean difference (one-sided 95% CI) | P-value |
| PP | IP | 10.1 [87]  (1.8) | 9.5 [80]  (2.0) | -0.6 (1.2) | <0.0001 | 0.39 | 0.15 (-∞, 0.14) | <0.0001 |
|  | VW | 9.6 [81]  (1.7) | 9.2 [77] (1.9) | -0.4 (1.3) | 0.0011 |  |  |  |
| ITT | IP | 10.2 [88]  (1.8) | 9.4 [79]  (1.9) | -0.7 (1.3) | <0.0001 | 0.09 | 0.29 (-∞, -0.01) | < 0.0001 |
|  | VW | 9.7 [83]  (1.7) | 9.3 [78]  (1.9) | -0.4 (1.2) | 0.0003 |  |  |  |
|  | | **Work week MET-hours Baseline to 9 week** | | | | | **Work week MET-hours non-inferiority**  **(margin of 12)** | |
| Analysis Type | Study Arm | Baseline  (SD) | 6-month | Change (SD) | Within-group *P* | Between-group *P* | Mean difference (one-sided 95% CI) | *P-*value |
| PP | IP | 103.9 (32.0) | 104.3 (36.8) | 2.4 (28.6) | 0.44 | 0.0168 | -10.32 (3.25, ∞) | <0.0001 |
|  | VW | 105.8 (33.5) | 98.5 (32.7) | -7.9 (29.1) | 0.0087 |  |  |  |
| ITT | IP | 103.2 (35.7) | 105.9 (39.0) | 3.0 (29.3) | 0.30 | 0.0103 | -10.22 (3.7, ∞,) | <0.0001 |
|  | VW | 104.8  (33.1) | 98.1 (31.8) | -7.2 (28.3) | 0.0084 |  |  |  |

PP: Per Protocol; ITT: Intention-to-Treat; CI: confidence interval; IP: In-person; VW: Virtual World; MET: metabolic equivalent of task

**Table S10. Group Changes in Secondary Outcomes among WIC 2 Study Participants (Unimputed results).**

| **TOTAL DIABETES DISTRESS** ^a^ **(DD)** | | | Changes from baseline to 9 weeks | | | Changes from baseline to 6 months | | |
| --- | --- | --- | --- | --- | --- | --- | --- | --- |
| Analysis Type | Arm | Baseline Mean (SD) | LS Means (Std Err) | Within-group p | Between-group p | LS Means (Std Err) | Within-group p | Between-group p |
| PP | IP | 2.3 (1.1) | -0.5 (0.1) | 6.00E-04 | 0.0505 | -0.25 (0.1) | 0.0005 | 0.9526 |
|  | VW | 2.3 (1.0) | -0.3 (0.1) | 0.0393 |  | -0.2 (0.1) | 0.0025 |  |
| ITT | IP | 2.2 (1.0) | -0.4 (0.1) | 6.00E-04 | 0.0454 | -0.18 (0.1) | 0.0068 | 0.7602 |
|  | VW | 2.3 (1.1) | -0.2 (0.1) | 0.0816 |  | -0.19 (0.1) | 0.0021 |  |
| **DD: EMOTIONAL BURDEN** | | | Changes from baseline to 9 weeks | | | Changes from baseline to 6 months | | |
| Analysis Type | Arm | Baseline Mean (SD) | LS Means (Std Err) | Within-group p | Between-group p | LS Means (Std Err) | Within-group p | Between-group p |
| PP | IP | 2.7 (1.5) | -0.7 (0.2) | 2.0E-04 | 0.0116 | -0.3 (0.1) | 0.0013 | 0.48 |
|  | VW | 2.7 (1.5) | -0.4 (0.2) | 0.06 |  | -0.2 (0.1) | 0.0111 |  |
| ITT | IP | 2.6 (1.5) | -0.7 (0.2) | 1.0E-04 | 0.0027 | -0.3 (0.1) | 0.0037 | 0.27 |
|  | VW | 2.7 (1.4) | -0.3 (0.2) | 0.06 |  | -0.2 (0.1) | 0.0068 |  |
| **DD: PHYSICIAN** | | | Changes from baseline to 9 weeks | | | Changes from baseline to 6 months | | |
| Analysis | Arm | Baseline Mean (SD) | LS Means (Std Err) | Within-group p | Between-group p | LS Means (Std Err) | Within-group p | Between-group p |
| PP | IP | 1.5 (0.9) | -0.2 (0.1) | 0.21 | 0.14 | -0.1 (0.1) | 0.05 | 0.47 |
|  | VW | 1.5 (1.0) | 0.0 (0.0) | 0.89 |  | -0.01 (0.1) | 0.31 |  |
| ITT | IP | 1.5 (0.9) | -0.1 (0.1) | 0.26 | 0.21 | -0.0 (0.1) | 0.48 | 0.77 |
|  | VW | 1.6 (1.0) | 0.1 (0.1) | 0.73 |  | -0.1 (0.1) | 0.18 |  |
| **DD: REGIMEN** | | | Changes from baseline to 9 weeks | | | Changes from baseline to 6 months | | |
| Analysis | Arm | Baseline Mean (SD) | LS Means (Std Err) | Within-group p | Between-group p | LS Means (Std Err) | Within-group p | Between-group p |
| PP | IP | 2.68 (1.3) | -0.7 (0.2) | 2.0E-04 | 0.08 | -0.3 (0.1) | 0.0002 | 0.79 |
|  | VW | 2.7 (1.4) | -0.4 (0.2) | 0.02 |  | -0.3 (0.1) | 0.0006 |  |
| ITT | IP | 2.6 (1.3) | -0.6 (0.2) | 5.0E-04 | 0.08 | -0.2 (0.1) | 0.0034 | 0.89 |
|  | VW | 2.7 (1.3) | -0.3 (0.2) | 0.05 |  | -0.3 (0.1) | 0.0009 |  |
| **DD: INTERPERSONAL** | | | Changes from baseline to 9 weeks | | | Changes from baseline to 6 months | | |
|  | Arm | Baseline Mean (SD) | LS Means (Std Err) | Within-group p | Between-group p | LS Means (Std Err) | Within-group p | Between-group p |
| PP | IP | 2.0 (1.3) | -0.3 (0.2) | 0.13 | 0.91 | -0.18 (0.1) | 0.0396 | 0.86 |
|  | VW | 2.0 (1.3) | -0.3 (0.2) | 0.03 |  | -0.17 (0.1) | 0.0375 |  |
| ITT | IP | 1.9 (1.3) | -0.2 (0.2) | 0.17 | 0.79 | -0.12 (0.1) | 0.12 | 0.82 |
|  | VW | 2.0 (1.3) | -0. (0.2) | 0.10 |  | -0.16 (0.1) | 0.0425 |  |
| **PHQ-8** ^b^ | | | Changes from baseline to 9 weeks | | | Changes from baseline to 6 months | | |
| Analysis | Arm | Baseline Mean (SD) | LS Means (Std Err) | Within-group p | Between-group p | LS Means (Std Err) | Within-group p | Between-group p |
| PP | IP | 4.9 (4.7) | -0.7 (0.7) | 0.30 | 0.57 | -0.5 (0.3) | 0.16 | 0.49 |
|  | VW | 5.5 (5.1) | -1.1 (0.7) | 0.10 |  | -0.2 (0.4) | 0.52 |  |
| ITT | IP | 5.1 (4.7) | -0.6 (0.6) | 0.9 | 0.82 | -0.4 (0.3) | 0.23 | 0.45 |
|  | VW | 5.9 (5.3) | -1.0 (0.6) | 0.14 |  | -0.3 (0.3) | 0.31 |  |
| **PHYSICAL FUNCTIONING** ^c^ | | | Changes from baseline to 9 weeks | | | Baseline to 6 months | | |
| Analysis | Arm | Baseline Mean (SD) | LS Means (Std Err) | Within-group p | Between-group p | LS Means (Std Err) | Within-group p | Between-group p |
| PP | IP | 45.7 (9.5) | 2.3 (1.3) | 0.08 | 0.17 | 1.0 (0.7) | 0.11 | 0.69 |
|  | VW | 46.5 (9.0) | 1.0 (1.2) | 0.40 |  | 0.3 (0.6) | 0.64 |  |
| ITT | IP | 45.7 (9.2) | 2.7 (1.1) | 0.0192 | 0.09 | 1 (0.6) | 0.07 | 0.38 |
|  | VW | 46.1 (8.9) | 0.9 (1.1) | 0.39 |  | 0.5 (0.5) | 0.33 |  |
| **PATIENT ACTIVATION** ^d^ | | | Changes from baseline to 9 weeks | | | Changes from baseline to 6 months | | |
| Analysis | Arm | Baseline Mean (SD) | LS Means (Std Err) | Within-group p | Between-group p | LS Means (Std Err) | Within-group p | Between-group p |
| PP | IP | 67.5 (20.4) | 8.0 (2.8) | 0.0043 | 0.0235 | 1.6 (1.4) | 0.23 | 0.85 |
|  | VW | 68.8 (17.4) | 0.1 (2.5) | 0.95 |  | 0.7 (1.2) | 0.57 |  |
| ITT | IP | 66.5 (21.1) | 6.7 (2.5) | 0.0077 | 0.0121 | 1.7 (1.3) | 0.17 | 0.37 |
|  | VW | 66.0 (20.3) | 1.4 (1.4) | 0.55 |  | 1.3 (1.2) | 0.28 |  |
| **WEIGHT, lb** | | | Changes from baseline to 9 weeks | | | Changes from baseline to 6 months | | |
| Analysis | Arm | Baseline Mean (SD) | LS Means (Std Err) | Within-group p | Between-group p | LS Means (Std Err) | Within-group p | Between-group p |
| PP | IP | 193.2 (38.7) | 0.0 (5.6) | 0.999 | 0.0303 | -0.5 (2.8) | 0.87 | 0.46 |
|  | VW | 199.7 (45.5) | 0.8 (6.2) | 0.89 |  | -0.9 (3.1) | 0.77 |  |
| ITT | IP | 193.9 (39.6) | 0.3 (5.0) | 0.96 | 0.0223 | -0.5 (2.5) | 0.83 | 0.44 |
|  | VW | 196.1 (43.1) | 5.2 (5.3) | 0.33 |  | 1.2 (2.7) | 0.65 |  |
| **STEP COUNT** | | | Changes from baseline to 9 weeks | | | Changes from baseline to 6 months | | |
| Analysis | Arm | Baseline Mean (SD) | LS Means (Std Err) | Within-group p | Between-group p | LS Means (Std Err) | Within-group p | Between-group p |
| PP | IP | 9936.5 (3158.4) | 449.9 (480.1) | 0.35 | 0.0042 | -184.6 (222.0) | 0.41 | 0.17 |
|  | VW | 10060.1 (2938.9) | -547.0 (411.4) | 0.19 |  | -365.9 (202.8) | 0.07 |  |
| ITT | IP | 9831.2 (3283.3) | 312.9 (423.5) | 0.46 | 0.0053 | -128.7 (198.8) | 0.52 | 0.21 |
|  | VW | 9995.3 (3015.7) | -409.4 (366.3) | 0.26 |  | -212.7 (180.9) | 0.24 |  |

PP: Per Protocol; ITT: Intention-to-Treat; CI: confidence interval; IP: In-person; VW: Virtual World; MET: metabolic equivalent of task

^a^ Assessed using the Diabetes Distress Scale-17.^27,28^

^b^ Assessed using the Patient Health Questionnaire-8.^29^

^c^ Assessed using the physical function subscale on the PROMIS-29 measure.^30^

^d^ Assessed using the Patient Activation Measure (PAM) -13.^31^
